# Thirty years of drivers and patterns of land-use change across the Amazon biome

**DOI:** 10.1007/s13280-025-02199-5

**Published:** 2025-06-25

**Authors:** Diego Brizuela-Torres, Yves Zinngrebe, Mark Rounsevell, Calum Brown

**Affiliations:** 1 Department of Conservation Biology and Social-Ecological Systems, Helmholtz Centre for Environmental Research GmbH - UFZ, Permoserstraße 15, 04318 Leipzig, Germany; 2 Institute of Meteorology and Climate Research, Atmospheric Environmental Research (IMK-IFU), Karlsruhe Institute of Technology, Garmisch-Partenkirchen, Germany; 3 German Center for Integrative Biodiversity Research (iDiv) Halle-Jena-Leipzig, Deutscher Platz 5E, 04103 Leipzig, Germany; 4 Institute of Geography and Geo-Ecology, Karlsruhe Institute of Technology, 76131 Karlsruhe, Germany; 5 School of Geosciences, University of Edinburgh, Drummond Street, Edinburgh, EH8 9XP UK; 6 Highlands Rewilding Limited, The Old School House, Bunloit, Drumnadrochit, IV63 6XG UK

**Keywords:** Agricultural frontiers, Cropland expansion, Indigenous Territories, Mining, Protected Areas, Tropical deforestation

## Abstract

The Amazon biome is crucial for achieving global biodiversity and climate targets but is severely threatened by deforestation and land-use change. While direct deforestation drivers have been analysed, their interactions with socio-economic and land-use dynamics, and the effects of policy interventions remain poorly understood, partly due to limited long-term data. To address this, we present and provide a pan-Amazonian dataset of potential deforestation drivers for the period 1990–2020 and assess their main trends, emergent land-use archetypes, and links to socio-economic dynamics. Our findings reveal a general commoditization of deforestation frontiers—i.e. expansion of export-oriented crops and extractive activities, with regional particularities and different degrees of commoditization. Understanding large-scale patterns of land-use change is key to support policies that effectively address the shifting interactions between deforestation, land-use change, and socio-economic dynamics. These include agricultural and extractive industries’ expansion, migration and armed conflicts in the Amazon, and other frontiers of the Global South.

**Supplementary Information:**

The online version contains supplementary material available at 10.1007/s13280-025-02199-5.

## Introduction

The Amazon biome is a major component of the Earth system, and its ongoing deforestation and associated degradation are a major driver of global change (Vargas Zeppetello et al. 2020). The Amazon contains the largest and best-preserved tropical forest in the world, which hosts approximately one tenth of global biodiversity (European Commission—Joint Research Centre 2021) alongside a diversity of indigenous populations and human livelihoods (Pereira and Gebara 2022). As human impacts on the biome increase, feedback effects and tipping points may push much of the area into an alternate state with devastating effects locally and globally (Xu et al. 2022). Protected Areas and legally recognized Indigenous Territories, some of the most effective constraints on deforestation, are threatened by these same processes (Schleicher et al. 2017).

Cropland expansion is among the main direct drivers of tropical forest loss globally (Jayathilake et al. 2021). In the Amazon, expansion occurs in the form of extensive industrial crops (e.g. soy in Brazil (Fearnside 2017) and in Bolivia (Müller et al. 2014)) and as part of more complex colonization patterns in countries like Ecuador or Peru (Rojas Briceño et al. 2019; Viteri-Salazar and Toledo 2020). More recently, activities like mining (Giljum et al. 2022) or oil extraction (Viteri-Salazar and Toledo 2020) have become important drivers of deforestation. Impacts occur through clearance for extraction, infrastructure development, population growth, and immigration, some of which occur outside legal concessions (Giljum et al. 2022).

The large extent of unpopulated land in the Amazon has also served for decades as a receptor of populations that have emigrated from their home regions to seek new livelihoods. Official colonization and settlement programmes (Viteri-Salazar and Toledo 2020; Pokorny et al. 2021), as well as cross-national and subnational migration (Perz et al. 2005; Ichikawa et al. 2014), have created complex patterns of land-use change and contributed to the loss of millions of hectares of forest in the last decades of the twentieth century. Analyses of migration and capitals flow attracted by ‘available’ land and natural resources have largely been framed under frontier expansion theory (Ioris 2020). This constitutes a useful framework for analysing the land-use, demographic, agricultural, and resource extraction dynamics in the Amazon as well as in other regions of the ‘Global South’ (e.g. le Polain de Waroux et al. 2018). At present, the typical forces of frontier expansion—immigration and population growth, either officially directed or driven by conditions at origin places—are starting to be overcome by inflows of capitals linked to commodities’ global value chains (Barbier 2012) in a process named frontier ‘commoditization’ (Kröger and Nygren 2020).

Under novel globalized forces of frontier expansion and unprecedented cropland growth in tropical forests (Eigenbrod et al. 2020), it is imperative to understand their effects and interactions with the diversity of national and subnational social and economic dynamics (Jayathilake et al. 2021). Currently, drivers’ interactions and variation through time and across political demarcations are poorly understood (Pendrill et al. 2022), making it difficult to link the dynamics of direct drivers (e.g. when and where different crops or type of extractive activity expand) to the underlying forces of land-use change. In this context, policy interventions to control Amazonian deforestation have had limited long-term success (Kalamandeen et al. 2018), largely due to fragmented interventions that consider only single economic sectors or commodities, regions, or particular policy levers (i.e. lack of policy integration (Zinngrebe 2018)).

**Fig. 1:**
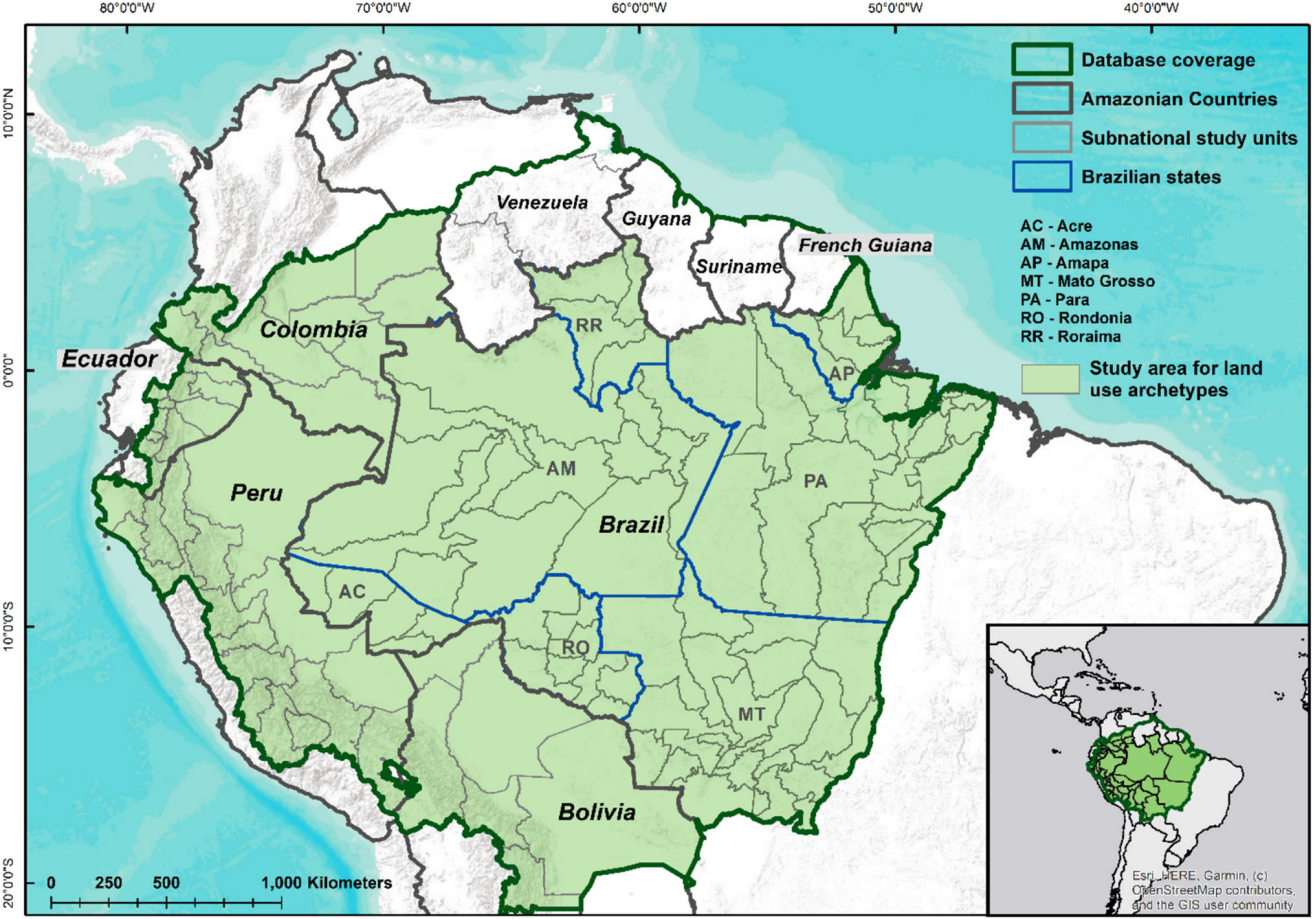
Map of Amazonian countries and the detailed study area. Available data were collected and processed for all Amazonian countries, but land-use archetypes analyses were performed only for those countries with sufficient subnational data

This fragmentation of interventions is, in turn, partly reflective of data and research operating within similar constraints, commonly precluding the characterization of land-use dynamics across time and spatial scales as well as the understanding of the relations between deforestation, direct and indirect drivers, and policy interventions aiming to control deforestation (Pendrill et al. 2022). To address these shortcomings, we developed a comprehensive, open-source dataset of potential deforestation drivers and interventions across the Amazon biome (Table 1) for the subnational demarcations displayed in Fig. 1. With this database, we tackle the following questions: How have potential deforestation drivers varied across time, countries, and subnational regions? Which drivers tend to vary together, where, and when? What land-use patterns (herein ‘archetypes’, see methods) do they drive? How are these outcomes affected by policy contexts?

**Table 1:** Drivers overview. Overview of the potential drivers of deforestation that were compiled and analysed. This table shows the general characteristics of different data types that were accessed and how these were processed. Detailed information and citations of each of the data sources are available in supplementary file 1 and supplementary material A. Outputs refer to the way drivers are expressed in final data products

Driver	Type of data	Sources	Processing	Output
Population	Tabulated, literature, documents, matricial spatial data (format: GeoTIFF)	Government statistics, scientific literature, demographic data compilations	1. Compilation of tabulated, documental and spatial data 2. Linear interpolation to complete yearly series 3. Calculate yearly % change	Total population and percentage population yearly change
Crops	Tabulated	Government statistics, FAO (French Guiana, Guyana and Suriname)	1. Compilation of tabulated data, mainly agricultural censuses and surveys 2. Harmonization of crops’ names 3. Calculate % area by subnational unit	Total area and percentage of subnational unit area covered by individual crops
Livestock	Tabulated	Government statistics, FAO (French Guiana, Guyana and Suriname)	1. Compilation of tabulated data of cattle, sheep, goat, pig and buffalo (Brazil and Colombia) 2. Calculate livestock density by subnational unit	Total livestock population, by species, and population density: population divided by subnational unit area in km^2^
Mining	Vectorial spatial data (format: ESRI’s shapefile), websites	Government repositories, public spatial data repositories, secondary sources	1. Compilation of spatial data 2. Compilation and addition of missing dates of concession granting 3. Categorization by type of mineral or scale of concession when possible 4. Calculate % area by subnational unit	Total area of concessions and percentage of subnational unit extent given to mining concessions
Oil	Vectorial spatial data (format: ESRI’s shapefile), websites, documents	Government repositories, public spatial data repositories, secondary sources	1. Compilation of spatial data 2. Compilation and addition of missing dates of oil block contract 3. Categorization by type of oil block (production or exploration) when possible 4. Calculate % area by subnational unit	Total area of mining blocks and percentage of subnational unit extent in oil blocks
Roads	Vectorial spatial data (format: ESRI’s shapefile), tabulated	Government statistics and data repositories	1. Compilation of tabulated and spatial data 2. Extraction of roads extension by subnational unit 3. Calculate roads density by subnational unit	Total length of main and secondary roads and roads density: Roads extension in km divided by subnational unit area in km^2^
Protected areas	Vectorial spatial data (format: ESRI’s shapefile)	Protected Areas polygons from government repositories, public spatial data repositories and World Database on Protected Areas (WDPA)	1. Compilation of spatial data 2. Compilation and addition of missing dates of Protected area establishment 3. Categorization by government administration level and use level allowed 4. Calculate % area by subnational unit	Total area and percentage of subnational unit extent declared as Natural Protected Areas
Indigenous territories	Vectorial spatial data (format: ESRI’s shapefile)	Indigenous territories polygons compiled by the Amazon Network of Georeferenced Socio-environmental Information (*Red Amazónica de Información Socioambiental Georreferenciada—*RAISG)	1. Compilation of spatial data 2. Compilation and addition of missing dates or Territories’ legal recognition 3. Calculate % area by subnational unit	Total area and percentage of subnational unit extent legally recognized as Indigenous Territories

We firstly describe the most important trends in the drivers and controls of deforestation, as well as the land-use archetypes that emerge. Then, using frontier expansion theory as a conceptual framework we characterize the different archetypes, relate them to ongoing deforestation processes, and discuss the socio-economic contexts in which they emerged. Lastly, we review the qualities and deficiencies of the dataset (gaps, consistency among different sources, potential documented and undocumented sources of uncertainty, etc.) and identify key information gaps in existent deforestation drivers’ data.

## Materials and methods

### Data collation and processing

Here we present an overview of the data sources, processing, and analytical approaches involved in the collation of the biome-wide dataset. In total, we compiled, curated, and processed 136 individual datasets from 33 different sources.

Detailed steps and a full list of data sources can be found in supplementary material A and supplementary file 1. Data and code for data processing and analyses can be found at:^1^.

### Study area

The study area is the Amazon biome, divided into political sub-units (Fig. 1). We used first-level subnational jurisdictions for Bolivia (*departamentos*), Colombia (*departamentos*), Ecuador (*provincias*), and Peru (*departamentos*). For Brazil, given the large number and small size of first-level subnational units (*estados*) that cover the Amazon biome, we used groups of municipalities named Microregions (*Microrregião*) (Instituto Brasileiro de Geografia e Estatística—IBGE 2025), while including data at municipality level as well. Subnational data were not available for some drivers and countries (Guyana, Suriname, and French Guiana), so we compiled national level data only in these cases. For Venezuela, which has about half of its territory in the Amazon, we only collected the available subnational data for the Amazonian states, which did not include crops or livestock, so we excluded Venezuela from the land-use archetypes analyses. In total, our study area covers 8 142 726 km^2^. The main analyses presented below are at subnational resolution and so focus on the 128 study units at this resolution, covering 7 236 723 km^2^. Supplementary material A contains full details on the definition of the study area and a list of all the national sub-units included.

### Data obtention and processing

We collected data of human population, cropland areas of individual crops, livestock populations, oil blocks, and mining concessions—as well as Protected Areas and Indigenous Territories as potential counter-deforestation interventions—and produced yearly time series. We include human population as an indirect driver, given its importance for frontier expansion dynamics. We obtained these data from governmental data repositories, non-governmental organizations, and cross-institutional collaborative efforts. Some drivers with different definitions in different countries (Protected Areas, mining concessions and oil blocks) were re-categorized into simpler categories for further analyses. Table 1 shows an overview of data characteristics and processing.

### Assessment of data characteristics

A comprehensive analysis of data quality was not possible for the entire dataset because full metadata, documentation, or alternative data were not available in many cases. Instead, we performed a qualitative assessment of data quality, recording data structures and formats, documentation, type of data (e.g. compilations, surveys, census, estimates, spatial data, data from scientific literature, and other compilatory sources), number of distinct sources, etc. We considered how these characteristics did and could hamper analysis, and provide suggestions for transparent acknowledgement and treatment of any limitations that we identified. Additionally, we included a rapid benchmarking exercise by comparing the magnitudes and trends emerging from our data to those emerging from the data compiled by the NGO MapBiomas (MapBiomas 2024). See supplementary material D for details about this comparison.

### Crops data harmonization

Given the large number of crops produced in different countries, the variety of local names and variation in naming conventions in different data sources, crops data required substantial harmonization. We harmonized the crops database with unique common names in English (when possible) across different countries. In total, the data include approx. 200 different crops (Some could not be fully identified.). See details about crops data harmonization in supplementary material A.

### Deforestation drivers and trends

**Fig. 2:**
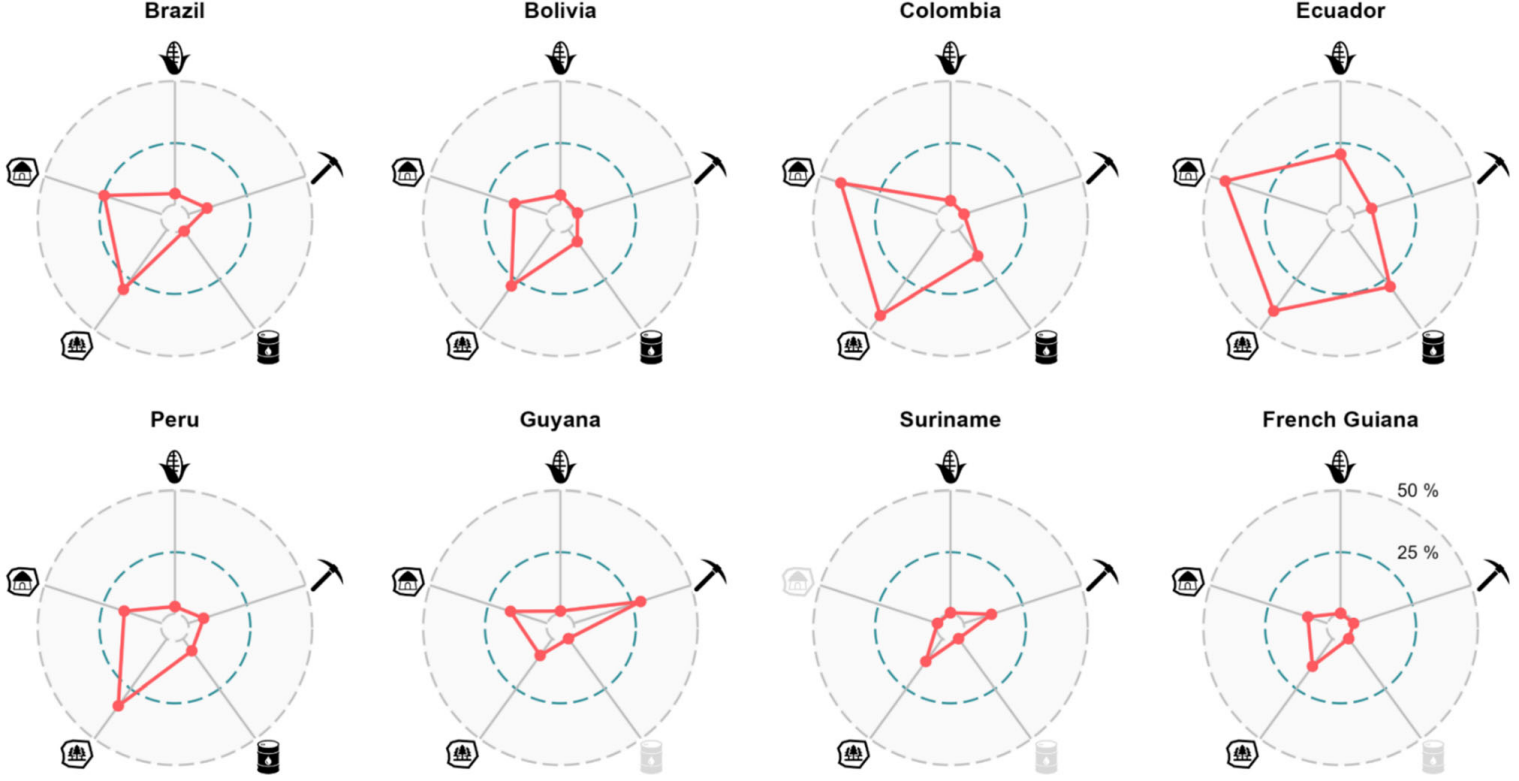
Total percentage area covered by 2020. These plots show the total percentage area covered by the drivers that had explicit area units (i.e. excluding population, livestock population, and roads) for all countries except Venezuela. Considerable overlap between Protected Areas of different categories, Indigenous Territories, mining concessions, and oil blocks was detected. Hence, in cases such as Ecuador where the largest overlaps were detected, the total area covered by these drivers sums up to more than 100%. The year displayed in Peru is 2019, due to gaps in the 2020 crops data, and in French Guiana it is 2006 as that is when crops data end. We excluded Venezuela from this figure due to the large amount of overlap in the Protected Areas data that were difficult to understand due to the lack of secondary information and to the lack of subnational crops data. Guyana, Suriname, and French Guiana had no data about oil blocks. Suriname has no legally recognized Indigenous Territories

We plotted yearly time series of different drivers across subnational units and used them to visually compare the trajectories of drivers across time, and to complement our analyses of land-use archetypes. These plots are available in supplementary material B. We present the total change (percentage increase or decrease) per decade in Table 2, where we show the changes in main crops (soy, corn, rice, oil palm, cacao, coffee, sugarcane, and banana) and cattle separately given their relevance in deforestation processes. In Fig. 2, we show the total proportional area covered by drivers and interventions with area units (agriculture, mining, oil, roads, Protected Areas and Indigenous Territories) by the year 2020. We also plotted main crops, cattle, and the rest of the drivers and interventions grouped by archetype (see below) and tested the significance of observed trends (see Sect. "Statistical analysis"). Plots for the main crops at the archetypes in deforestation frontiers are shown in Fig. 5 and for the rest in supplementary material E.

**Table 2:**
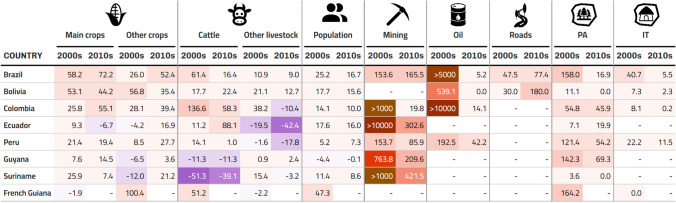
Drivers’ percentage change by decade. Total percentage change per group of drivers for the decades of the 2000s and 2010s*. The period for these decades is 2000–2009 and 2010–2019, respectively. Purple cells show areas where percentage decreased and red cells where it increased along the corresponding decade. The main crops in this table include soy, corn, rice, oil palm, cacao, coffee, sugarcane, and banana

*Due to different data gaps, especially in the earlier years of the 1990–2020 period, these percentage changes correspond to slightly different time periods for different countries. Namely, in Ecuador, crops area and livestock density correspond to the periods 2002–2010, 2011–2020. This was done to calculate percentage changes along the ESPAC dataset (see supplementary material A) which starts in 2002. In Bolivia, oil data start in 2006; hence, periods are 2006–2016 and 2017–2020. In Brazil, roads data were only updated by decade; hence, to capture the change from one decade to the other the periods are 1999–2008 and 2009–2019. In Colombia, oil data start in 2001, so periods are 2001–2010 and 2011–2020. In French Guiana, most data are not available after 2006, so the only period displayed is 1996–2006

### Subnational land-use archetypes

Having collated and harmonized the data, we used principal components analysis (PCA) and k-means clustering (Sect. "Statistical analysis") to identify groups of subnational units with distinctive patterns in drivers’ variation at different time steps, which we named subnational land-use archetypes. We did this analysis for all of the countries for which we found subnational data for all or most drivers: Brazil, Bolivia, Colombia, Ecuador, and Peru. We characterized emergent archetypes, compared them qualitatively across countries and time steps with respect to the socio-economic contexts likely causing their emergence, and discuss how they form frontiers with different degrees of commoditization.

### Statistical analysis

In analysing drivers of deforestation (Sect. "Deforestation drivers and trends"), we performed a Mann–Kendall analysis to test the significance and strength of the trends observed within different archetypes for the main crops, cattle, population growth, and extractive activities. For the PCA analysis described in Sect. "Subnational land-use archetypes", we used drivers’ data expressed as percentage cover area for crops, mining concessions, oil blocks, Protected Areas and Indigenous Territories, density for livestock and roads, and annual percentage growth rate for human population. We applied PCA approximately at five-year intervals (avoiding years with severe data gaps where necessary) and produced biplots to display drivers and subnational units’ separation across the first two principal components. In addition to visual identification of the main patterns, we used k-means clustering to identify clusters of subnational units, choosing the number of clusters to co-maximize visual separation and coverage. Then, we mapped these clusters with the polygons of subnational units. Although we acknowledge that specific socio-economic or political events relevant to deforestation have occurred at different times in different countries, we chose 5-year intervals to offer an overview of subnational land-use archetypes at the same time slices across the Amazon. We performed these analyses for each country as well as for all countries together (i.e. pan-Amazonian analysis), but since the percentage of variance explained was considerably higher for individual country analyses than when analysing all countries together, the results we present here correspond to analyses for each country separately.

We used the statistical programming environment R (R Core Team 2021) to perform all analyses and the R package factoextra (Kassambara and Mundt 2020) to produce biplots.

## Results

We found that relevant data on deforestation drivers are widely scattered and inconsistent. Therefore, the dataset presented here is the result of compiling, curating, and harmonizing tens of individual datasets. The temporal extent and availability of the processed dataset are described in Table S2 in supplementary material A, and the entire formatted dataset can be accessed at 2. Below, we present the main characteristics of this dataset, followed by analyses of subnational land-use archetypes.

### Trends through time

Nearly all of the drivers analysed increased substantially from 1990 to 2020, across all Amazonian countries. The areas of mining concessions had the largest proportional increase in all the drivers analysed (see Fig. 2), growing at least two-fold in the last two decades in all the countries where data were available (see Fig. 2)—although from very low starting points in most countries. For instance, in the Ecuadorian Amazon there were only approx. 17 km^2^ of mining concessions in 2000. By 2020, there were approx. 11 007 km^2^ (about 3% of Ecuador’s area). For the countries with data on oil blocks (Bolivia, Brazil, Colombia, and Peru), we also found large increases during the 2000s and early 2010s (see Table 2 and supplementary material B).

Cropland—particularly soy and corn, which are sometimes grown in rotation—has also expanded considerably. The area dedicated to soy is especially large in Brazil and Bolivia and remains far higher than the area of any other crop. In Brazil, cattle farming has greatly expanded following the trajectory of soy and corn. Also in Brazil, the açaí palm (*Euterpe oleracea*) fruit has become the most harvested fruit, more so than oil palm, cacao, or coffee, although data for this fruit are only available since 2015. Ecuador and Colombia had the slowest increases in cropland area. Cattle populations grew strongly in Bolivia, Brazil and Colombia, but decreased in Ecuador, Peru, Guyana, and Suriname.

Total human population increased in most of the subnational jurisdictions (except in Guyana, where total population decreased) across the study period, although growth rates deaccelerated in all countries. In general, the subnational jurisdictions towards the interior of the Amazon had the largest population growth rates.

Meanwhile, Protected Areas and Indigenous Territories currently occupy the largest proportional area in most of the countries (Fig. 2). Protected Areas had, in general, large increases in the first two decades of analysis, but nearly stagnated since the 2010s (with the exception of Peru). Indigenous Territories showed similar trends where present (Suriname had none recognized by 2020), although some Brazilian microregions and the Peruvian department of Ucayali still had large increases throughout the early 2010s. We detected different levels of overlap, particularly high in Ecuador, between Protected Areas and Indigenous Territories with mining concessions and oil blocks.

### Subnational land-use archetypes

**Fig. 3:**
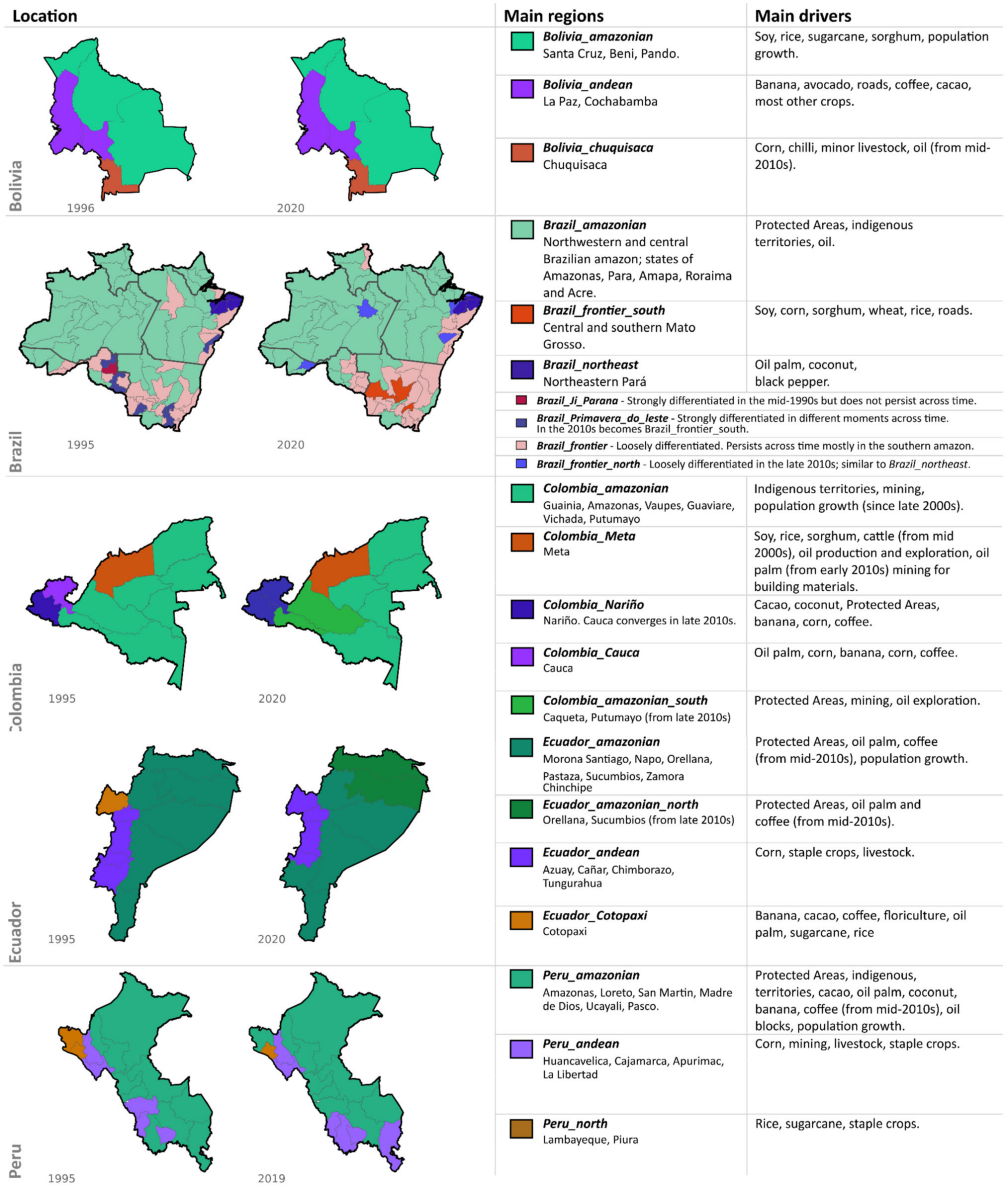
Land-use archetypes by country. Land-use archetypes (clusters across principal components 1 and 2) for the earliest and latest years available per country. The main regions’ column shows the names of the study units that conform to each archetype, and in the main drivers’ column, the variables that most strongly correlate with the study units clustered in that archetype, and which most strongly drive Principal Components 1 and 2. In the case of Brazil, we provide a short description of the lesser relevant archetypes (less differentiated and less consistent through time) that are shown in the maps

**Fig. 4:**
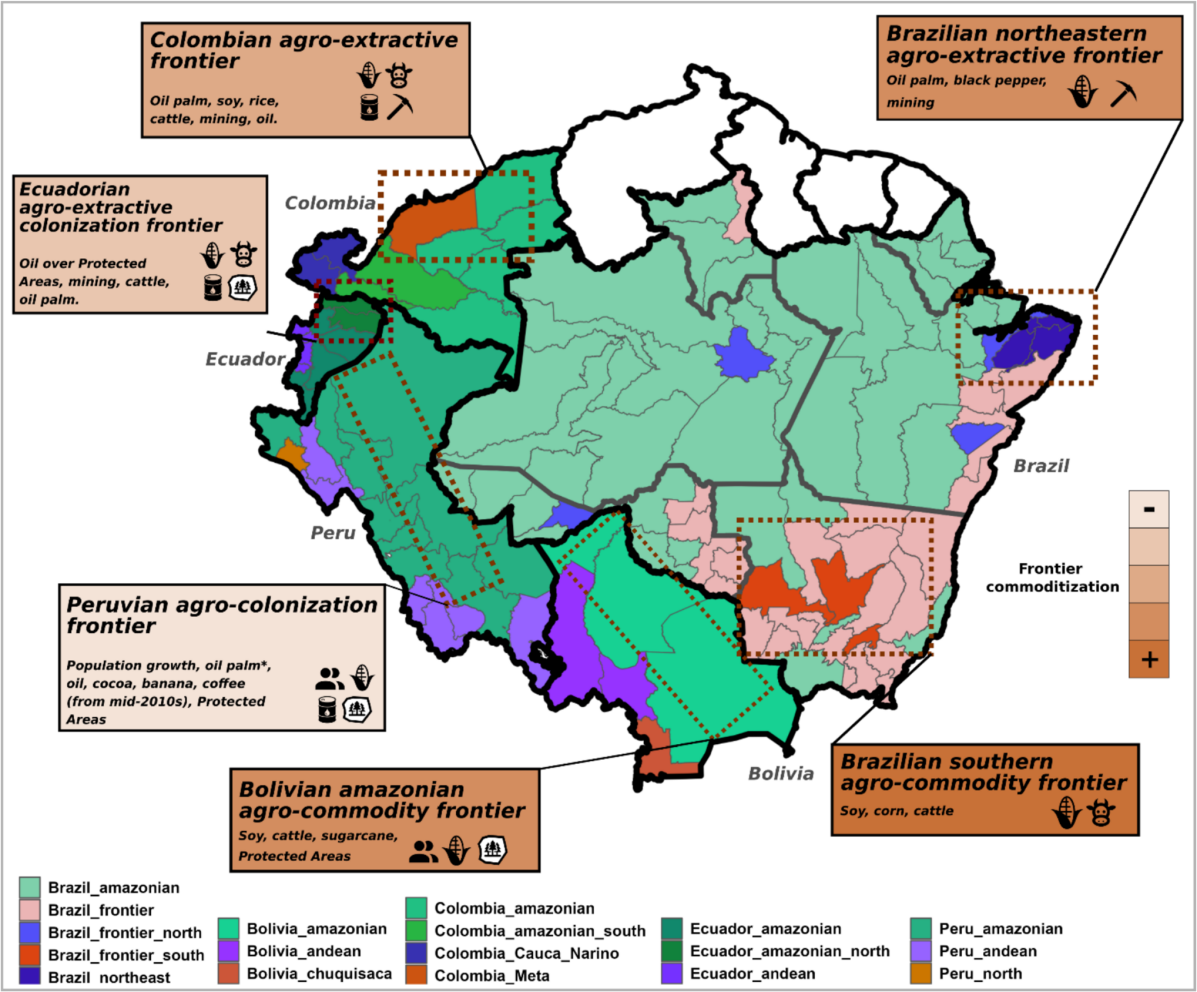
Map of subnational land-use archetypes and frontiers in 2020. This map shows the spatial distribution of subnational land-use archetypes by 2020. In the dotted rectangles, we indicate the regions where archetypes form the main frontiers. In the textboxes, we indicate the drivers that dominate the archetypes at these frontiers. Text boxes are coloured according to the qualitative grading of commoditization. This map is produced from the PCA and k-means outputs from each country. *In Peru, oil palm production areas are mostly located in the northern Amazonian departments of San Martin and Ucayali

**Fig. 5:**
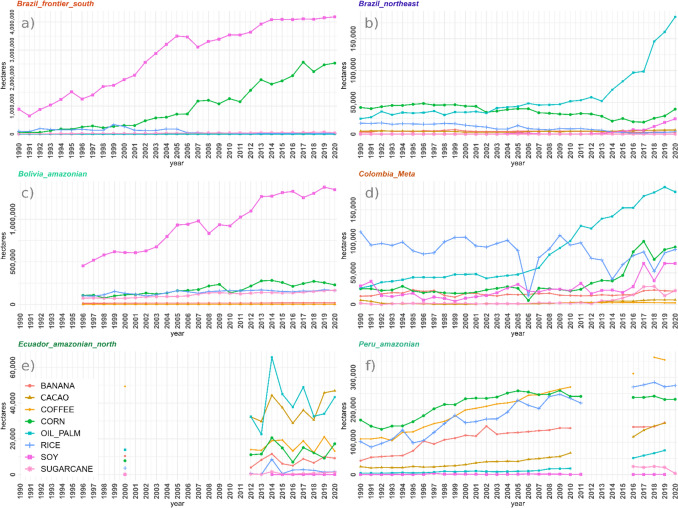
Main crops aggregated by archetypes. These plots show the main crops aggregated by the subnational land-use archetypes that emerge by 2020 in the main agricultural frontiers shown in Fig. 4. The plots also show the years with missing data for these crops along the 1990–2020 time period

**Fig. 6:**
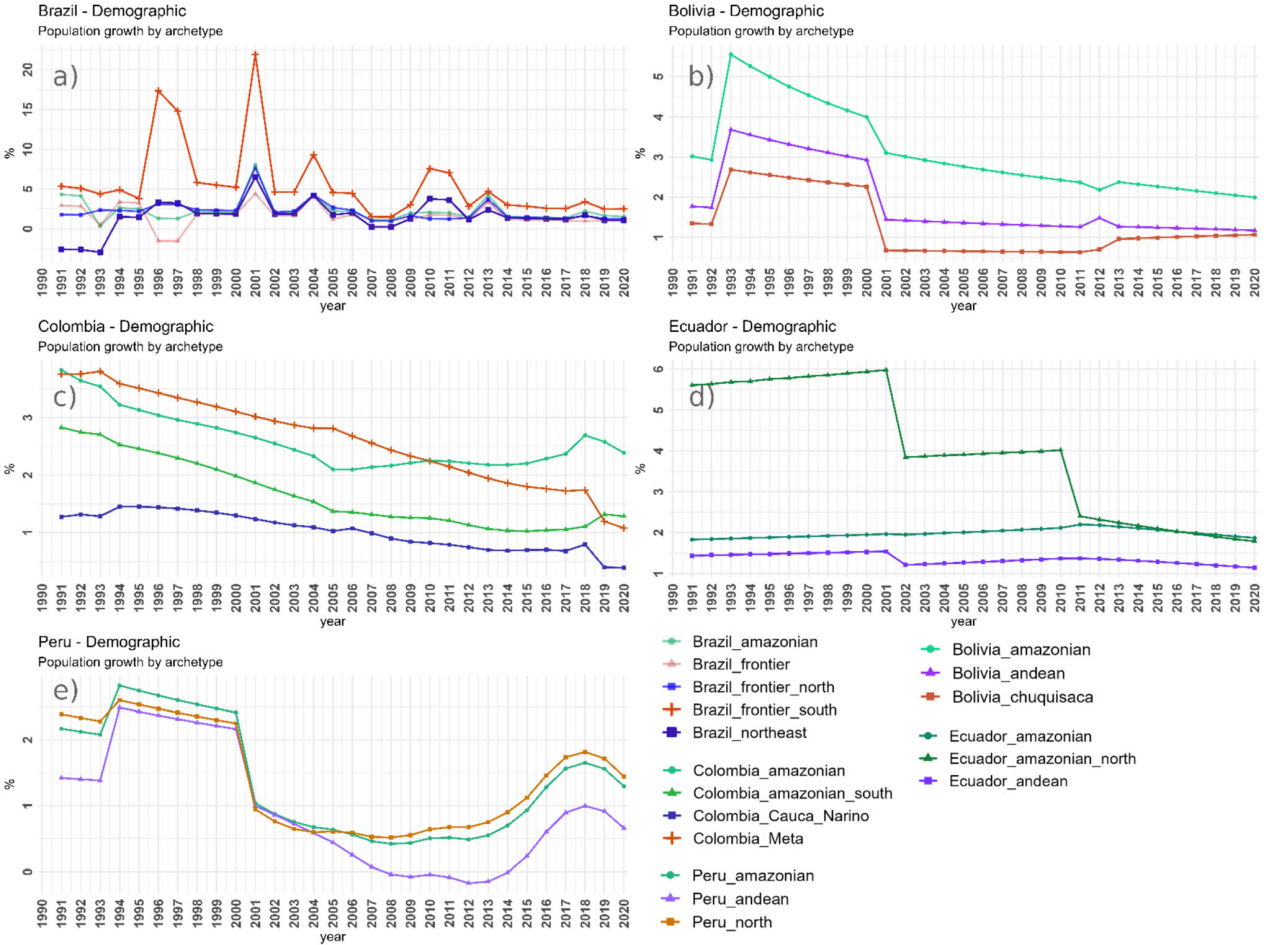
Population growth by archetypes. These plots show the changes in population growth rates aggregated by the subnational land-use archetypes that emerge by 2020

Subnational land-use archetypes and the times at which they emerge had important country-specific characteristics (see Fig. 3 and supplementary material C). Yet, they also share a trend towards increasing dominance of cash crops and extractive activities alongside a slowing in population growth. At present, the interplay of these general trends with regional socio-economic and political processes at different countries and subnational jurisdictions has given place to frontiers with diverse levels of commoditization, as shown in Fig 4. Figures 5, 6, and supplementary material E show the time series of the expansion of main crops, cattle and extractive activities. Supplementary material F shows the outcomes of the Mann–Kendall analyses, which find statistically significant trends in different countries as described in the following sections.

#### Brazil

The *Brazil_amazonian* covers most of the Brazilian Amazon. It is mainly influenced by the presence of Protected Areas of different categories and Indigenous Territories, has few major roads, and is the only archetype containing oil blocks. From the mid-1990s, several microregions diverged, mostly in the southern and south-western borders of the Brazilian Amazon, to eventually develop a markedly distinct archetype, *Brazil_frontier*. This archetype is driven by crops like coffee or cacao, and population growth and livestock at different moments in time. From the early 2000s, several microregions from the southern Amazon diverged further to form a distinct archetype (*Brazil_frontier_south*) strongly dominated by soy and corn. Other important temporary crops such as rice and sorghum also influence this archetype. The microregion of Primavera do leste, in the state of Mato Grosso, is highly representative of this archetype, to the extent that at different points in time it diverged from *Brazil_frontier_south* towards a stronger influence of the crops driving this archetype (i.e. soy, corn, sorghum). An independent *Brazil_northeast* archetype, driven by commodity crops like oil palm, coconut, black pepper, and tobacco also emerged in the northeastern Amazon. By 2020, the *Brazil_northeast* and the *Brazil_frontier_south* archetypes were the most differentiated and consolidated.

#### Bolivia

In Bolivia, three archetypes emerged and remained stable through time. The *Bolivia_amazonian* archetype covers the Bolivian lowland (i.e. Amazonian) departments of Santa Cruz, Beni, and Pando and is dominated by population growth and commodity crops such as soy (by far the most planted crop), rice and sorghum, as well as Protected Areas and Indigenous Territories. An inversely correlated archetype, *Bolivia_andean*, is influenced by nearly all crops other than commodity temporary crops, by roads and, to a lesser extent, oil blocks. Finally, the department of Chuquisaca diverged strongly from the 1990s. The *Bolivia_chuquisaca* archetype is marked by an absence of both agroindustry (contrary to *Bolivia_amazonian*) and infrastructure development (contrary to *Bolivia_andean*), instead comprising corn, wheat, and other minor crops, as well as minor livestock and, from the early 2010s, oil exploration.

#### Colombia

Most Colombian departments belonged to the *Colombia_amazonian* archetype. This archetype is strongly dominated by Indigenous Territories, but population growth and mining, mostly gold, became increasingly influential from the early 2010s. The departments of Meta, Cauca, and Nariño all formed archetypes of their own. *Colombia_Meta* was strongly dominated by soy, rice, cattle since the mid-2000s, and oil palm since the early 2010 (see Fig. 5 panel *d*) and has experienced a substantial decrease in population growth. *Colombia_Nariño* was dominated by staple food crops such as corn and potato, with other economically important crops such as cacao and banana becoming important from the mid-2000s. *Colombia_Cauca* was strongly dominated by oil palm—by far the most-planted crop in the Colombian Amazon—from the mid-1990s until the late 2000s (when oil palm started expanding more in Meta, see Fig. 5), and to a lesser extent by sugarcane.

#### Ecuador

In Ecuador, three main archetypes emerged. The *Ecuador_amazonian* archetype was consistently dominated by population growth, and to a lesser extent by Protected Areas. By 2017, the year at which oil blocks data was available, the provinces of Orellana and Sucumbios, which belong to this archetype, had the largest oil blocks’ area. The departments of Chimborazo, Tungurahua, Cañar, and Azuay formed the *Ecuador_andean* archetype, dominated by pastures, livestock, corn, and other staple crops such as potato, beans, and wheat. By the end of the 2010s the departments of Cañar and Azuay had strongly diverged from this archetype). Throughout the 2000s, the department of Cotopaxi clearly and strongly diverged from the rest of the country, mainly due to the planting of cacao, banana, and rice, the most-planted crops in Ecuador. Sugarcane, oil palm and coffee also became important in Cotopaxi in the mid-2000s. Mining concessions and ornamental flowers (floriculture is an important agricultural sector in Ecuador) were also dominant in the early 2000s; after this point, subnational data for flowers were not available and mining expanded into other areas as well as into Cotopaxi.

#### Peru

We detected three main archetypes in Peru. The *Peru_amazonian* archetype, highly represented in the departments of San Martin, Madre de Dios, Ucayali, and Loreto, was dominated by population growth, but also by Indigenous Territories and Protected Areas. Some of the most economically important crops like cacao, oil palm, and banana were associated with this archetype, as well as coffee from the early 2010s. Oil palm was also present and strongly correlated with population growth throughout the mid-1990s and 2000s, particularly in San Martin. From the mid-2000s, areas under oil exploration and exploitation expanded in this archetype. Inversely correlated was the *Peru_andean* archetype. It is dominated by cattle and staple crops such as potatoes and corn, with mining becoming important by the early 2000s. This archetype was most prevalent in the departments of Apurimac and Huancavelica, but also occurred in La Libertad and Cajamarca until the mid-2000s. After this, La Libertad and (temporarily) Cajamarca became associated with the third archetype, *Peru_north*. This archetype is mostly independent of the other two and remained well defined until the mid-2010s in the northern departments of Piura and Lambayeque. It includes rice and sugarcane.

### Assessment of data characteristics

There was major heterogeneity in data formats, accompanying metadata or documentation, temporal extents, and consistency between, and in some cases within countries. In general, human population data were the most complete and homogeneous. Livestock population data, particularly for cattle, were also relatively complete, but had to be compiled from different sources for most countries. Crops data required the most processing, being the largest in volume and also the most heterogeneous among and within countries. Crops’ names required substantial harmonization, for which details are provided in supplementary material A and supplementary file 2. For spatially explicit data, the challenges were mostly related to the information content in the vectorial spatial data (format: ESRI’s shapefile). Mining concessions and oil blocks files did not contain timepoints for some countries, but these were often available elsewhere and so were incorporated manually (see supplementary material A and supplementary file 1 for details on spatial data sourcing and processing).

The rapid benchmarking of the dataset collected here with MapBiomas had to be largely visual (i.e. comparing plots; see supplementary material D) and qualitative given important differences between both datasets: for instance, different origins (e.g. crops area statistics reported by countries vs remote sensing), different forms of aggregation (e.g. individual crops’ area vs. total cropland area), or different variables (e.g. polygons of mining concessions vs areas affected by mining detected from remote sensing). However, the general patterns of expansion of cropland, commodity crops (for the case of palm oil), and mining operations (expansion of concessions and area affected) emerge from both datasets. See supplementary material D for all results of this comparison.

## Discussion

The dataset and analyses we present reveal a general trajectory of frontier commoditization across the Amazon, with several archetypes increasingly dominated by export-oriented crops, hydrocarbon and mineral concessions, whereas population growth deaccelerates. This general trajectory, nevertheless, varies in intensity, stage, and other particularities among countries and subnational land-use archetypes. We now discuss the spatial and temporal dynamics of the land-use archetypes across countries, the socio-economic contexts in which they emerge, and situate the frontier commoditization trend we observe in the Amazon, in the context of global commodity markets. Lastly, we reflect on the potentialities and limitations of the dataset collated here for understanding long-term land-use dynamics across the Amazon.

### Frontiers expansion and commoditization

With different intensities, paces, and particularities, all countries analysed show signs of commoditization. As in other biomes shared by different countries (e.g. the Gran Chaco (le Polain de Waroux et al. 2018) or the Congo Basin (Ordway et al. 2017)), we show that frontier expansion and commoditization across the Amazon are mediated by socio-economic dynamics that vary at national and subnational scales. At present, this has originated frontiers with varying degrees of commoditization across different subnational land-use archetypes. Figure 4 shows the geographic distribution of archetypes by 2020 and a qualitative grading of frontier commoditization according to the relative dominance of commodities vs. population growth.

In Brazil, the *Brazil_frontier_south* and *Brazil_northeast* archetypes exhibit a strong commoditization earlier than other Amazonian areas, which persists until present (see Brazil in Fig. 3). These archetypes have formed frontiers across the Amazonian ‘Arc of deforestation’ (Brazil’s south and northeast ‘agro-commodity’ frontiers in Fig. 4), dominated by commodity crops since the early 2000s—permanent crops like oil palm or coconut in the northeast and temporary crops like soy and corn as well as cattle in the south, whereas population growth has slowed down (see Figs. 5 and 6 panel *a*). While these demographic changes may indicate changes in crops production and land tenure dynamics (e.g. mechanization and land consolidation, with less people managing more land), they may also reflect large reductions in fertility rates over the second half of the twentieth century (Nascimento Lombardi and Luíz do Carmo 2020). However, soy undoubtedly reflects a strong commoditization as it has been closely linked to the agroindustry since its first introduction in this region (Cattelan and Dall’Agnol 2018).

The more recent expansion of corn is also linked to agroindustrial development. The cropping of soy and corn in rotation made Brazil one of the major exporters of these crops globally (Cattelan and Dall’Agnol, 2018). This also prompted increases in the supply of animal feed, making Brazil the main exporter of meat and poultry (Klein and Luna 2022), further linking land-use in this frontier to global value chains and inducing pervasive dynamics of land grabbing and speculation, commonly linked to cattle ranching and illegal logging (Fearnside 2017). Although soy was partially decoupled from deforestation in Brazil during the 2010s, mostly through the Amazonian Soy Moratorium (Villoria et al. 2022), political changes occurring near the end of our dataset (the election of president Bolsonaro in 2019) have likely attenuated the effect of the Moratorium (Barbosa de Andrade Aragão et al. 2024).

In Bolivia, soy shows a nearly constant growth in the *Bolivia_amazonian* archetype since the 1990s, with a similar expansion curve as in the neighbouring *Brazil_frontier* and *Brazil_frontier_south* archetypes. Despite estimates of minimal transnational deforestation leakage to Bolivia from deforestation control policies in Brazil (Villoria et al. 2022), Brazilian agroindustrial investments have expanded substantially in Bolivia (Mackey 2011). At present, conflicts have emerged between the pro-environmental and pro-indigenous discourse that gained relevance since Evo Morales’ arrival to the presidency in 2006, and new advantages given to the national and foreign agroindustry, as well as unabated deforestation (Castro Pereira and Viola 2021). This circumstance is likely to allow further expansion of soy—and associated deforestation—in this archetype.

In Ecuador, data for oil blocks’ contracts were not available, but oil is known to have been an important driver of colonization for decades (Sierra 2000; Viteri-Salazar and Toledo 2020) and plays a major role in Ecuador fiscal income (Fernández Moreno et al. 2024). At present, nevertheless, our analyses suggest that oil expansion is less related to migration. For instance, the provinces of Sucumbios and Orellana where the *Ecuador_amazonian_north* archetype emerges have the largest proportion of oil blocks, and at the same time the smallest population and largest decreases in population growth since the 1990s (Fig. 6, panel *c*). Plus, similarly as Codato et al. (2019), we found large overlaps of oil blocks with Protected Areas and Indigenous Territories in this region.

In Colombia, the Colombian Armed conflict has shaped demographic, economic, land tenure, and land-use dynamics across many regions of the study area (Suarez et al. 2018; Lozano Ospina et al. 2021). This is reflected, for instance, in the expansion of commodity crops like oil palm, cattle, or oil extraction in the *Meta* archetype—despite deaccelerating population growth (Fig. 6, panel *c*)—after violence from armed conflicts was reduced in the mid-2000s due to strong governmental intervention (Grajales 2020). This aligns with an observed preference in these industries for less populated areas to avoid conflicts over land (Murillo-Sandoval et al. 2021). On the other hand, low population is a limitation for developing permanent commodity crops which require labour (Fontanilla-Díaz et al. 2021). In any case, the post-conflict land tenure and social dynamics have a strong influence on the actors that benefit the most from the commoditization of this frontier and are likely to keep influencing future dynamics (Grajales 2020).

In the Peruvian Amazon, commoditization is the lesser advanced, and agricultural frontiers are still largely pushed by migration (Peruvian agro-colonization frontier in Fig. 4). Armed conflicts, poverty, and lack of access to land have prompted migration, particularly from the Andes, towards the Amazon in different waves across the decades (Menton and Cronkleton 2014). This migratory process is reflected in the divergence in population growth rates among *Peru_Amazonian* and *Peru_Andean* archetypes in the early 2000s. Upon arrival, migrants commonly adopt the ‘migrant farming’ cycle, clearing patches of forest across the landscape to cultivate temporary staple crops. (Ichikawa et al. 2014). This is known to be an important factor for deforestation in Peru (Menton and Cronkleton 2014), although the actual magnitude of impacts is debated (Ravikumar et al. 2017).

The expansion of commodity crops like oil palm (in the northern departments) and cacao in the *Peru_amazonian* archetype since the mid-2000s also emerges from the dataset. Although far behind from the main crops in the region (rice, corn and coffee), oil palm expansion exercises a strong influence in land-use dynamics given its links to global value chains and the increasing demand for vegetable oils (OECD and FAO 2021). In Peru, oil palm-producing areas have typically belonged to smallholders since coca substitution campaigns in the 1990s and 2000s (Borasino 2016). However, the palm sector is going through a process of industrialization, with increasing influence of large producers and agroindustry companies, increasing the risk of becoming an important deforestation driver (McCarthy 2020).

### Amazonian commoditization in the global context

Globally, most tropical deforestation is linked to agricultural production for domestic demands (Pendrill et al. 2022). In contrast, frontier expansion in the Amazon and other regions of Global South is increasingly linked by the influence of global demands for commodity crops and industrial materials (Kröger and Nygren 2020). Amazonian frontier expansion occurs in interaction with diverse demographic dynamics, social conflicts, market access conditions, land tenure regimes, and governmental interventions that, despite being driven by the same global forces, result in land-use dynamics that differ to other regions of the globe.

For instance, in Bolivia and Brazil, soy expansion is driven by cross-border commercial integration, whereas in Indonesia and Malaysia, oil palm expansion reflects border security efforts (Eilenberg 2014). In sub-Saharan Africa, cash crop growth is led by small and medium producers who are increasingly linked to global markets, rather than the large agroindustrial or governmental investments that drive similar outcomes elsewhere (Ordway et al. 2017). In regions with contrasting political histories, the reopening of frontiers by global market demands take yet other forms. In post-Soviet Eurasia, cropland abandonment and weak agricultural investment after the USSR’s collapse created unique land tenure systems and perceptions of ownership (Petrick et al. 2013). Unlike the Amazon, these conditions made large agricultural investments desirable for a large part of the population, demonstrating how factors like land tenure and government support mediate global market impacts (Meyfroidt et al. 2016).

Our findings also indicate that future studies based on statistical data on drivers and framed in frontier expansion theory offer a potential to explore many local or regional processes of frontier expansion, with relevance beyond Amazonia and across the Global South, where a growing body of evidence points to the rapid commoditization of many agricultural frontiers (Barbier 2012; le Polain de Waroux et al. 2018; Kröger and Nygren 2020). In combination with in-depth studies on the social dynamics and governance options related to different agricultural frontiers, these statistical studies could tackle further research avenues regarding, for instance, key levers for the regulation and mitigation of social and environmental impacts, national and transnational deforestation leakages, or the magnitude of deforestation attributable to regional, national, or global market forces.

### Land-use governance in telecoupled commodity frontiers

Deforestation frontiers in the Amazon reflect a combination of subnational, national, and global factors, with the latter becoming increasingly relevant. Furthermore, deforestation itself is a multifaceted problem, with causes and impacts that cross several economic and policy sectors (McElwee et al. 2024). We have shown that, for instance, agricultural commodities and extractive activities interact with different demographic and other land-use dynamics in the jurisdictions where they occur. It is hence key to mainstream deforestation across all policy sectors involved (e.g. agricultural, extractive, rural development) (Runhaar et al. 2024). Depending on the specific characteristics of a region, deforestation might need to be mainstreamed differently. For example, in the Peruvian Amazon, where signs of commoditization are weaker than in other frontiers of the Amazon, halting deforestation would require a mainstreaming strategy that approached rural development and migration, along with other sectors more directly related to land use like agriculture, forestry, or infrastructure development (Zinngrebe 2018).

Amazonian deforestation also crosses several administrative levels, conditioning the effectivity of interventions to the degree of integration across different policy and administrative levels. Across the Amazonian countries, policy and administrative integration is heterogeneous (Castro Pereira and Viola 2021). While some countries like Brazil—albeit not free of failures and setbacks (Trancoso 2021)—have made important progress in the last decades integrating policy, private commitments, and administrative structures (Costa Neves and Whately 2016; Sills et al. 2020), other countries like Peru have had a long and incomplete administrative decentralization process that has hindered their capacities to tackle deforestation (Castro Pereira and Viola 2021). For instance, problems like uncertainty in the responsibilities of different agencies, the existence of contradictory incentives, and difficult access to rural development programmes due to excessive bureaucracy largely arise due to poorly integrated capacities after administrative decentralization (Kowler et al. 2016). Yet, subnational cases of more effective land-use governance through improved integration also exist in Peru. Here, the Region of San Martín, implemented a series of institutional and legislative actions throughout the late 2000s and early 2010s that allowed a drastic reduction in deforestation rates and the establishment of land-use governance structures that until today contribute to the comparatively better environmental performance of San Martin (Augusto Meléndez et al. 2017).

Finally, as value chains of key exports become more globally interconnected (i.e. telecoupled (Newig et al. 2020)), it is important that initiatives spanning different levels of political organization—local, regional, national—as well as with global private commitments and standards, become coherently interconnected. For instance, the European Union Deforestation Regulation (European Union 2023) could serve to align land-use governance towards reduced deforestation in countries wishing to increase their exports to the European Union.

### How useful are the available data?

We found important differences in the degree of availability, systematization, metadata, and documentation of data across countries. In general, Bolivia and Brazil had the most consistent and well-documented data. For crops and livestock data, both countries have complete subnational time series that extend over the entire study time frame as well as data on the temporal evolution of road networks. Dates of mining concession contracts were the only major gap in Bolivian data. Peruvian and Brazilian mining data were available directly from official sources but only from external sources in other countries. Although the validity of data obtained from non-official sources was assessed by contrasting it to similar but incomplete datasets from reputable sources (e.g. the spatial data compiled by MapBiomas), caution should be exercised as relevant updates or additions might be missing. Only French Guiana had no mining data available at all, although it is documented that both legal mining (Columbus Gold Corporation 2015) and illegal mining (Hammond et al. 2007) occur in this overseas French territory. The lack of access to dates of oil blocks establishment in Ecuador was another major gap. Given the importance of oil in the Ecuadorian economy and the role of oil in Ecuadorian Amazon land-use dynamics (Sierra 2000), locating the establishment of oil blocks in their temporal context would provide a more detailed vision of the development of this industry. Consistent, multi-temporal data on illegal mining are another significant data gap (Giljum et al. 2022), as well as road expansion and spatially explicit data of individual crops and livestock breeding areas.

Another important limitation in the dataset is the scarcity of data for Suriname, Guyana, and French Guiana (see Table S2 in supplementary material A), which have received less attention in terms of land-use change than their neighbouring countries (Hänggli et al. 2023). Land-use change processes in Venezuela also remain another knowledge gap. Although the Venezuelan Amazon did not seem to be undergoing extensive deforestation processes by the mid-2000s (Perz et al. 2005), this may now be challenged by infrastructure projects (Sy et al. 2015).

Here, we analysed the data across its entire geographical (i.e. all Amazonian countries), temporal (~ 1990–2020), and information (all drivers) extents. Nevertheless, users can subset drivers (different crops, livestock species, types of mining, etc.), specific subnational units or time periods according to their needs. Yet, at specific countries or periods of time, gaps and inconsistencies in the data—which we documented as extensively as possible—result more problematic. We recommend reviewing supplementary material A and supplementary file 1 to access information on data sources and specific characteristics of the data of interest.

We observed that a large number of dispersed datasets exist for most of the drivers we analysed, but these are difficult to collect and understand. In recent years, the initiative MapBiomas has produced several datasets of land cover data from remote sensing and compilations of spatial data that cover some of the same variables (and reveal similar trends) as in our work (MapBiomas 2024). Official efforts to further compile and systematize such data in other biomes and across an extended set of direct and indirect drivers would greatly benefit researchers and decision-makers. Open-source and transparent analyses of these data are also crucial if coherent progress is to be made on understanding and, ultimately, controlling agricultural frontier expansion and commoditization as key components of global change.

## Conclusion

Despite the globalized nature of frontiers commoditization, frontiers have evolved differently according to the socio-economic and political contextual conditions where they occur. The data we compiled and the analyses of land-use archetypes contribute to improving the understanding of changes in national and subnational land-use patterns in the Amazon by giving access to data that had not been previously compiled and analysed at this temporal and spatial scale. With these data, we show that cases of frontier commoditization that had formerly been observed at individual regions (e.g. Mackey (2011) or McCarthy (2020)) are part of a larger process of commoditization across the entire biome and that this process, which emerges from analyses of long-term statistical data, also reflects a diversity of contextual factors mediating frontier expansion and commoditization in the Amazon.

Furthermore, given the atypical land-use and crop expansion dynamics in agricultural frontiers (Eigenbrod et al. 2020), interventions need to be designed based on solid knowledge on the specific characteristics of each intervened frontier (Pendrill et al. 2022). While driver or value chain-specific interventions can reduce deforestation—e.g. the Brazilian Soy Moratorium—interventions need to be conceived for landscapes where multiple drivers interact with each other and with socio-economic dynamics to maximize their effectivity in the long term (Villoria et al. 2022). For instance, oil palm has expanded across different land-use archetypes (e.g. *Colombia_Meta*, *Brazil_northeast* or *Peru_amazonian*), although it interacts differently with other drivers and has expanded in different socio-economics contexts. Hence, although oil palm has not been a major deforestation driver in Latin America as in major producing countries (Furumo and Aide 2017), in the face of increasing demand (OECD and FAO 2021), interventions to control potential deforestation from palm need to address the land-use and socio-economic dynamics of the landscape where it is expanding. Initiatives like the jurisdictional approach recently piloted by RSPO in Ecuador, though focused on oil palm, aim to regulate its expansion while considering other land-use drivers and landscape characteristics (Ministerio de Agricultura y Ganadería et al. 2020). Similarly, some regions in Peru—such as Ucayali and San Martín—have implemented Regional Strategies for Low-Emission Rural Development (Gobierno Regional de San Martín 2021; Gobierno Regional de Ucayali 2021). Given the recent implementation of these interventions, their effects are still to be evaluated.

Lastly, we show that extractive industries have expanded largely across the Amazon in the last decades. At present, these industries have become major economic players and key sources of tax revenue in many developing countries, hence challenging deforestation control goals (Kinda and Thiombiano 2021). Although the forms governments spend the revenues from extractive industries can influence the deforestation impacts (Kinda and Thiombiano 2021), socio-economic challenges remain. Often, export-oriented primary industries contribute little to diversify and boost local economies in frontier regions (Barbier 2012). Hence, given many countries’ present and foreseeable dependence on natural resources’ extraction, land-use governance needs to be improved so that rents from these industries contribute to sustainable landscape management and inclusive social benefits.

The data and analyses of land-use archetypes presented here contribute building a knowledge base about the historic interplays between land-use changes, deforestation, and socio-economic and policy contexts. Such knowledge can inform future policy for controlling deforestation and for balancing social, economic, and environmental goals in the face of frontiers’ commoditization across much of the Global South.

## Supplementary Information

Below is the link to the electronic supplementary material.

Supplementary file1 (XLSX 33 KB)

Supplementary file2 (XLSX 73 KB)

Supplementary file3 (XLSX 19 KB)

Supplementary file4 (DOCX 18055 KB)

## Footnotes

1 https://osf.io/gvmwx/.

2 https://osf.io/gvmwx/.
