# Supplementary material for: Thirty years of drivers and patterns of land-use change across the Amazon biome
Source: Ambio. 2025 Jun 25;54(12):2135–53. doi: 10.1007/s13280-025-02199-5 (PMC12569279; doi:10.1007/s13280-025-02199-5)
Supplement: Supplementary file 4 — Supplementary file4 (DOCX 18055 KB) [file 13280_2025_2199_MOESM4_ESM.docx]

***Ambio***

Supplementary Information

*This supplementary information has not been peer reviewed.*

Title: **Thirty years of drivers and patterns of land use change across the Amazon biome**

This file includes:

**Supplementary material A.** Description of the data sources and data treatment, from selections of study units to descriptions of the data we found per driver for the different countries. This section contains two supplementary files: an Excel table describing every data we used (supp_file1_rawdata.xlsx) and a second excel file with the crop names’ equivalences per country, for the crop names’ harmonization process (supp_file2_crops_names.xlsx). Supplementary material A also contains table S1 (full list of study units) and table S2, which shows the total time span of the produced dataset for each country.

**Supplementary material B.** Plots of the yearly data series for the different drivers. Includes figures S1 to S57.

**Supplementary material C.** Biplots from principal components analysis (PCAs) and k-means clusters cross principal components one and two. These analyses were made for Brazil, Bolivia, Colombia, Ecuador, Peru, and for all of these combined (pan-amazonian). Includes figures S58 to S90.

**Supplementary material D.** Further details and plots of the rapid benchmarking of the collected dataset with the data produced by MapBiomas. Includes figures S91 to S93.

**Supplementary material E.** Plots of drivers and interventions aggregated by the archetypes present at 2020. Includes figures S94 to S100.

**Supplementary material F.** Plots showing the results from the Mann-Kendall analyses.

Other supplementary materials for this manuscript include the following:

**Supplementary file 1.** MOESM1.xlsx. Excel table describing (source, description, original format, temporal extent) every data we used.

**Supplementary file 2.** MOESM2.xlsx. Excel file with the crops names equivalences per country for the crops’ names harmonization process (supp_file2_crops_names.xlsx).

**Supplementary file 3.** MOESM3.xlsx. Excel file with the description of the columns of the processed dataset.

# SUPPLEMENTARY MATERIAL A

In this section we describe in detail the steps we took for processing drivers’ data. We also describe the data sources in more detail and give an overview of each country’s particular conditions such as: institutions in charge of the administration and governance of the drivers’ impacts on deforestation (e.g., environmental or agricultural ministries, protected areas services, etc.); institutions or services in charge of gathering official data and making it available to the public (mostly official statistics institutions); in the case of protected areas, institutional and governance structures in charge of managing the protected areas systems (governmental levels at which areas are managed, private and native communities participation, etc.).

All data used and all data-processing outputs (diagnosis plots, final time series matrices, etc.) are available at the Open Science Framework (OSF) repository at <https://osf.io/gvmwx/>

## Selection of study units

We selected the subnational units that overlapped the amazon regions of Amazonia – sensu stricto, Andes, and Guiana according to (Eva and Huber 2005). For Bolivia, Colombia, Ecuador, and Peru we used first level sub-national divisions: departments (*Departamentos*) for Bolivia, Colombia and Peru, provinces (*Provincias*) for Ecuador and states (*Estados*) for Venezuela. For Brazil, we used the groups of municipalities (*Municípios*) named Microregions (*Microrregião*). Given that the entire territories of Guyana, Suriname and French Guiana are contained in the study area, we used the entire countries as study units but also compiled subnational data where this were available, also at the first level divisions: Regions for Guyana, Districts for Suriname. In the case of Venezuela, we decided not to gather national level data when available because only about half of Venezuela is included in the Amazonian study area. See table SA.1 for the entire list of units included.

**Table S1. List of study units.** Complete list of study units for each country, coded name and area in km^2^. Coded name ’name_code’, corresponds to the standardised name we gave to each subnational unit, avoiding special characters and adding a country prefix. In the case of Brazil an additional term was added to the prefix, corresponding to the state where the microregion is located (AC: Acre, AM: Amazonas, AP: Amapá, MT: Mato Grosso, RO: Rondônia, RR: Roraima).

| **country** | **name** | **name_code** | **s_unit_km2** |
| --- | --- | --- | --- |
| Bolivia | Chuquisaca | BO_CHUQUISACA | 50943.24 |
| Bolivia | Cochabamba | BO_COCHABAMBA | 54449.75 |
| Bolivia | Santa Cruz | BO_SANTA CRUZ | 367729.86 |
| Bolivia | Pando | BO_PANDO | 63686.68 |
| Bolivia | Beni | BO_BENI | 206615.96 |
| Bolivia | La Paz | BO_LA PAZ | 128212.62 |
| Brazil | Porto Velho | BR_RO_PORTO VELHO | 65658.83 |
| Brazil | Guajará-Mirim | BR_RO_GUAJARA-MIRIM | 40803.84 |
| Brazil | Ariquemes | BR_RO_ARIQUEMES | 24333.50 |
| Brazil | Ji-Paraná | BR_RO_JI-PARANA | 25090.43 |
| Brazil | Alvorada D'Oeste | BR_RO_ALVORADA DOESTE | 15965.85 |
| Brazil | Cacoal | BR_RO_CACOAL | 24526.34 |
| Brazil | Vilhena | BR_RO_VILHENA | 26762.89 |
| Brazil | Colorado do Oeste | BR_RO_COLORADO DO OESTE | 14623.79 |
| Brazil | Cruzeiro do Sul | BR_AC_CRUZEIRO DO SUL | 31948.77 |
| Brazil | Tarauacá | BR_AC_TARAUACA | 53503.51 |
| Brazil | Sena Madureira | BR_AC_SENA MADUREIRA | 40546.01 |
| Brazil | Rio Branco | BR_AC_RIO BRANCO | 22264.59 |
| Brazil | Brasiléia | BR_AC_BRASILEIA | 15910.53 |
| Brazil | Rio Negro | BR_AM_RIO NEGRO | 332217.91 |
| Brazil | Japurá | BR_AM_JAPURA | 72657.91 |
| Brazil | Alto Solimões | BR_AM_ALTO SOLIMOES | 213235.26 |
| Brazil | Juruá | BR_AM_JURUA | 112647.59 |
| Brazil | Tefé | BR_AM_TEFE | 39890.38 |
| Brazil | Coari | BR_AM_COARI | 112082.39 |
| Brazil | Manaus | BR_AM_MANAUS | 41304.33 |
| Brazil | Rio Preto da Eva | BR_AM_RIO PRETO DA EVA | 31274.66 |
| Brazil | Itacoatiara | BR_AM_ITACOATIARA | 25414.94 |
| Brazil | Parintins | BR_AM_PARINTINS | 106944.88 |
| Brazil | Boca do Acre | BR_AM_BOCA DO ACRE | 63563.57 |
| Brazil | Purus | BR_AM_PURUS | 186851.64 |
| Brazil | Madeira | BR_AM_MADEIRA | 221083.06 |
| Brazil | Boa Vista | BR_RR_BOA VISTA | 67639.68 |
| Brazil | Nordeste de Roraima | BR_RR_NORDESTE DE RORAIMA | 30818.17 |
| Brazil | Caracaraí | BR_RR_CARACARAI | 73729.27 |
| Brazil | Sudeste de Roraima | BR_RR_SUDESTE DE RORAIMA | 51457.32 |
| Brazil | Óbidos | BR_PA_OBIDOS | 157598.58 |
| Brazil | Santarém | BR_PA_SANTAREM | 92473.48 |
| Brazil | Almeirim | BR_PA_ALMEIRIM | 90377.57 |
| Brazil | Portel | BR_PA_PORTEL | 45126.48 |
| Brazil | Furos de Breves | BR_PA_FUROS DE BREVES | 30067.99 |
| Brazil | Arari | BR_PA_ARARI | 27614.81 |
| Brazil | Belém | BR_PA_BELEM | 3129.56 |
| Brazil | Castanhal | BR_PA_CASTANHAL | 3751.86 |
| Brazil | Salgado | BR_PA_SALGADO | 5157.81 |
| Brazil | Bragantina | BR_PA_BRAGANTINA | 8755.11 |
| Brazil | Cametá | BR_PA_CAMETA | 16662.23 |
| Brazil | Tomé-Açu | BR_PA_TOME-ACU | 23714.89 |
| Brazil | Guamá | BR_PA_GUAMA | 28281.04 |
| Brazil | Itaituba | BR_PA_ITAITUBA | 189594.35 |
| Brazil | Altamira | BR_PA_ALTAMIRA | 226196.77 |
| Brazil | Tucuruí | BR_PA_TUCURUI | 32877.32 |
| Brazil | Paragominas | BR_PA_PARAGOMINAS | 48400.80 |
| Brazil | São Félix do Xingu | BR_PA_SAO FELIX DO XINGU | 121177.47 |
| Brazil | Parauapebas | BR_PA_PARAUAPEBAS | 22472.42 |
| Brazil | Marabá | BR_PA_MARABA | 20073.19 |
| Brazil | Redenção | BR_PA_REDENCAO | 21168.29 |
| Brazil | Conceição do Araguaia | BR_PA_CONCEICAO DO ARAGUAIA | 31195.42 |
| Brazil | Oiapoque | BR_AP_OIAPOQUE | 37151.50 |
| Brazil | Amapá | BR_AP_AMAPA | 20088.38 |
| Brazil | Macapá | BR_AP_MACAPA | 38643.67 |
| Brazil | Mazagão | BR_AP_MAZAGAO | 46586.44 |
| Brazil | Aripuanã | BR_MT_ARIPUANA | 124052.92 |
| Brazil | Alta Floresta | BR_MT_ALTA FLORESTA | 51376.07 |
| Brazil | Colíder | BR_MT_COLIDER | 42773.72 |
| Brazil | Parecis | BR_MT_PARECIS | 59584.21 |
| Brazil | Arinos | BR_MT_ARINOS | 54921.16 |
| Brazil | Alto Teles Pires | BR_MT_ALTO TELES PIRES | 54884.25 |
| Brazil | Sinop | BR_MT_SINOP | 50093.59 |
| Brazil | Paranatinga | BR_MT_PARANATINGA | 46959.48 |
| Brazil | Norte Araguaia | BR_MT_NORTE ARAGUAIA | 84934.66 |
| Brazil | Canarana | BR_MT_CANARANA | 60738.00 |
| Brazil | Médio Araguaia | BR_MT_MEDIO ARAGUAIA | 31657.49 |
| Brazil | Alto Guaporé | BR_MT_ALTO GUAPORE | 31355.71 |
| Brazil | Tangará da Serra | BR_MT_TANGARA DA SERRA | 22259.77 |
| Brazil | Jauru | BR_MT_JAURU | 18937.38 |
| Brazil | Alto Paraguai | BR_MT_ALTO PARAGUAI | 6673.60 |
| Brazil | Rosário Oeste | BR_MT_ROSARIO OESTE | 9200.01 |
| Brazil | Cuiabá | BR_MT_CUIABA | 24947.59 |
| Brazil | Alto Pantanal | BR_MT_ALTO PANTANAL | 53427.48 |
| Brazil | Primavera do Leste | BR_MT_PRIMAVERA DO LESTE | 11210.10 |
| Brazil | Tesouro | BR_MT_TESOURO | 26983.86 |
| Brazil | Rondonópolis | BR_MT_RONDONOPOLIS | 25542.40 |
| Brazil | Alto Araguaia | BR_MT_ALTO ARAGUAIA | 10692.33 |
| Colombia | Cauca | CO_CAUCA | 31071.69 |
| Colombia | Caquetá | CO_CAQUETA | 92795.47 |
| Colombia | Meta | CO_META | 82738.30 |
| Colombia | Nariño | CO_NARINO | 30858.93 |
| Colombia | Vaupés | CO_VAUPES | 53015.15 |
| Colombia | Putumayo | CO_PUTUMAYO | 25942.20 |
| Colombia | Guainía | CO_GUAINIA | 70656.91 |
| Colombia | Vichada | CO_VICHADA | 99370.93 |
| Colombia | Amazonas | CO_AMAZONAS | 109193.47 |
| Colombia | Guaviare | CO_GUAVIARE | 55499.36 |
| Ecuador | Azuay | EC_AZUAY | 8323.55 |
| Ecuador | Cañar | EC_CANAR | 3145.65 |
| Ecuador | Chimborazo | EC_CHIMBORAZO | 6495.42 |
| Ecuador | Cotopaxi | EC_COTOPAXI | 6105.37 |
| Ecuador | Tungurahua | EC_TUNGURAHUA | 3382.01 |
| Ecuador | Morona Santiago | EC_MORONA SANTIAGO | 23981.68 |
| Ecuador | Napo | EC_NAPO | 12513.31 |
| Ecuador | Orellana | EC_ORELLANA | 21548.61 |
| Ecuador | Pastaza | EC_PASTAZA | 29456.46 |
| Ecuador | Sucumbíos | EC_SUCUMBIOS | 18044.20 |
| Ecuador | Zamora Chinchipe | EC_ZAMORA CHINCHIPE | 10558.72 |
| France | Guyane française | FR_FRENCH GUIANA | 83313.34 |
| Guyana | Guyana | GU_GUYANA | 210839.25 |
| Peru | Amazonas | PE_AMAZONAS | 39266.80 |
| Peru | Apurímac | PE_APURIMAC | 21105.02 |
| Peru | Ayacucho | PE_AYACUCHO | 43530.69 |
| Peru | Cajamarca | PE_CAJAMARCA | 32833.96 |
| Peru | Cusco | PE_CUSCO | 71951.13 |
| Peru | Huancavelica | PE_HUANCAVELICA | 22079.05 |
| Peru | Huánuco | PE_HUANUCO | 37529.63 |
| Peru | [Junín](https://es.wikipedia.org/wiki/Departamento_de_Jun%C3%ADn) | PE_JUNIN | 44150.67 |
| Peru | La Libertad | PE_LA LIBERTAD | 25161.88 |
| Peru | Lambayeque | PE_LAMBAYEQUE | 14455.59 |
| Peru | Loreto | PE_LORETO | 374726.91 |
| Peru | Madre de Dios | PE_MADRE DE DIOS | 84520.51 |
| Peru | Pasco | PE_PASCO | 23894.77 |
| Peru | Piura | PE_PIURA | 35459.07 |
| Peru | Puno | PE_PUNO | 67445.85 |
| Peru | San Martín | PE_SAN MARTIN | 51005.48 |
| Peru | Ucayali | PE_UCAYALI | 104975.78 |
| Suriname | Suriname | SU_SURINAME | 144633.30 |
| Venezuela | Amazonas | VE_AMAZONAS | 179802.49 |
| Venezuela | Bolívar | VE_BOLIVAR | 250543.07 |
| Venezuela | Delta Amacuro | VE_DELTA AMACURO | 36870.91 |

## Data gathering and processing

Here we provide detailed descriptions of the data sources and data processing steps for the different at the Amazonian countries. A full list and description of all the individual datasets we compiled is available in the supplementary file 1 (supp_material1_datasets_table.xlsx).

### Population growth

We created population growth rate yearly time series from official government statistics. We used population estimates and census data, depending on each country’s data availability. When gap years existed in the published statistics, we interpolated the yearly series using all data points available. In all cases, we obtained a yearly series of total population and a series of yearly population growth rate.

#### Bolivia

Bolivia has produced official department level population estimates in different moments and for different time periods. We used the 2014 population revision for the period 2000-2011, and the 2020 revision for the period 2012-2020. These are available through the National Statistics Institute (Instituto Nacional de Estadística - INE) website at https://www.ine.gob.bo/index.php/censos-y-proyecciones-de-poblacion-sociales/ (Instituto Nacional de Estadística - INE 2022e), consulted on 11.17.2021. We then used the 1992 and 1976 census data to interpolate the entire 1990-2020 population series. Census data is available at <https://www.ine.gob.bo/index.php/censos-y-banco-de-datos/censos/> (Instituto Nacional de Estadística - INE 2022b), consulted on 11.17.2021.

#### Brazil

We obtained population estimates at the municipal level for Brazil for the periods 1989, 1992-1995, 1997-2006, 2008, 2009, 2011-2020 from the Brazilian Institute of Geography and Statistics (*Instituto Brasileiro de Geografia e Estatística*) at <https://www.ibge.gov.br/en/statistics/social/population/18448-estimates-of-resident-population-for-municipalities-and-federation-units.html?=&t=downloads> (Instituto Brasileiro de Geografia e Estatística 2021a), consulted on 9.29.2021

The estimates for the year 1989 are only available as a scanning of an old archive, so we manually captured the data from the selected municipalities.

Given that the configuration of the municipal borders has changed through time, for data aggregated at the municipal level, we manually identified the municipalities that were created or disappeared in the period 1990-2020 and their year of creation. We then integrated a yearly series where the population estimates of all the municipalities corresponded to their respective time period of existence.

Afterwards, we interpolated the series we produced to fill the gaps on the government estimates.

Lastly, we aggregated the municipal level data at the microregion level.

#### Colombia

Colombia has department level yearly population estimates for the entire temporal extent of our study, so we obtained these from the National Administrative Department of Statistics (*Departamento Administrativo Nacional de Estadística* – DANE) at https://www.dane.gov.co/index.php/estadisticas-por-tema/demografia-y-poblacion (Departamento Administrativo Nacional de Estadística – DANE 2022), consulted on 8.19.2021.

#### Ecuador

Ecuador also has yearly province population estimates for the entire period 1990-2020. These were generated by Ecuador’s National Institute of Statistics and Census (*Instituto Nacional de Estadística y Censos –* INEC) and we obtained them through the National System of Information (*Sistema Nacional de Información* - SNI) at <https://sni.gob.ec/proyecciones-y-estudios-demograficos> (Secretaría Nacional de Planificación 2021), consulted on 9.29.2021. The raw data consisted in three files: two retro-projections for the period 1990-2001 and 2001-2010 and one projection for the period 2010-2020.

#### Peru

We obtained census data for the years 1972, 1981, 1993 from the INEI National Series (*Series Nacionales*, *Instituto Nacional de Estadística e Informática -* INEI) service at <https://webapp.inei.gob.pe:8443/sirtod-series/> (Instituto Nacional de Estadística e Informática - INEI n.d.), consulted on 11.4.2021.

For the period 2000-2020 we obtained official yearly department level population estimates available from the Peruvian National Institute of Statistics and Informatics (*Instituto Nacional de Estadística e Informática -* INEI) (Instituto Nacional de Estadística e Informática - INEI 2022), consulted on 11.4.2021. We combined these two sources and used them to interpolate and complete the yearly series 1990-2020.

#### French Guiana

We obtained population projections for the French department of French Guiana (*Guyane française*) for the period 2000-2025 from the National Institute of Statistics and Economic Studies (*Institut national de la statistique et des études économiques*) at <https://www.insee.fr/en/statistiques/serie/001760178> (Institut national de la statistique et des études économiques 2023), consulted on 3.23.2023.

#### Guyana

For the period 1992 to 2000 only national level data for Guyana was available. We obtained these data from Guyana’s Bureau of Statistics, at <https://statisticsguyana.gov.gy/data/data-tables/> (Bureau of Statistics 2019), consulted on 9.29.2021.

We obtained subnational population projections for the period 2000-2020 from the United States Census Bureau at <https://www.census.gov/geographies/mapping-files/time-series/demo/international-programs/subnationalpopulation.html> (United States Census Bureau 2023), consulted on 2.27.2023.

#### Suriname

We compiled population data for Suriname from several sources. For the years 1980, 2004 and 2012 we obtained census population data at district level at <http://www.citypopulation.de/en/suriname/cities/> (City Population n.d.), consulted on 4.1.2023. This source states that data were obtained from: The Europa World Year Book 1991 (1980 data) and the 2004 and 2012 census data from the General Bureau of Statistics of Suriname (*Algemeen Bureau voor de Statistiek in Suriname*), although we could not locate this data from the original source.

We obtained districts’ population from 2015 at <https://www.city-facts.com/suriname/population> (city-facts 2023), consulted on 4.1.2023. For 2020 we used the GHSL - Global Human Settlement Layer from the European Commission's Joint Research Centre (European Commission. Joint Research Centre. 2023). This is a raster dataset of population estimates by pixel at 100 m resolution. We used it to obtain the total population for each district.

We combined these sources and used them to interpolate and complete the yearly series 1990-2020.

#### Venezuela

We obtained state level population census data of Venezuela from the literature (Siso Quintero 2012) for the years 1971, 1981, and 1990, as well as the official subnational population estimates for the period 2000-2050 published by the National Institute of Statistics of Venezuela (*Instituto Nacional de Estadística de Venezuela*) obtained at <http://www.ine.gov.ve/index.php?option=com_content&view=category&id=98&Itemid=51> (Instituto Nacional de Estadística de Venezuela 2011), consulted on 9.29.2021.

We used the census data and the yearly estimates for the period 2000-2020 to interpolate the 1990-2020 series.

### Agriculture

We put together subnational yearly time series of crops’ area from multiple sources for virtually every crop that was reported by the different countries. Agricultural data was the most challenging to process as several differences exist among countries and between different sources for the same country. For instance: some countries reported planted or harvested area or both, with different levels of completeness; some countries disaggregate the area dedicated to certain crops according to their use, like sugarcane for biodiesel or for sugar, whereas others do not or only star doing it at a certain point in time; some crops, at different points in time appear disaggregated by varieties and at other moments aggregated as a single crop; some of the data come from censuses whereas other come from surveys, etc.

We harmonised crops’ common names internally (i.e., standard names for each country in its original language -spanish or portuguese) and among countries (i.e., a single name in English per each crop across all countries) using the crops’ scientific names -or cultivars varieties when required- as a reference. Tables of names’ equivalences for harmonisation can be consulted in the supplementary file supp_file2_crops_names.xlsx.

For most countries we grouped the same crops when these were the different varieties of the same crop (e.g., different types of corn or beans) or when crops were disaggregated depending on their final use. These crops’ groupings can be changed by users in the agricultural data processing scripts by country, although the look-up tables that are called when harmonizing data in the scripts would need to be modified as well.

The final agricultural data tables contain planted and harvested areas reported, as well as the percentage of the study unit covered by the different crops.

#### Bolivia

We obtained yearly time series of Bolivian crops across the 1996-2020 time period from the National Statistics Institute (*Instituto Nacional de Estadística*) at https://www.ine.gob.bo/index.php/estadisticas-economicas/agropecuaria/agricultura-cuadros-estadisticos/ (Instituto Nacional de Estadística - INE 2022a), consulted on 7.18.2022. The variable reported was planted area. Bolivian agricultural statistics are compiled from several sources which include agricultural surveys, censuses and information obtained from companies and producers’ organizations (Instituto Nacional de Estadística - INE 2020).

Documentation is meant to be available at: <https://www.ine.gob.bo/index.php/estadisticas-economicas/agropecuaria/agricultura-metadatos/> but the access link seems to be broken (throughout 2023)

#### Brazil

We obtained yearly time series of Brazilian crops across the entire time period from the Brazilian repository of statistical tables (*Banco de Tabelas Estatíticas*). The data was separated between temporary crops, where area planted was reported, and permanent crops, where harvested area was reported. These data were obtained at <https://sidra.ibge.gov.br/tabela/1612> (Instituto Brasileiro de Geografia e Estatística 2021b), consulted on 11.1.202, and <https://sidra.ibge.gov.br/tabela/1613> (Instituto Brasileiro de Geografia e Estatística 2021c), consulted on 11.1.2021, respectively. This data originally come from a surveys undertaken at the municipal level (Instituto Brasileiro de Geografia e Estatística - IBGE 2014).

#### Colombia

We compiled department level agricultural statistics for the 1990-2020 period from the Information and Communication Network of the Colombian Agricultural and Livestock Sector (*Red de Información y Comunicación del Sector Agropecuario Colombiano -* Agronet). These were dispersed in different files for different time periods in the website of the Agricultural Evaluations - EVA and Statistical Yearbook of the Agricultural Sector at <https://www.agronet.gov.co/estadistica/Paginas/home.aspx?cod=59> (Ministerio de Agricultura y Desarrollo Rural 2018a), consulted on 3.3.2022. For the 1990-2005 period, we used the Statistical Yearbook of the Agricultural and Livestock Sector 2017 (*Historico EVA - desde el año 1987 al 2017*). For the 2006-2018 period we used the Municipal Agricultural Assessments 2007-2018 (*Base Agrícola EVA 2007-2018*). For 2019 and 2020 we used the 2019-2020 Municipal Agricultural Assessments - Agricultural Base 2019 – 2021 (*BaseEVA_Agrícola2019_2020_2021*). The variable reported was harvested area. No documentation on data provenance was found.

#### Ecuador

We compiled agricultural statistics from Ecuador’s National Institute of Statistics and Census (*Instituto Nacional de Estadística y Censos –* INEC). Only data for the period 2000-2020 were available. For the year 2000 we used data from the III National agricultural and livestock census (III Censo Nacional Agropecuario) available at <https://www.ecuadorencifras.gob.ec/censo-nacional-agropecuario/> (Instituto Nacional de Estadística y Censos 2022), consulted on 8.22.2022. The 2000 census had planted area data.

For the period 2002-2020 we used data from the Survey of Continuous Agricultural and Livestock Area and Production (*Encuesta de Superficie y Producción Agropecuaria Continua - ESPAC*). When we first did our search, these data were dispersed into different files in different sections of INEC’s webpage (see agriculture processing code) as follows:

2002-2014 at https://www.ecuadorencifras.gob.ec//encuesta-de-produccion-agropecuaria-continua/ https://www.ecuadorencifras.gob.ec//encuesta-de-produccion-agropecuaria-continua/ accessed 6.29.2022.

2015-2017: <https://www.ecuadorencifras.gob.ec/encuesta-de-superficie-y-produccion-agropecuaria-continua-2015-2016-2017-2/> accessed 7.29.2022.

2018: <https://www.ecuadorencifras.gob.ec/encuesta-de-superficie-y-produccion-agropecuaria-continua-2018/> accessed 7.3.2022.

2019: <https://www.ecuadorencifras.gob.ec/encuesta-de-superficie-y-produccion-agropecuaria-continua-2019/> accessed 7.3.2022.

2020: <https://www.ecuadorencifras.gob.ec/encuesta-de-superficie-y-produccion-agropecuaria-continua-2020/> accessed 7.3.2022.

At present all files can be accessed at <https://www.ecuadorencifras.gob.ec//encuesta-de-produccion-agropecuaria-continua/> (Instituto Nacional de Estadística y Censos n.d.)

No subnational agricultural data was found for the year 2001. Sufficient documentation exists both for the census (Instituto Nacional de Estadística y Censos and Ministerio de Agricultura y Ganadería 2002) and the ESPAC survey (Dirección de Estadísticas Agropecuarias y Ambientales 2023a; Dirección de Estadísticas Agropecuarias y Ambientales 2023b).

Large spikes in pastures’ area occur between 2011 and 2014 for which available documentation does not offer an explanation.

#### Peru

We compiled agricultural data for the period 1990-2020 from several official Peruvian sources.

For the period 1990-2011 we used the data compiled at the INEI National Series (*Series Nacionales*, *Instituto Nacional de Estadística e Informática -* INEI) service at https://webapp.inei.gob.pe:8443/sirtod-series/ (Instituto Nacional de Estadística e Informática - INEI n.d.), consulted on 11.4.2021.

For 2012 we used data from the IV National Agricultural Census (*IV Censo Nacional Agropecuario 2012*) available at <http://censos.inei.gob.pe/cenagro/tabulados/> (Instituto Nacional de Estadística e Informática - INEI n.d.), consulted on 11.22.2022. A noticeable spike in the area of crops planted in Peru can be observed in the year 2012, where the data comes from the national agricultural census rather than from estimates made by governmental agencies.

For the period 2016-2020 we used data from the Statistical yearbooks of agricultural production (*Anuario Estadístico de Producción Agrícola*) available at: <https://www.gob.pe/institucion/midagri/informes-publicaciones/2730325-compendio-anual-de-produccion-agricola> (Ministerio de Desarrollo Agrario y Riego 2021). These are yearly files.

In all cases, the information is a mix of planted and harvested area for different crops. No methodological documentation was found. No data was found for the period 2013-2015.

#### French Guiana

Agricultural data for French Guiana, for the entire French department, for the period 1990-2006 was obtained from the Statistics Division of the Food and Agriculture Organization of the United Nations (FAOSTAT) at <https://www.fao.org/faostat/en/#data/QCL> (Statistics Division of the Food and Agriculture Organization - United Nations (FAOSTAT) 2022), consulted on 3.13.2023.

#### Guyana

Agricultural data for Guyana for the period 1990-2020, at national level, was obtained from the Statistics Division of the Food and Agriculture Organization of the United Nations (FAOSTAT) at <https://www.fao.org/faostat/en/#data/QCL> (Statistics Division of the Food and Agriculture Organization - United Nations (FAOSTAT) 2022), consulted on 2.28.2023.

#### Suriname

Agricultural data for Suriname for the period 1990-2020, at national level, was obtained from the Statistics Division of the Food and Agriculture Organization of the United Nations (FAOSTAT) at <https://www.fao.org/faostat/en/#data/QCL> (Statistics Division of the Food and Agriculture Organization - United Nations (FAOSTAT) 2022), consulted on 3.6.2023.

#### Venezuela

No subnational agricultural data for Venezuela were found.

### Livestock

We created subnational yearly time series of livestock total population and population density (population/study unit area). We gathered data for major livestock species: cattle, pig, goat, sheep and buffalo (in Brazil and Colombia).

#### Bolivia

We obtained yearly series of total heard population of cattle, goat, pig and sheep at the departmental level from the Ministry of Rural Development and Land (*Ministerio de Desarrollo Rural y Tierras*) available from the Bolivian National Statistics Institute at <https://www.ine.gob.bo/index.php/estadisticas-economicas/ganaderia-y-avicultura/ganaderia-cuadros-estadisticos/> (Instituto Nacional de Estadística - INE 2022c), consulted on 9.16.2021.

Cattle data for the period 2017-2020 are estimates.

Documentation is meant to be available at: https://www.ine.gob.bo/index.php/estadisticas-economicas/ganaderia-y-avicultura/ganaderia-metadatos/ but the access link seems to be broken (throughout 2023).

#### Brazil

We obtained yearly series of total heard population of cattle, buffalo, goat, pig and sheep at the municipal level from the Municipal livestock inquiry (*Pesquisa da Pecuária Municipal*) available from the Brazilian Institute of Geography and Statistics at <https://sidra.ibge.gov.br/tabela/3939> (Instituto Brasileiro de Geografia e Estatística - IBGE n.d.), consulted on 11.2.2021.

#### Colombia

We obtained department level livestock population data from the Livestock Inventory available at the Information and Communication Network of the Colombian Agricultural and Livestock Sector (*Red de Información y Comunicación del Sector Agropecuario Colombiano -* Agronet) (Ministerio de Agricultura y Desarrollo Rural 2018a). We obtained data for cattle, goat, pig, sheep and buffalo (starting in 2004). We obtained sub-national data for the period 1995-2020. There were no data for the year 1998.

For the period 1995-2004 we used the Livestock Inventory compilation available at <https://www.agronet.gov.co/estadistica/Paginas/home.aspx?cod=65> (Ministerio de Agricultura y Desarrollo Rural 2018b), consulted on 10.4.2021. The present-day departments of Amazonas, Guainía, Guaviare, Vichada, Vaupés, made part of the *Territorios Nacionales* delimitiation until 1991, when they were declared departments. This former designation grouped a series of territories scarcely populated and economically underdeveloped. Probably because of this, these departments still appear grouped together in the period 1995 - 2003, then not mentioned for the period 1997-2001, and grouped as ‘other departments’ for the period 2002-2003 (this likely includes other departments as well, and is not possible to distinguish the proportion of the total of animals from different the different departments grouped). Hence for this time period and the mentioned departments, we only used data for the year 1995.

For the period 2005-2017 we used the Statistical Yearbook of the Agricultural and Livestock Sector 2017 (*Anuario Estadístico del Sector Agropecuario 2017*) available at <https://www.agronet.gov.co/estadistica/Paginas/home.aspx?cod=59> (Ministerio de Agricultura y Desarrollo Rural 2018a), consulted on 2.23.2022. We noticed inconsistencies in some of the departments and/or national totals for 2004, which is the year when these two datasets overlap.

For 2019 and 2020 we used the livestock inventories of those years available at <https://www.agronet.gov.co/estadistica/Paginas/home.aspx?cod=65> (Ministerio de Agricultura y Desarrollo Rural 2018b), consulted on 10.4.2021.

For livestock species other than cattle, the data sources we consulted do not have subnational level data for all departments. In different years, different departments are included in the inventory whereas the rest are grouped together as ‘other departments’; in some years, some species are included and others are not. For instance, goats are only listed in the departments within our study area in 2002.

Aside from the 1990-1995 gap, 2018 was the only year for which no livestock data was available.

#### Ecuador

We consulted the Ecuadorian National Agricultural and Livestock censuses and related surveys to obtain national and province level total population of cattle, goat, sheep and pig livestock. For the year 2000 we used the III National agricultural and livestock census (*III Censo Nacional Agropecuario*), available at <https://www.ecuadorencifras.gob.ec/censo-nacional-agropecuario/> (Instituto Nacional de Estadística y Censos 2022), consulted on 11.29.2021. For the period 2002-2020 we used the Survey of Continuous Agricultural and Livestock Area and Production (*Encuesta de Superficie y Producción Agropecuaria Continua*). As in agricultural data, livestock data was dispersed into subsections of INEC’s website but is at present all available at <https://www.ecuadorencifras.gob.ec//encuesta-de-produccion-agropecuaria-continua/> (Instituto Nacional de Estadística y Censos n.d.). We obtained sub-national data for the period 2000-2020. No subnational data were avalable for the period 1990-1999 and the year 2001. Adequate documentation exists both for the census (Instituto Nacional de Estadística y Censos and Ministerio de Agricultura y Ganadería 2002) and the ESPAC survey (Dirección de Estadísticas Agropecuarias y Ambientales 2023a; Dirección de Estadísticas Agropecuarias y Ambientales 2023b).

#### Peru

We integrated livestock population series for cattle, goat, pig and sheep from several official sources which had data for different time periods. No sub-national data is available prior to 1994, as informed by the Ministry of Agrarian Development and Irrigation (*Ministerio de Desarrollo Agrario y Riego*) when we requested it. We were, however, provided with national level data for the period 1960 -1995 under the Public Information Access mechanism (*Acceso a la Información Pública*) (CARTA Nro 1061-2021-MIDAGRI-SG/OACID-TRANSP). Nevertheless, we observed that this data is apparently highly underestimated when comparing it to the overlapping years available at the National Series service and to the national aggregates at the Statistical Yearbook of Livestock and Poultry Production. Hence, -and also given that no documentation explaining these inconsistencies existed- we decided to exclude this data.

We obtained department level yearly data for the period 1994-2010 from the INEI National Series (*Series Nacionales*, *Instituto Nacional de Estadística e Informática -* INEI) service at https://webapp.inei.gob.pe:8443/sirtod-series/ (Instituto Nacional de Estadística e Informática - INEI 2022), consulted on 11.5.2021.

For the period 2011-2020 we obtained data from the Statistical Yearbook of Livestock and Poultry Production 2020 (*Anuario Estadístico de la Producción Ganadera y Avícola 2020*) (Dirección General de Estadística, Seguimiento y Evaluación de Políticas 2020), consulted 3.2.2022. From this same source and time period we obtained yearly national data.

#### French Guiana

We obtained livestock data for cattle, goat, sheep and pig for the period 1990-2006 for the entire French department from the Statistics Division of the Food and Agriculture Organization of the United Nations (FAOSTAT) at <https://www.fao.org/faostat/en/#data/QCL> (Statistics Division of the Food and Agriculture Organization - United Nations (FAOSTAT) 2022), consulted on 3.13.2023.

#### Guyana

We obtained livestock data for cattle, goat, sheep and pig for Guyana for the period 1990-2020 at national level from the Statistics Division of the Food and Agriculture Organization of the United Nations (FAOSTAT) at <https://www.fao.org/faostat/en/#data/QCL> (Statistics Division of the Food and Agriculture Organization - United Nations (FAOSTAT) 2022), consulted on 2.28.2023.

#### Suriname

We obtained livestock data for cattle, goat, sheep and pig for Suriname for the period 1990-2020 at national level from the Statistics Division of the Food and Agriculture Organization of the United Nations (FAOSTAT) at <https://www.fao.org/faostat/en/#data/QCL> (Statistics Division of the Food and Agriculture Organization - United Nations (FAOSTAT) 2022), consulted on 3.6.2023.

#### Venezuela

No subnational livestock data were found for Venezuela.

### Mining

We compiled spatially explicit data about the extent of mining concessions granted at the different countries. Our processed output consists in the accumulated area at each year, based on the concessions’ area that was granted each year, as well as the corresponding percentage area covered by mining concessions per year.

The different countries had different degrees of information regarding the materials being mined and the type of concession. We aimed to re-define these categories into simpler ones to aid interpretation and comparison across countries. We re-categorized mining data into categories based on type of mining activity (industrial or artisanal), type of mineral (gold, metallic minerals, construction materials, etc.) or size of concession (large scale, medium scale or small scale) as follows:

Brazil: industrial and artisanal mining, Colombia: metallic mining, gold mining, construction materials mining and other mining, Ecuador: mining concessions, material banks and artisanal mining areas, Guyana: large scale concessions, medium scale concessions and small claims, Venezuela: gold mining, metallic mining and other mining; Bolivia Suriname and Peru did not allow recategorization and were processed as ‘all mining’. We did not find any data for French Guiana.

Data for Peru and Brazil came from the official mining cadasters. The rest of the countries did not have official information available but we found related datasets in public GIS repositories (ArcGIS online). Nevertheless, the provenance of these datasets is uncertain as no metadata or supporting information accompanied the information. In all cases we analysed only concessions that had already started activities (i.e., excluded mines with pending permits or not yet authorized by 2020) and that were granted after 1960.

#### Bolivia

We were unable to access data with temporal dimension on mining concessions granting for Bolivia and were only able to estimate the extent of concessions by 2017, based on the data compiled by the NGO RAISG (Amazon Geo-Referenced Socio-Environmental Information Network 2020), consulted on 9.8.2021.

#### Brazil

We obtained data on Brazilian mining concessions from the Brazilian Mining Geographic Information System (*Sistema de Informações Geográficas da Mineração - SIGMINE*) at <https://dados.gov.br/dados/conjuntos-dados/sistema-de-informacoes-geograficas-da-mineracao-sigmine> (Governo Federal and Agência Nacional de Mineração 2021), consulted on 4.3.2022. Brazilian mining data allows distinguishing between industrial (*lavra*) and artisanal (*garimpeira*) mining as well as between concessions for exploration, exploitation and those under request. We excluded concessions that were on request status and processed only exploration and exploitation concessions, reclassified into industrial and mining concessions.

#### Colombia

We obtained the mining cadaster polygons of Colombia from the NGO RAISG (Amazon Geo-Referenced Socio-Environmental Information Network 2020), consulted on 9.8.2021. These had no dates of concession granting, so we sourced these from the files consultation section of the website of the Colombian Mining Cadaster (*Catastro Minero Colombiano*) at <http://www.cmc.gov.co/CmcFrontEnd/consulta/index.cmc> (Agencia Nacional de Minería - ANM n.d.), consulted on 11.30.2022. We then incorporated the dates onto the concessions’ polygons.

The mining cadaster from Colombia allows properly differentiating different minerals being mined, but to make data as homogeneous as possible with respect to the rest of the countries, we reclassified it into metallic mining, gold mining, construction materials mining and other mining.

#### Ecuador

We obtained the mining cadaster polygons of Ecuador from the ArcGIS online repository at <https://www.arcgis.com/home/item.html?id=e7d53bb464f6488aa9981e552505f47e> (ESRI 2021a), consulted on 11.11.2021. This is the same data that is available for consultation at <https://arcmineria.maps.arcgis.com/apps/webappviewer/index.html?id=27bfda03ce4342b3834a27010da857e5>

Ecuadorian mining data allows differentiating into mining concessions, material banks and artisanal mining areas.

#### Peru

We obtained the mining cadaster polygons of Peru from the GEOCATMIN service of the Geological, Mining and Metallurgical Institute of Peru (*Instituto Geológico Minero y Metalúrgico -INGEMMET*) available at <https://geocatmin.ingemmet.gob.pe/geocatmin/> (Instituto Geológico Minero y Metalúrgico -INGEMMET n.d.), consulted on 5.17.2022. Peruvian mining cadaster does not allow distinguishing different materials mined so we processed it as ‘all mining’.

#### Guyana

Spatial data of mining concessions from Guyana were obtained from the ArcGIS online repository. Data for three different types of concessions, based on size, were obtained.

Large scale concessions data were obtained at <https://www.arcgis.com/home/item.html?id=6b998419c87c439d80dbbb82f159f7b0> (ESRI 2021b), consulted on 10.4.2022.

Medium scale concessions data were obtained at <https://www.arcgis.com/home/item.html?id=952d6c39b6574a029cbfcf33684740aa> (ESRI 2021d), consulted on 2.27.2023.

Small scale mining claims data were obtained at <https://www.arcgis.com/home/item.html?id=0f0898a677f8419bac2e2da6c5a7f91b> (ESRI 2021g), consulted on 2.27.2023.

#### Suriname

Spatial data of mining concessions from Suriname were obtained from the ArcGIS online repository at

<https://www.arcgis.com/home/item.html?id=70cc7a4e2e45442a989a963eb5c7eaf8> (ESRI 2021h). This data does not allow differentiating between types of mining or minerals, so it was processed as ‘all mining’.

#### Venezuela

We were unable to access data with temporal dimension on mining concessions granting for Venezuela and were only able to estimate the extent of concessions by 2020, based on the data compiled by the NGO RAISG (Amazon Geo-Referenced Socio-Environmental Information Network 2020), consulted on 9.8.2021. We recategorized the data into gold mining, metallic mining and other mining

### Oil

We compiled spatially explicit data about the extent of oil blocks at the different countries. Our processed output consists in the accumulated area at each year, based on the blocks’ area that were contracted each year, as well as the corresponding percentage covered by oil blocks per year.

We obtained data on oil blocks from a diversity of sources. Brazil was the only country for which these data could be consulted directly from an official source. The rest of the countries that we found data for, we obtained this from secondary sources. As with mining concessions, we kept for analyses those blocks that had been already assigned for exploration or exploitation throughout the study period.

#### Bolivia

We obtained polygons of Bolivian oil blocks from the NGO RAISG at [https://www.amazoniasocioambiental.org/en/maps/#!/areas](https://dev.amazoniasocioambiental.org/en/maps/) (Amazon Geo-Referenced Socio-Environmental Information Network 2020), consulted on 9.8.2021, and the dates of blocks contracting from the Hydrocarbons Geographic Information System of the Jubileo Foundation at http://200.105.166.222/WebSiteV30/Descarga_pdf.aspx and <http://200.105.166.222/WebSiteV30/> (Fundación Jubileo n.d.), consulted on 4.17.2023. We then manually incorporated the date of contracting to the spatial polygons. We also observed that a few oil blocks’ names had been captured wrongly in the RAISG polygons, so we manually corrected these names (see oil_bolivia processing code).

We kept only blocks that were at exploration or exploitation phase, i.e., did not considered lot currently being offered by the government but at which no company (either private or state-owned) had started working. The earliest contracted block is from 2006.

#### Brazil

Brazil is the only country for which data about oil blocks is available at official repositories. Up to date information on the status of Brazilian oil blocks as well as spatial data can be accessed at: <https://www.gov.br/anp/pt-br/assuntos/exploracao-e-producao-de-oleo-e-gas/dados-tecnicos/shapefile-de-dados> (Ministério de Minas e Energia n.d.), consulted on 3.13.2023.

Nevertheless, to match the temporal extent of the rest of the data, we processed Brazilian oil blocks according to their status by 2020. This means we processed the blocks that were being explored by that time, but for which (after 2020) no exploitation contracts were finally given. We obtained such polygons from the NGO RAISG at <https://www.amazoniasocioambiental.org/en/maps/> (Amazon Geo-Referenced Socio-Environmental Information Network 2020), consulted on 9.8.2021. These data correspond to the same polygons available at the above-mentioned website under the label ‘*Blocos das rodadas concluídas*’, but in the RAISG sources these have already been pre-selected for the amazonian biome, whereas in the direct Brazilian source, one has to sort them by yearly offers rounds.

We then obtained the dates of exploration contract granting for these bocks at: <https://cpl.anp.gov.br/anp-cpl-web/public/sigep/consulta-blocos-exploratorios-fase-encerrada/consulta.xhtml?dswid=8074> (Agência Nacional do Petróleo, Gás Natural e Biocombustíveis - ANP 2023) and manually incorporated it into the polygons.

We classified Brazilian blocks into exploration and exploitation according to their status by 2020 and compiled the yearly series for the period 1998-2020.

#### Ecuador

We obtained polygons of oil blocks at Ecuador from the NGO RAISG at <https://www.amazoniasocioambiental.org/en/maps/> (Amazon Geo-Referenced Socio-Environmental Information Network 2020). consulted on 9.8.2021. We could not find the date of establishment of Ecuadorian oil blocks from secondary sources so we only analysed the extent of blocks existing by 2017.

#### Colombia

We obtained oil blocks polygons from Colombia from the ArcGIS online repository at <https://www.arcgis.com/home/item.html?id=58077b8c29ac43d088c90f1ae4993a06> (ESRI 2021e), consulted on 4.13.2023. These data are the same that is available for consultation at the official geo-viewer from the National Hydrocarbon Agency (*Agencia Nacional de Hidrocarburos*) at <https://geovisor.anh.gov.co/tierras/> but for which the downloading link is broken. Earliest block was contracted in 2001.

#### Peru

We obtained oil blocks polygons from Peru from the ArcGIS repository at <https://www.arcgis.com/home/item.html?id=3a0bf913b34c4e1ea4c08a4f3fdd02bf> (ESRI 2021c), consulted on 4.13.2023. We selected for analyses the lots that had already being assigned for exploration or exploitation or that were under technical evaluation but had already been assigned to a company. These categories are: Contract Lots (*Lotes de Contrato*), Technical Evaluation Agreement, (Convenio de Evaluación Técnica) and plots in Negotiation (*Lotes en Negociación*). We sourced the dates of establishment of the five blocks under Negotiation from secondary sources (see processing code). Earliest block was contracted in 1991.

#### French Guiana

We did not find any information on extent of oil blocks in French Guiana.

#### Guyana

We did not find any information on extent of oil blocks in Guyana.

#### Suriname

We did not find any information on extent of oil blocks in Suriname.

#### Venezuela

We obtained polygons of oil blocks at Venezuela from the NGO RAISG at <https://www.amazoniasocioambiental.org/en/maps/> (Amazon Geo-Referenced Socio-Environmental Information Network 2020). consulted on 9.8.2021. We could not find the date of establishment of Venezuelan oil blocks from secondary sources so we only analysed the extent of blocks existing by 2019.

### Roads

We only found data on the temporal evolution of roads for Brazil and Bolivia. Our processed outputs consisted on the yearly extension in km of roads per study unit as well as roads’ density per study unit (roads extension/ study unit area in km2).

#### Bolivia

We obtained yearly tabulated data of roads’ extension for the period 2000-2020 from the website of the National Statistics Institute (Instituto Nacional de Estadística - INE) at <https://www.ine.gob.bo/index.php/estadisticas-economicas/transportes/longitud-de-caminos-cuadros-estadisticos/> (Instituto Nacional de Estadística - INE 2022d). According to the data, the information was provided by the Bolivian main roads authority (Administradora Boliviana de Carreteras), the departments’ roads’ service offices (Servicios Departamentales de Caminos) and the Autonomous Municipal Governments (Gobiernos Autónomos Municipales).

#### Brazil

Brazil has shapefiles of main roads evolution aggregated by decades. These span from the 1960s to the 2010s. We obtained these data from the Brazilian Infrastructure Ministry (Ministério da Infraestrutura) at <https://www.gov.br/infraestrutura/pt-br/assuntos/dados-de-transportes/bit/bitmodosmapas> (Ministério da Infraestrutura 2021), consulted on 4.24.2022. However, the URL for accessing the data became unavailable at some point in early 2023 and we were unable to locate the data elsewhere.

We processed the shapefiles to obtain tabulated yearly data of the extension and density of roads per microregion for the period 1990-2020.

### Protected areas

We integrated a spatial dataset of the Protected areas (PAs) at the different governmental levels stablished by each country. We obtained the datasets from governmental sources when these were available. When this was not the case, we obtained the spatial data from several sources (See supplementary file 1), and verified the accuracy of the information as best as possible according to the information available for each country.

As a general reference, we used the national Protected Areas shapefile compiled by the NGO RAISG (Amazon Geo-Referenced Socio-Environmental Information Network 2020) as well as the Protected Planet World Database on Protected Areas (WDPA) global dataset (UNEP-WCMC and IUCN 2022). When no official shapefiles existed, either directly from governmental sources or stored in other repositories, we used the WDPA dataset.

Since not all countries have homogenized their protected areas categorization under, for instance, IUCN criteria, we implemented our own categorization for all the protected areas in our study area. This was based on the governmental administrative level (national, subnational or private) and level of use (direct or indirect). Hence, we had the following categories national-direct, national-indirect, regional-direct, regional-indirect, and private.

Where these existed, we also included Biosphere Reserves and Ramsar sites.

#### Bolivia

Bolivia has a National Protected Areas System (Sistema Nacional de Áreas Protegidas - SNAP) with areas protected at the national, regional (departamental) and municipal (municipal) level. National protected areas are managed by the National Service of Protected Areas (Servicio Nacional de Áreas Protegidas - SERNAP) (Servicio Nacional de Áreas Protegidas 2020). One national protected area has been declared as national park and indigenous territory (Parque Nacional y Territorio Indigena Isiboro Securé). Historically, regional and municipal areas have been created for several regional and local objectives, under numerous categories and managed by several institutions. This has driven low integration, among the different governmental levels and difficulties in identifying the group of biodiversity values that subnational level protected areas are protecting (Dirección Gral. de Biodiversidad y Areas Protegidas and Servicio Nacional de Areas Protegidas 2012).

The figure of private protected areas (Reservas Privadas del Patrimonio Natural) exists in the Bolivian law but no information about any private area declared was found by us or by (Dirección Gral. de Biodiversidad y Areas Protegidas and Servicio Nacional de Areas Protegidas 2012).

Areas of Immobilization, which have been proposed as protected areas but are awaiting studies to define its final categorization and zonification are also considered in the SERNAP (Dirección Gral. de Biodiversidad y Areas Protegidas and Servicio Nacional de Areas Protegidas 2012).

We obtained the protected areas shapefile of Bolivia from the WDPA (UNEP-WCMC and IUCN 2022). Given the multitude of categories and lack of official information regarding the conservation objectives, management plans or any other information about the use level exercised in the different categories, we used the homogenization categories proposed by (Dirección Gral. de Biodiversidad y Areas Protegidas and Servicio Nacional de Areas Protegidas 2012), and any other sources available (sub-national governments websites, protected areas websites, etc.) to determine the level of use.

We obtained Legal act information from (Dirección Gral. de Biodiversidad y Areas Protegidas and Servicio Nacional de Areas Protegidas 2012) and (Servicio Nacional de Áreas Protegidas (SERNAP) 2012) and wrote it into the shapefile.

#### Brazil

Brazilian Protected Areas are named Conservation Units (Unidades de Conservação) and are administered at the three governmental levels, national (federais - federal), regional (estadual - state) and local (municipal - municipal); private protected areas exist as well. National protected areas are administered by the Chico Mendes Institute of Biodiversity Conservation (Instituto Chico Mendes de Conservação da Biodiversidade) (Instituto Chico Mendes de Conservação da Biodiversidade 2020b), whereas state and municipal areas are administered by the environmental offices of the respective governments (Presidência da República 2000).

Brazilian private protected areas are named Private Reserves of Natural Patrimony (Reservas Particulares do Patrimônio Natural - RPPN) and can as well be established at the national, state or municipal level (de Souza et al. 2012).

The official National Protected Areas shapefile (Instituto Chico Mendes de Conservação da Biodiversidade 2020a) had all the information we were gathering, so no complementary information was added. We included two national protected areas from the RAISG shapefile (Estação Ecológica Iquê and Reserva Ecológica Sauim-Castanheira).

We sourced state, municipal and some private protected areas from a shapefile of all Brazilian conservation units compiled by the Coordination of Situation and Information Management of the Brazilian National Water Agency – ANA (Agência Nacional de Águas - Coordenação de Conjuntura e Gestão da Informação and Ministério do Meio Ambiente 2019). No complementary additions were made to this dataset either.

We used the official shapefile of all Brazilian private protected areas compiled by the Chico Mendes Institute of Biodiversity Conservation (Instituto Chico Mendes de Conservação da Biodiversidade n.d.), which did not have the date nor the legal act when the area was established. This information can be consulted through the RPPN Automated Monitoring System of the Chico Mendes Institute, so we manually searched for the legal act of each area and wrote it into the shapefile. The most recently declared RPPN found in this source is from 2019 (RPPN Frigonosso). We added four additional RPPNs not included in this shapefile, but that were included in the shapefile compiled by ANA and for which we manually verified their existence and localization. These are: RPPN Cristalino I, RPPN Cristalino III, RPPN Fazenda Loanda and RPPN Peugeot-ONF-Brasil.

#### Colombia

Colombia has national, regional and local, privately administered protected areas. All areas form the National System of Protected Areas (Sistema Nacional de Áreas Protegidas – SINAP) and are registered into the Unique National Register of Protected Areas (Registro Único Nacional de Áreas Protegidas - RUNAP). National protected areas are administered by National Natural Parks of Colombia (Parques Nacionales Naturales de Colombia), which depends on the Ministry of the Environment. National Parks of Colombia is also in charge of the register and evaluation of land plot that request to be declared as private protected areas (Parques Nacionales Naturales de Colombia 2021a).

We obtained the official RUNAP shapefile (Parques Nacionales Naturales de Colombia 2021b) but noticed that it did not have the date of the legal act of most of the Protected Areas (It had, however, the dates of most of the areas declared after 2020). We found and older version of this shapefile on the ArcGIS Online repository at https://www.arcgis.com/home/item.html?id=4a673b3d51c146abab5fd462992dd2c0 (ESRI 2021f), consulted on 1.28.2022, which did have the dates of the legal act of all areas prior to 2020, so we merged these two to have a single, updated and complete shapefile.

#### Ecuador

In Ecuador, the National System of Protected Areas (Sistema Nacional de Áreas Protegidas del Ecuador - SNAP) is formed by a national, regional, local and private subsystems. National protected areas integrate the Patrimony of Natural Areas of the State (Patrimonio de Áreas Naturales del Estado - PANE) subsystem and are administered by the Ministry of the Environment. Regional protected areas integrate the Decentralized Autonomus subsystem (Áreas Protegidas de los Gobiernos Autónomos Descentralizados - APG) and are administered by subnational autonomous governments at the Provincia and Canton level. (Ministerio del Ambiente 2015; Ministerio del Ambiente del Ecuador 2016). The Community Protected Areas subsystem (Áreas Protegidas Comunitarias - APC) was recently established (first APC, Área Protegida Comunitaria Tambillo, was declared in 2018) to allow local indigenous and afroecuatorian communities to propose and administer protected areas (Ministerio del Ambiente 2015; Ministerio del Ambiente, Agua y Transición Ecológica 2018).

The Private Protected Areas subsystem (Subsistema de Áreas protegidas privadas - APPRI) was recently established as well. The firs private protected area, Área Protegida Privada Bellavista, was declared in 2019 but is outside of our study area (Ministerio del Ambiente 2015). An additional private area has been declared (Ministerio del Ambiente, Agua y Transición Ecológica 2020) but no spatial information exists yet.

Ecuador also has a protection category named Protection Forests (Bosques Protectores). These consist on vegetated areas that, given their rough topography, are not suitable for agriculture or livestock grazing, and are instead protected (Sistema Nacional de Áreas Protegidas del Ecuador and Ministerio del Ambiente 2015).

We obtained the SNAP shapefile from the ArcGIS online repository at <https://www.arcgis.com/home/item.html?id=e7517da8645e4d19bc9bf2ce02f3ab4b> (ESRI 2022) and the shapefile of Ecuadorian Protection Forests from the NGO RAISG at <https://www.amazoniasocioambiental.org/en/maps/> (Amazon Geo-Referenced Socio-Environmental Information Network 2020), consulted on 9.8.2021.

#### Peru

Peru has national, regional and private Protected Areas systems. National protected areas are administered by the National Service of Natural Protected Areas (Servicio Nacional de Áreas Naturales Protegidas por el Estado - SERNANP); Regional Conservation Areas (Áreas de Conservación Regional – ACR) are proposed and administered by regional governments. Private areas are proposed by the owners of the land and approved by the SERNANP and the Ministry of the Environment (Ordóñez 2018). Altogether they form the National System of Natural Areas Protected by the State (Sistema Nacional de Áreas Naturales Protegidas por el Estado - SINANPE).

Peruvian protected areas’ legislation contemplates buffer zones (zonas de amortiguamiento) as well as reserved zones (zonas reservadas). Buffer zones surround national protected areas only, and the specific zonation and activities allowed inside them are defined in the areas’ Master Plans. Reserved zones are areas reserved that have the characteristics needed to become national protected areas but complementary studies are yet to be completed before these can be formally declared protected areas. Despite not being declared, they make part of the SINANPE (Ordóñez 2018; Estado Peruano 2022b).

Peru has communally administered reserves (Reserva Comunal) which are in charge of local rural communities (Estado Peruano 2022a).

Official shapefiles of all the protected areas and special zones (buffer and reserved zones) are available through the spatial data portal of the SERNANP at <https://geo.sernanp.gob.pe/visorsernanp/> (Servicio Nacional de Áreas Naturales Protegidas por el Estado - SERNANP 2022). These contain all the information we were gathering, so no additions were made.

#### Venezuela

Venezuela put in force the Organic Law for Territory Planning (Ley Orgánica para la Ordenación del Territorio – LOPOT, put in force in 1983) which defines several land use categories termed Areas under Special Administration Regime, all under national administration (Áreas Bajo Régimen de Administración Especial - ABRAE) (García Peña and Silva Viera 2013). These land use categories have been portrayed by the national authorities as protected areas, although some of them include uses not compatible with biodiversity conservation (García Peña and Silva Viera 2013; García Peña et al. 2019).

We obtained Venezuelan protected areas polygons from the WDPA (UNEP-WCMC and IUCN 2022) and reclassified their use categories based on the IUCN categories the WDPA data already has.

#### Guyana

Guyana stablished its National Protected Areas System (NPAS) in 2011, when the Protected Areas Act (2011) was put in order (Protected Areas Trust (Guyana) 2022c). A total of five protected areas exist in Guyana. Protected areas are managed at the national level by the Protected Areas Commission (PAC), excepting the Iwokrama International Centre for Rainforest Conservation and Development, which preexisted the creation of the NPAS and is managed as an international non-for-profit organization (Iwokrama International Centre for Rainforest Conservation and Development 2020; Protected Areas Trust (Guyana) 2022b). All protected areas in Guyana include sustainable use of natural resources.

We obtained Guyana’s protected areas shapefiles from the WDPA (UNEP-WCMC and IUCN 2022). WDPA includes an equivalence with IUCN categorization. For Guyana, all protected areas are listed as category VI: Protected area with sustainable use of natural resources (UNEP-WCMC and IUCN 2022).

Guyana has one large area owned and managed by indigenous people, Kanashen Amerindian Protected Area (Protected Areas Trust (Guyana) 2022a).

#### Suriname

Suriname has a fragmented protected areas system. The Nature Conservation Division (NCD) of the Ministry of Natural Resources is in charge of managing the protected areas system, whereas the Foundation for Nature Conservation in Suriname (Stichting Natuurbehoud Suriname - STINASU) is in charge of some indirect uses of some reserves (eco-tourism, research) and effectively implementing management and surveillance (Ouboter 2002; Delvoye et al. 2017).

We obtained the protected areas shapefile from the WDPA (UNEP-WCMC and IUCN 2022) (Table #). Areas where status was listed as ‘proposed’ were discarded. This was verified on (Ouboter 2002) and (Vereniging van Inheemse Dorpshoofden in Suriname - VIDS 2009).

### Indigenous Territories

We used the Indigenous Territories spatial dataset compiled by the NGO RAISG at <https://www.amazoniasocioambiental.org/en/maps/> (Amazon Geo-Referenced Socio-Environmental Information Network 2020), consulted on 9.8.2021. Data for Ecuador and Guyana had no dates of territories’ legal recognition. Suriname has not granted legal recognition to indigenous territories (International Work Group for Indigenous Affairs 2021). The data compiled by RAISG does contain information on different aspects of the indigenous peoples that inhabit those territories. Nevertheless, for the purpose of this work, we did not consider any subcategories for indigenous territories and simply grouped them as ‘all indigenous territories’. Most of the data from RAISG was updated in 2017.

#### Bolivia

The data includes territories that have been demanded (Demandada) and titled (Titulada). We only analysed territories that had been titled.

#### Brazil

Brazilian data includes territories in different stages of the legal recognition and official demarcation process. We selected for further analysis those territories that have already finished the demarcation process and already have a Declaratory Ordinance, as well as those demarcated areas where isolated indigenous groups are granted protection. These corresponds to the categories: Approved (Homologada), Registered (Registrada), Declared (Declarada), Reserved (Reservada) and with Restriction of Use (com restrição de uso).

#### Colombia

All Colombian indigenous territories had the status of Created (Creado) and we analysed all of these.

#### Ecuador

In Ecuador we selected those territories with the status of Agreement (Convenio), Decreed (Decretada) and Titled (Titulada). Ecuadorian data did not have dates of legal declarations so we only analysed the extent of indigenous territories by 2017.

#### Peru

The Peruvian data from RAISG contains polygons of Indigenous Territories (Comunidad nativa) and also of pedestrian communities (Comunidad campesina) but none of the later had dates of recognition and we could not find them on secondary sources. Hence, we only analysed Indigenous territories through time. We kept only territories that had been titled or decreed.

#### French Guiana

French Guiana declared a series of Indigenous Territories in the mid-1990s but since then there has not been additional territories declared.

#### Guyana

Indigenous Territories data from Guyana had no dates of territories’ recognition and we could not locate these elsewhere.

#### Suriname

No legally recognized Indigenous Territories exist in Suriname (International Work Group for Indigenous Affairs 2021).

#### Venezuela

Data from Venezuela contains territories legally recognized after 2005, after the *Ley Orgánica de Pueblos y Comunidades Indígenas*.

**Table S2.** **Total time span of the produced dataset for each country.** Years in italics in Guyana, Suriname and French Guiana indicate data which were only available at national level. Data of roads evolution in Brazil are available as compiled shapefiles per decade, so changes are only registered at the turn of each decade.

| **COUNTRY** | 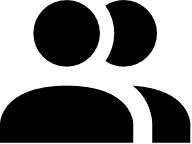  POPULATION | 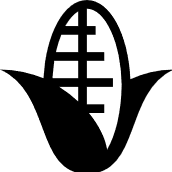  CROPS | 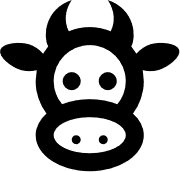  LIVESTOCK | 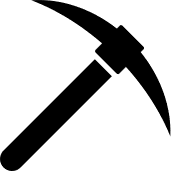  MINING | 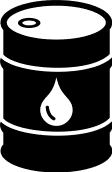  OIL | 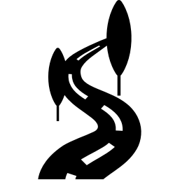  ROADS | 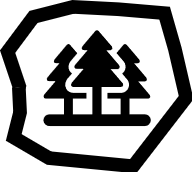  PROTECTED AREAS | 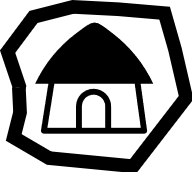  INDIGENOUS TERRITORIES |
| --- | --- | --- | --- | --- | --- | --- | --- | --- |
| **Brazil** | 1990-2020 | 1990-2020 | 1990-2020 | 1990-2020 | 1998-2020 | 1990-2020 | 1990s-2020 | 1990-2020 |
| **Bolivia** | 1990-2020 | 1996-2020 | 1990-2020 | 2020 | 2006-2020 | 2000-2020 | 1990-2020 | 1990-2020 |
| **Colombia** | 1990-2020 | 1990-2020 | 1995-2020 | 1990-2020 | 2001-2020 | - | 1990-2020 | 1990-2020 |
| **Ecuador** | 2000-2020 | 2000-2020 | 2000-2020 | 1991-2020 | 2017 | - | 2000-2020 | 2020 |
| **Peru** | 1990-2020 | 1990-2020 | 1994-2020 | 1990-2020 | 1991-2020 | - | 1990-2020 | 1990-2020 |
| **Guyana** | 1992-2020 | *1990-2020* | *1990-2020* | 1990-2020 | - | - | 1990-2020 | 2009 |
| **Suriname** | 1990-2020 | *1990-2020* | *1990-2020* | 1996-2020 | - | - | 1990-2020 | - |
| **French Guiana** | *1990-2020* | *1990-2006* | *1990-2006* | *-* | *-* | *-* | *1990-2020* | *1991-2020* |
| **Venezuela** | 1990-2020 | - | - | 2020 | - | - | 1990-2020 | 2005-2020 |

# SUPPLEMENTARY MATERIAL B

In this section we plot the time series of processed data of the different drivers. Population data is plotted at subnational level and both total population and yearly percentage change are plotted. French Guiana was the only country for which subnational population data could not be obtained so plot shows the data processed for the entire French department.

Agricultural data is plotted as total area in hectares, at national level, aggregated by crop, using the harmonized unique names we defined for each crop. For countries that had both planted and harvested data, we provide both plots. Livestock data is plotted as total livestock population at national level aggregated by livestock species.

Mining and oil data are plotted both at national level, aggregated by the categories we defined for each country based on data characteristics (see Mining and Oil sections in Supplementary Material A), and also the total extension aggregated by subnational unit, when possible. At national level we plotted total mining concessions and oil blocks extension in km^2^, whereas subnational plots represented the proportional extension by subnational unit.

For roads, we plotted the total extension in km at national level, aggregated by type of road, and total roads area by subnational units summed across all types of roads (only evolution of main roads was available for Brazil).

For Protected Areas and Indigenous Territories, we also plotted total extension by categories (see Protected Areas and Indigenous Territories sections in Supplementary Text A) at national level and proportional extension (total across all categories) at subnational units.

For French Guiana and Venezuela, we plot all data at national level only, excepting population for Venezuela which we plotted at subnational level.

### Population growth

| 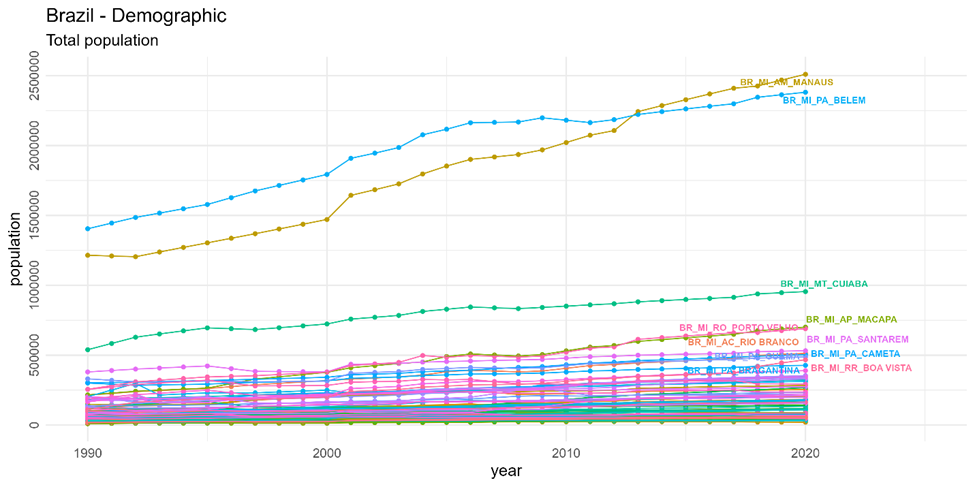 |
| --- |
| 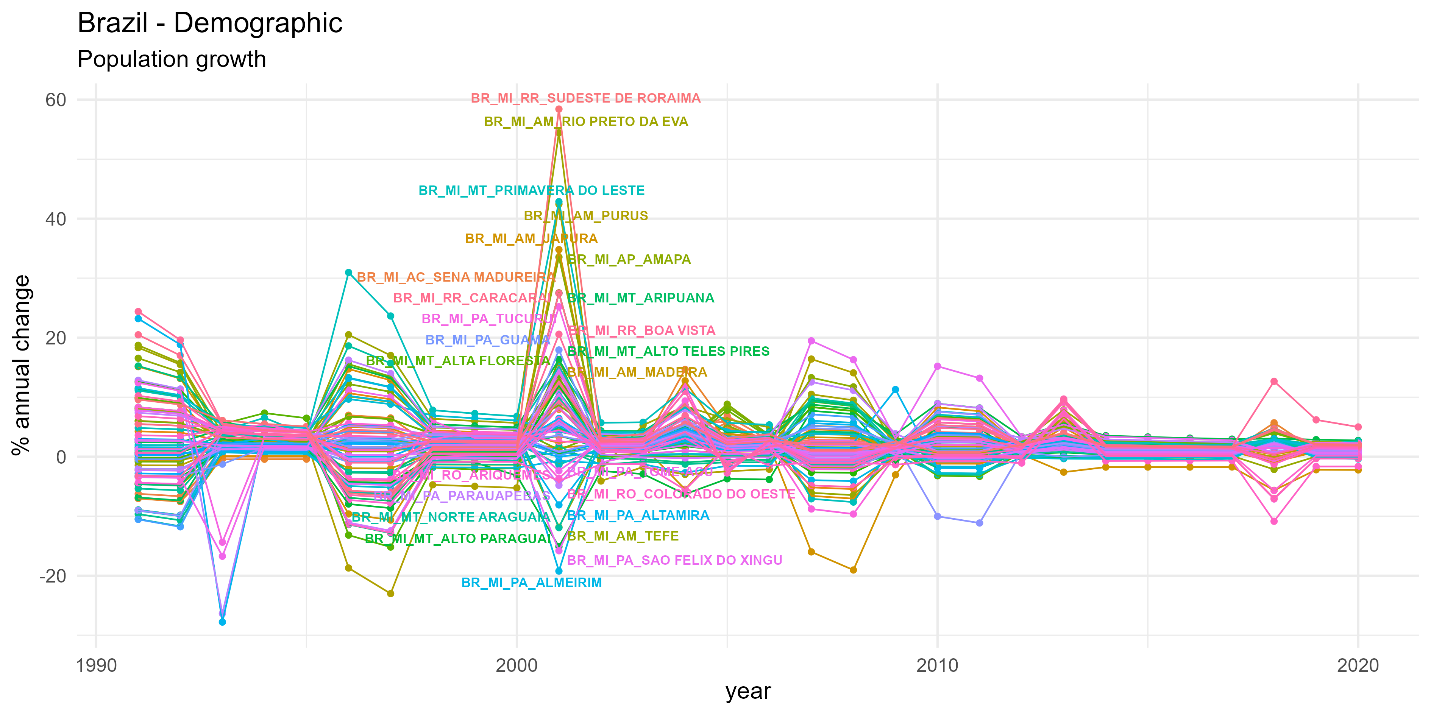 |

**Figure S1.** **Population in Brazil.** Total population and annual population percentage change for Brazil for the period 1990-2020. Migration processes linked to access to land, life cycles of previous migrants, economic incentives, among other have driven spikes of migration across the Brazilian amazon (Browder et al. 2008; Randell and VanWey 2014).

| 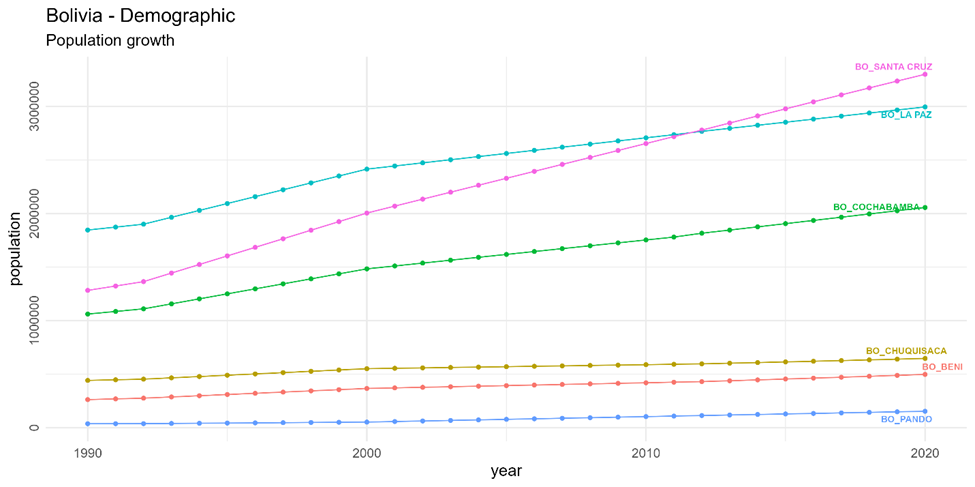 |
| --- |
| 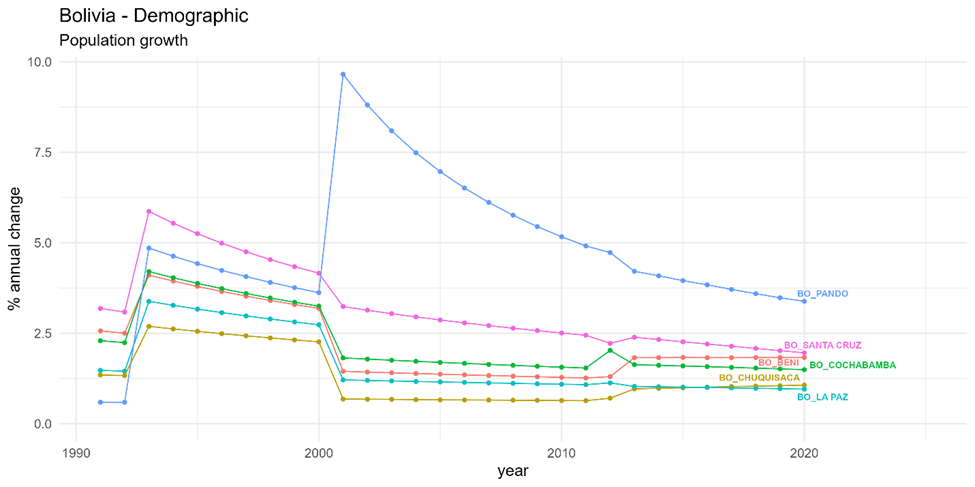 |

**Figure S2.** **Population in Bolivia.** Total population and annual population percentage change for Bolivia for the period 1990-2020. The department of Pando has been experiencing que largest growth rates due to immigration (Canelas et al. 2005).

| 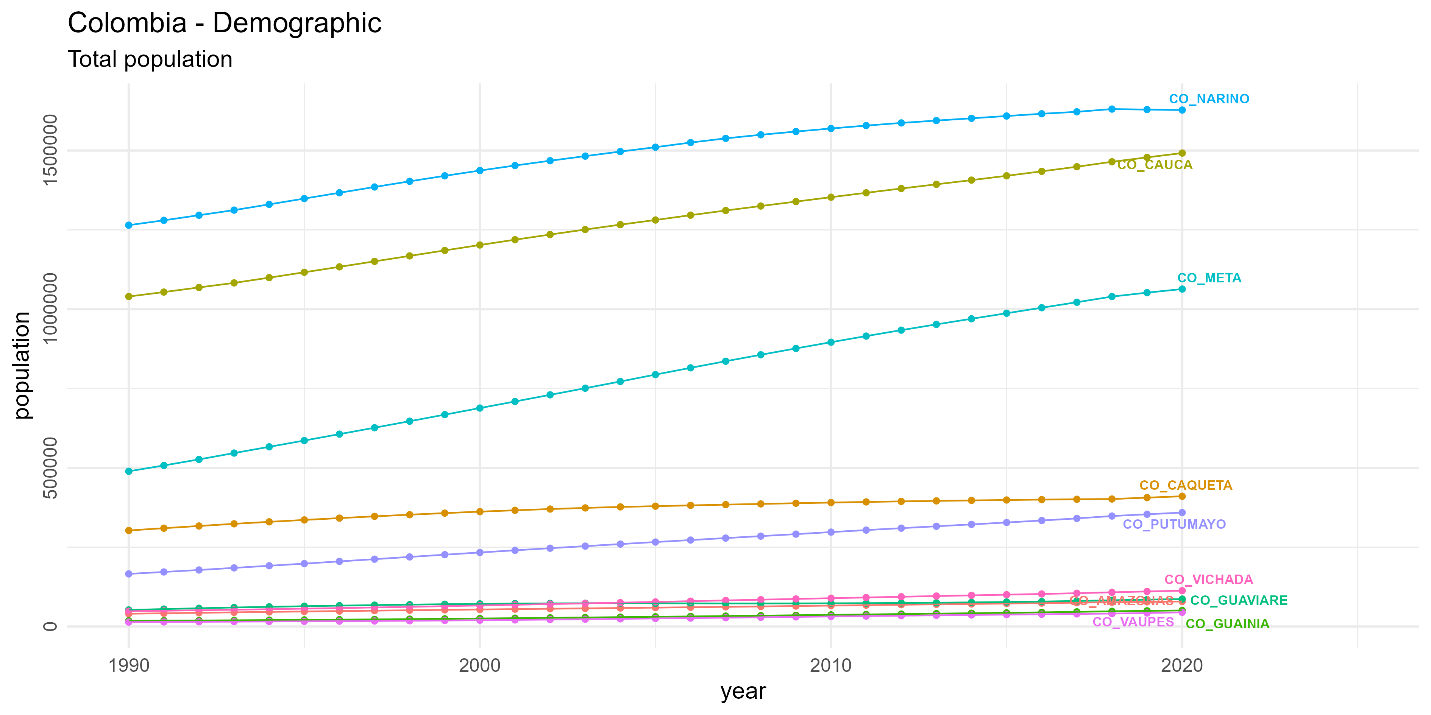 |
| --- |
| 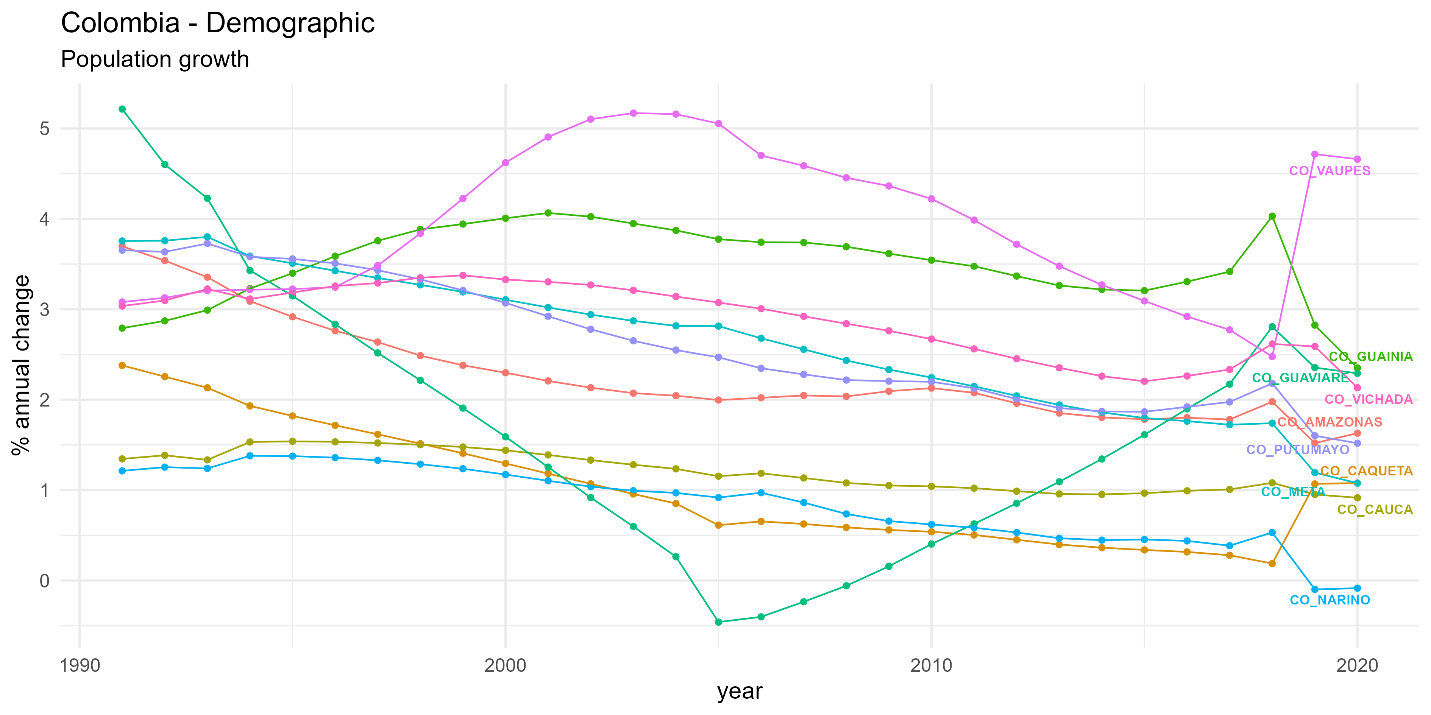 |

**Figure S3. Population in Colombia.** Total population and annual population percentage change for Colombia for the period 1990-2020.

| 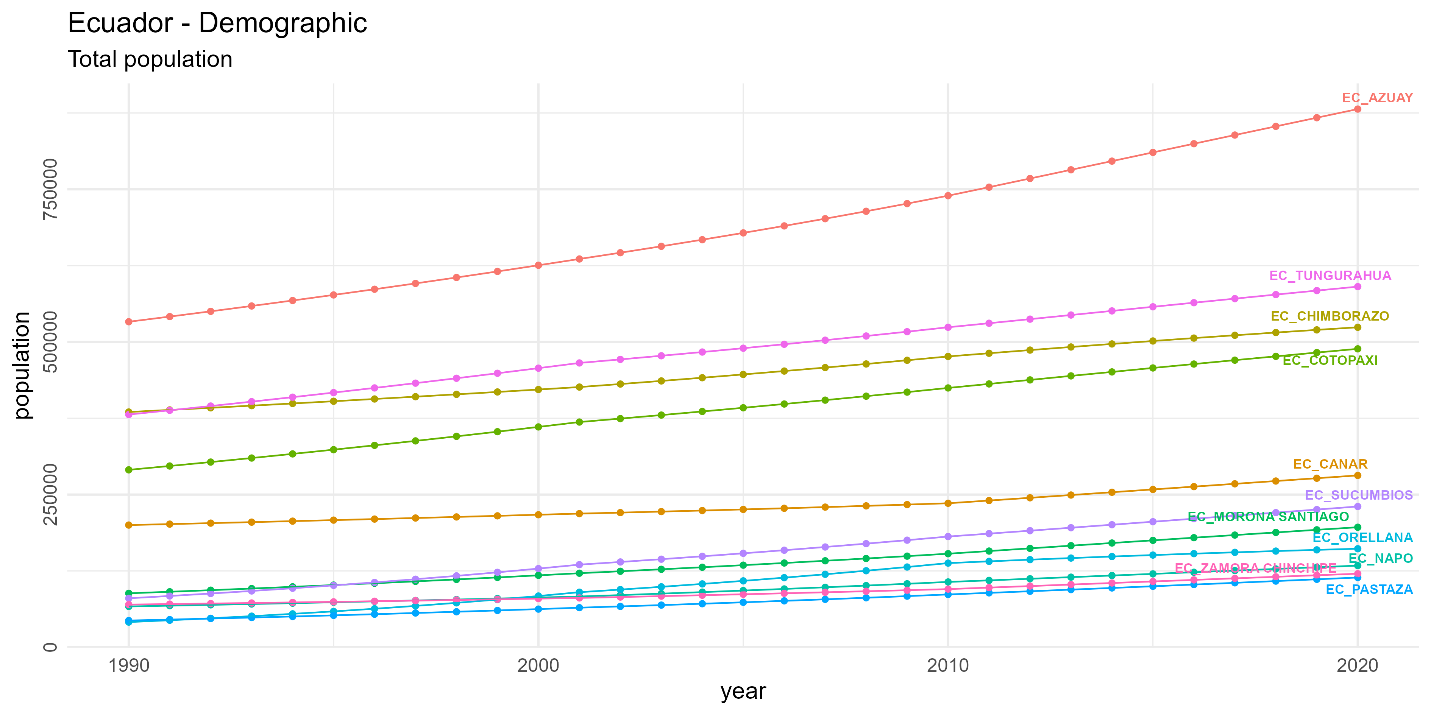 |
| --- |
| 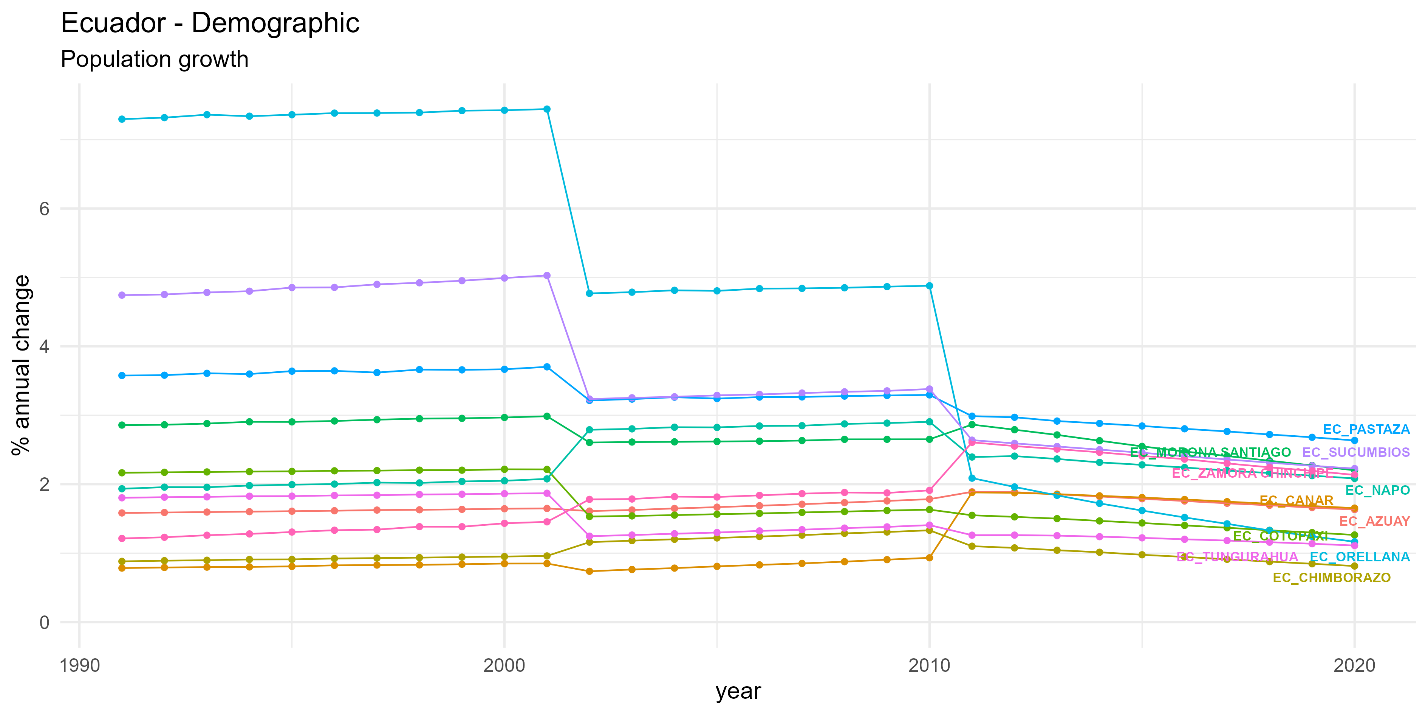 |

**Figure S4. Population in Ecuador.** Total population and annual population percentage change by province for Ecuador, for the period 1990-2020 for the period 1990-2020.

| 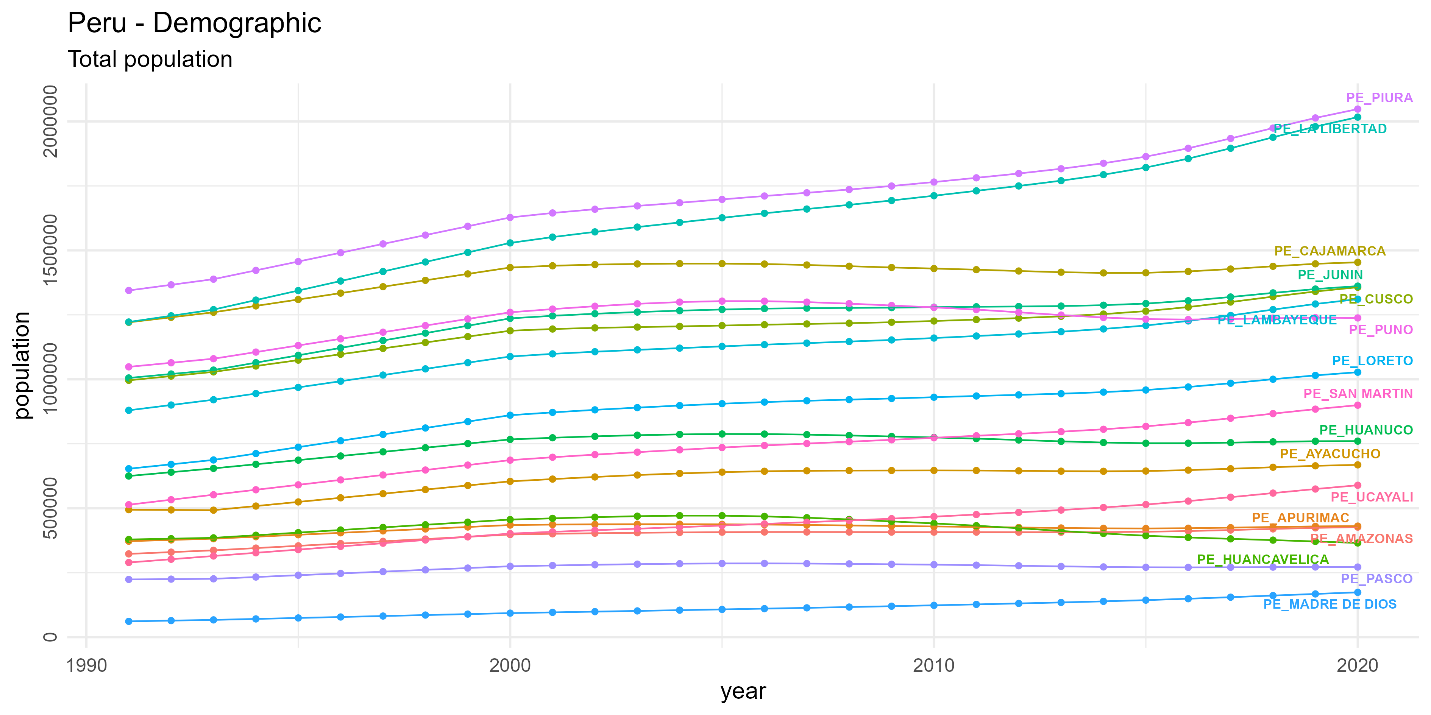 |
| --- |
| 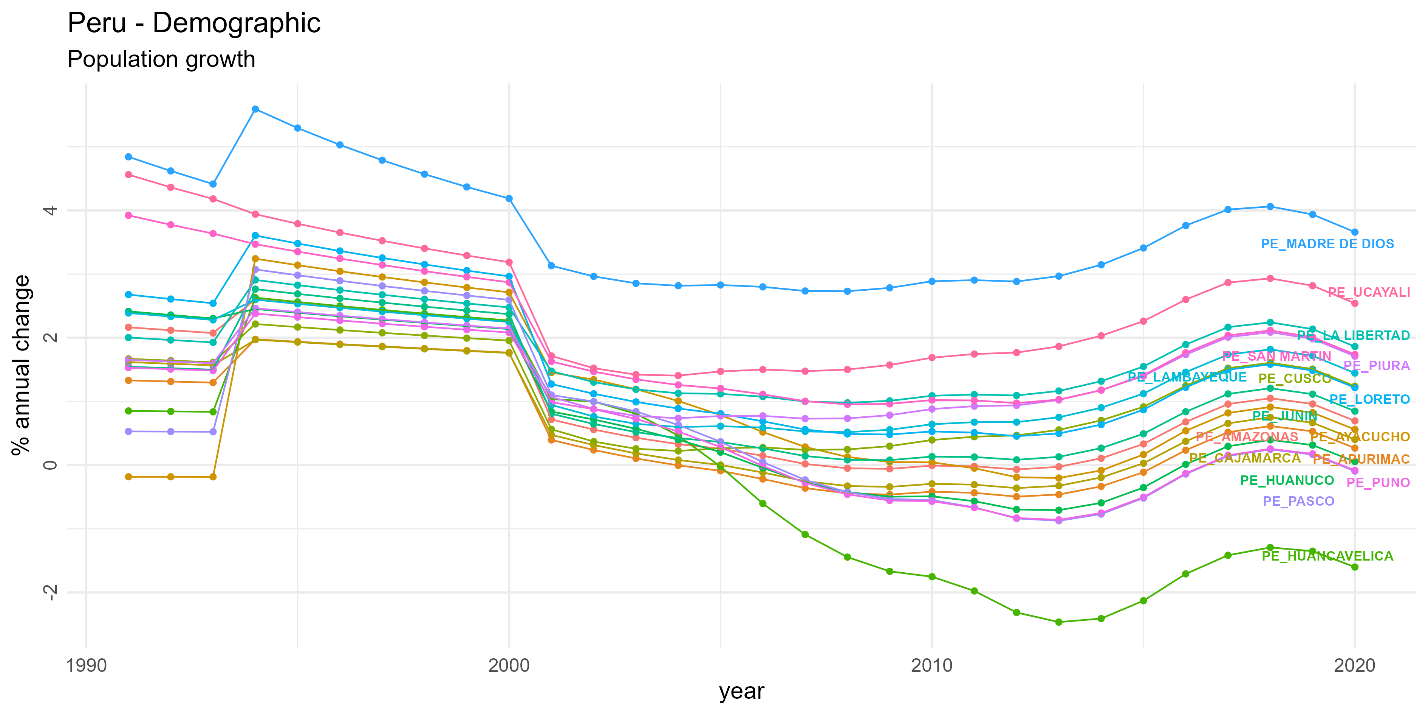 |

**Figure S5. Population in Peru.** Total population and annual population percentage change for Peru for the period 1990-2020.

| 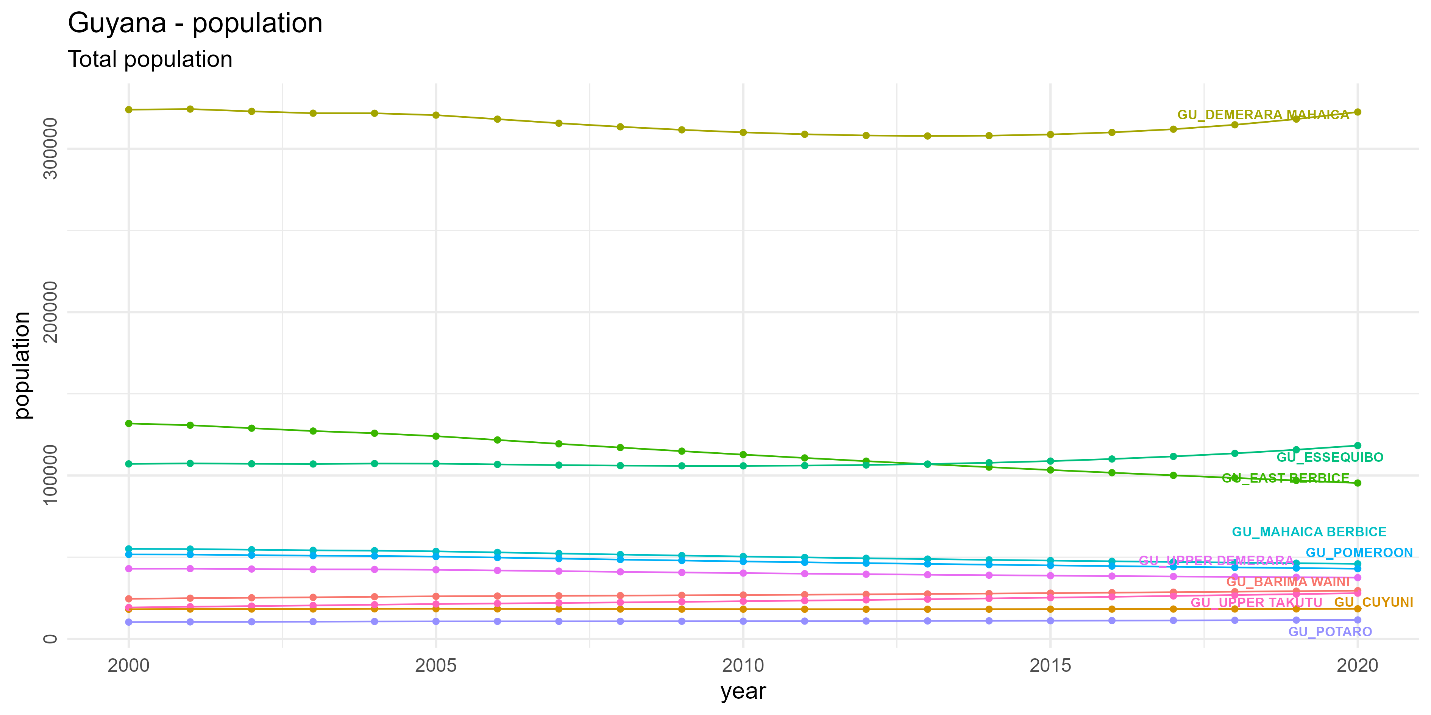 |
| --- |
| 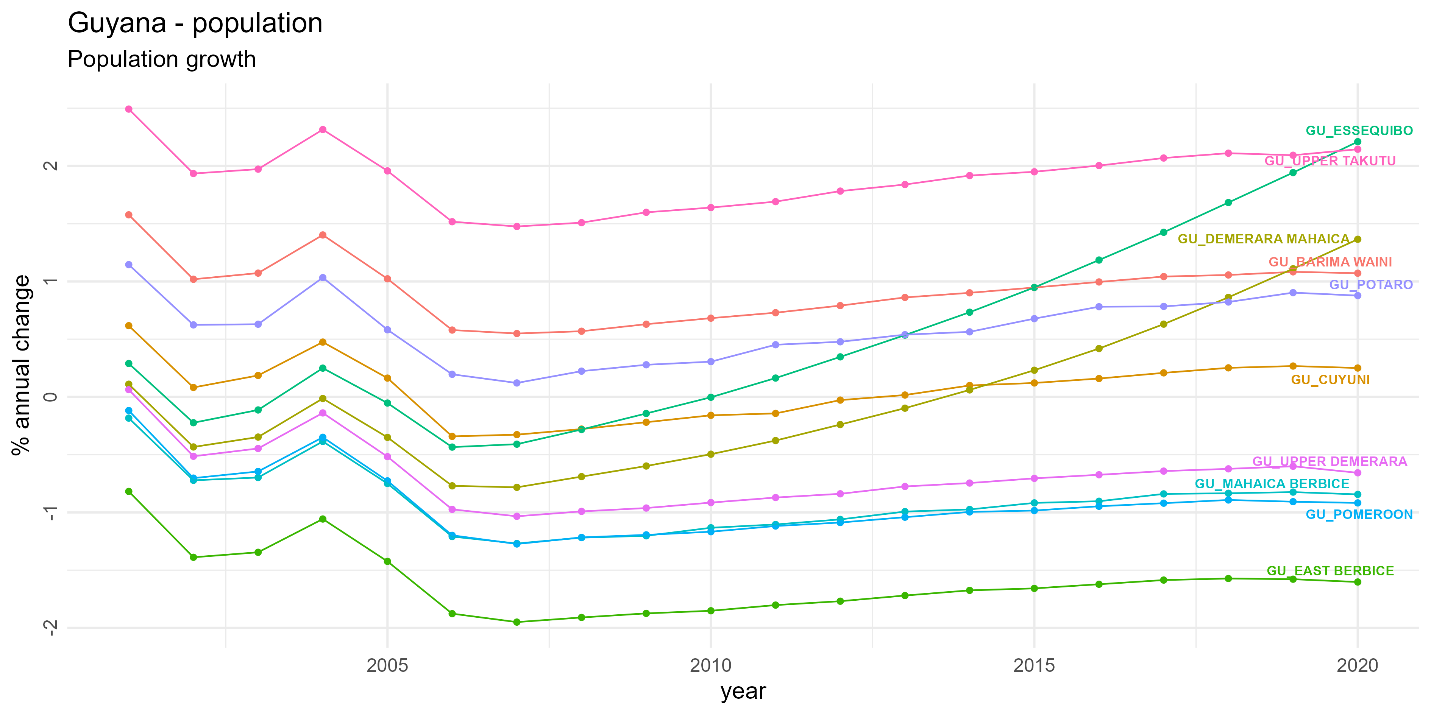 |

**Figure S6. Population in Guyana.** Total population and annual population percentage change for Guyana for the period 2000-2020.

| 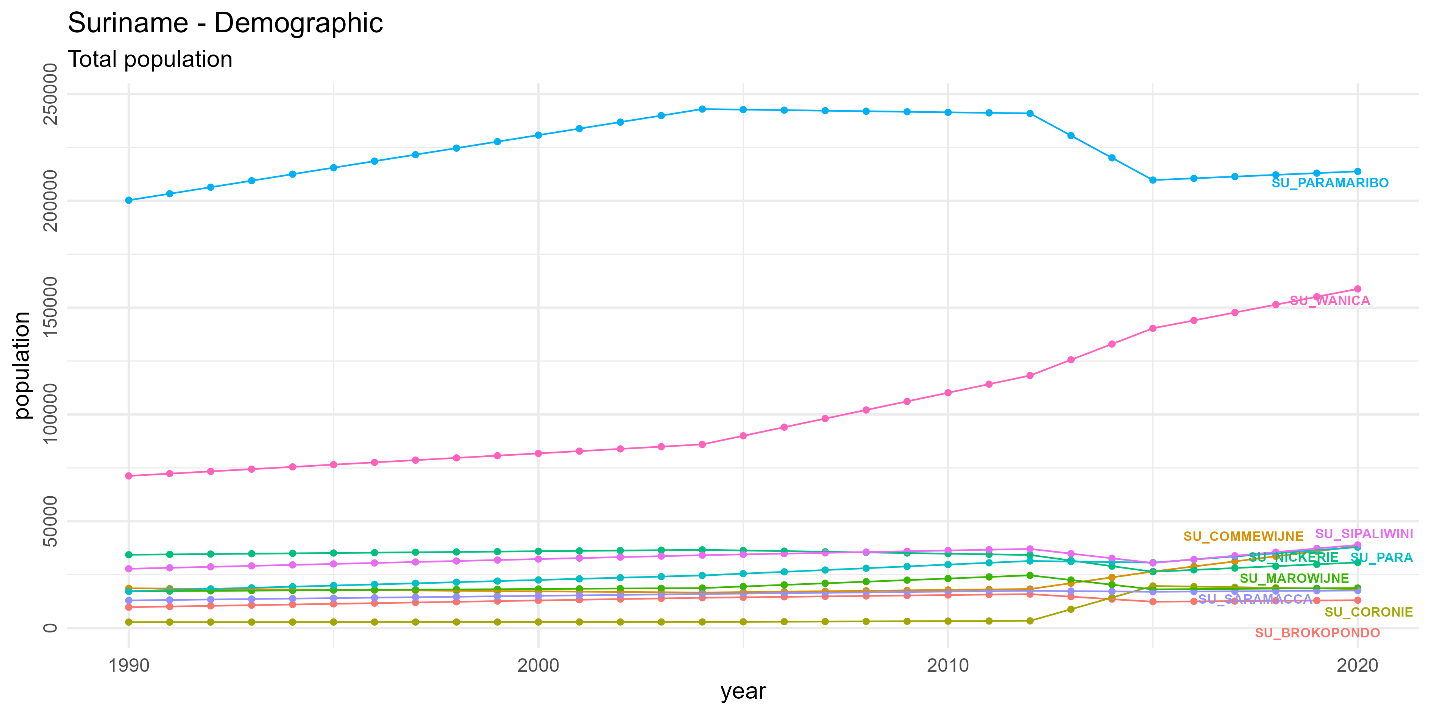 |
| --- |
| 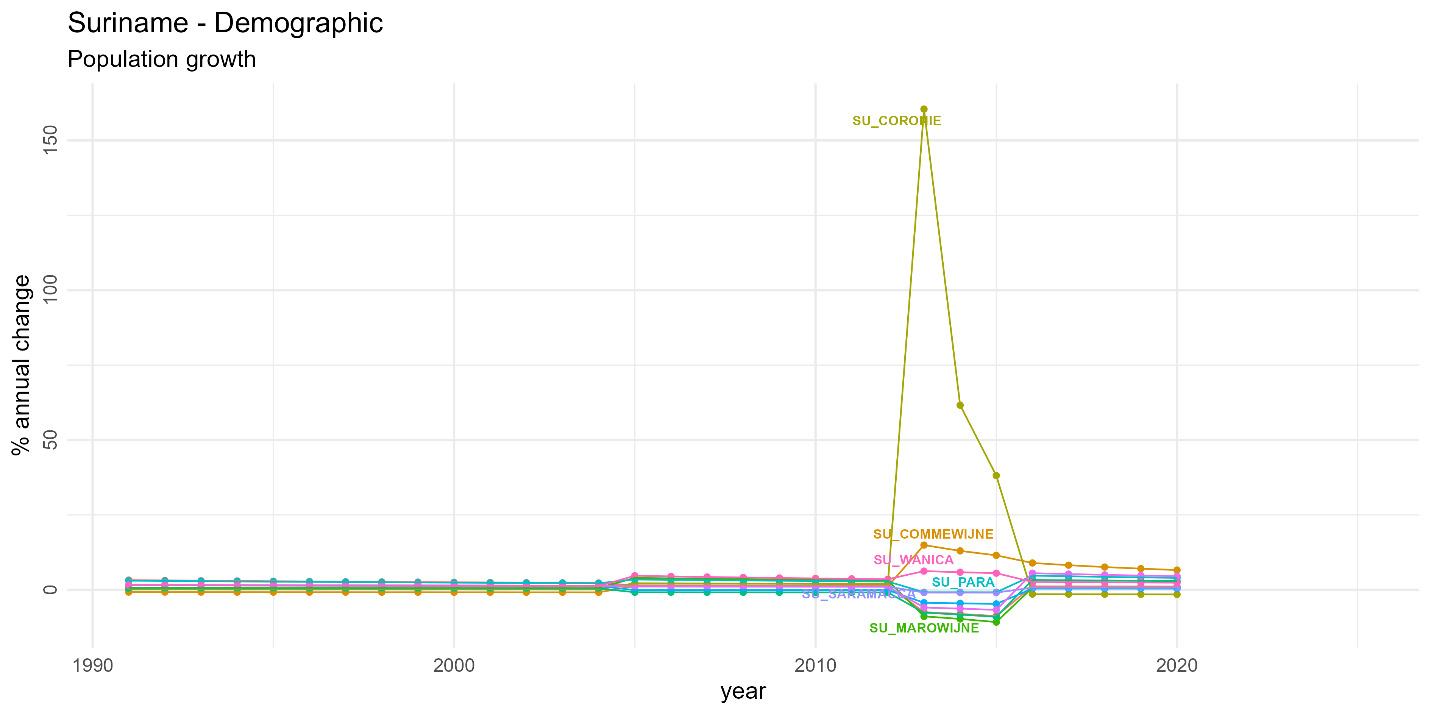 |

**Figure S7. Population in Suriname.** Total population and annual population percentage change for Suriname for the period 1990-2020.

| 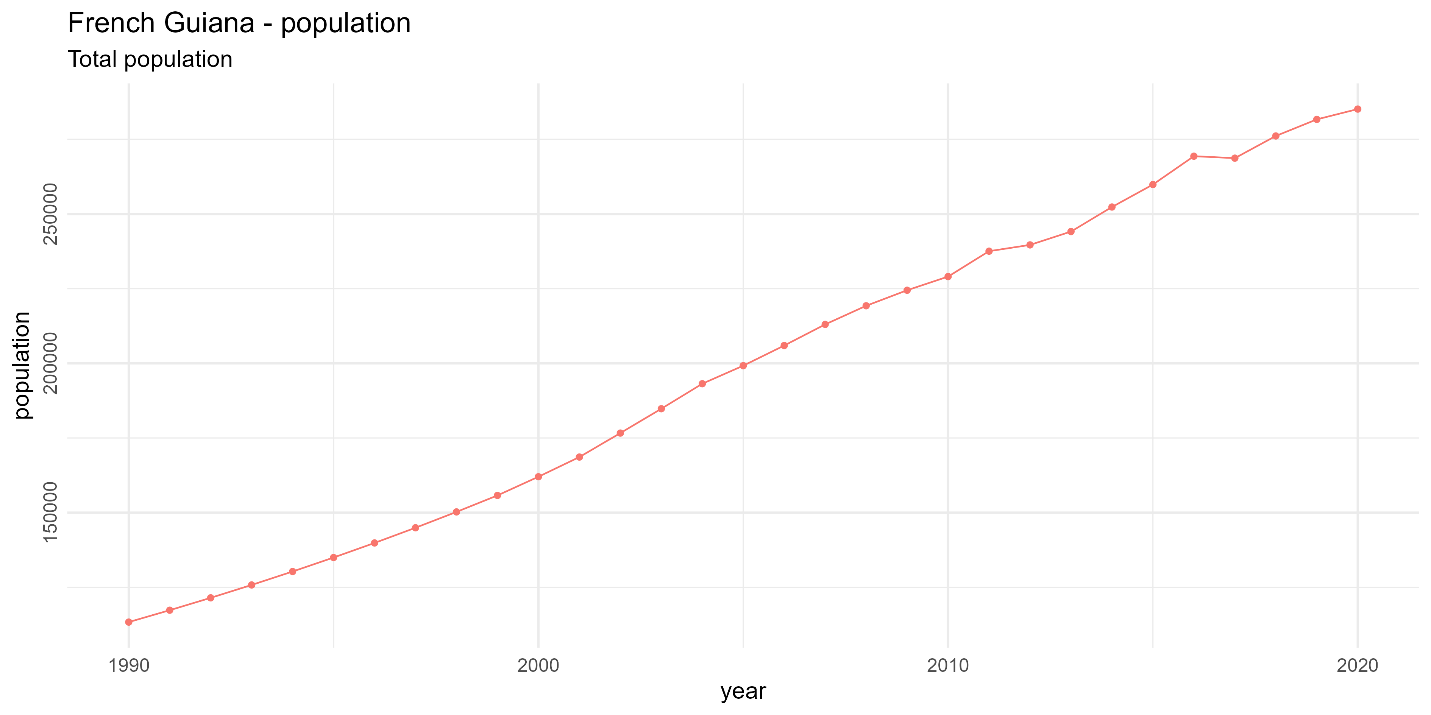 |
| --- |
| 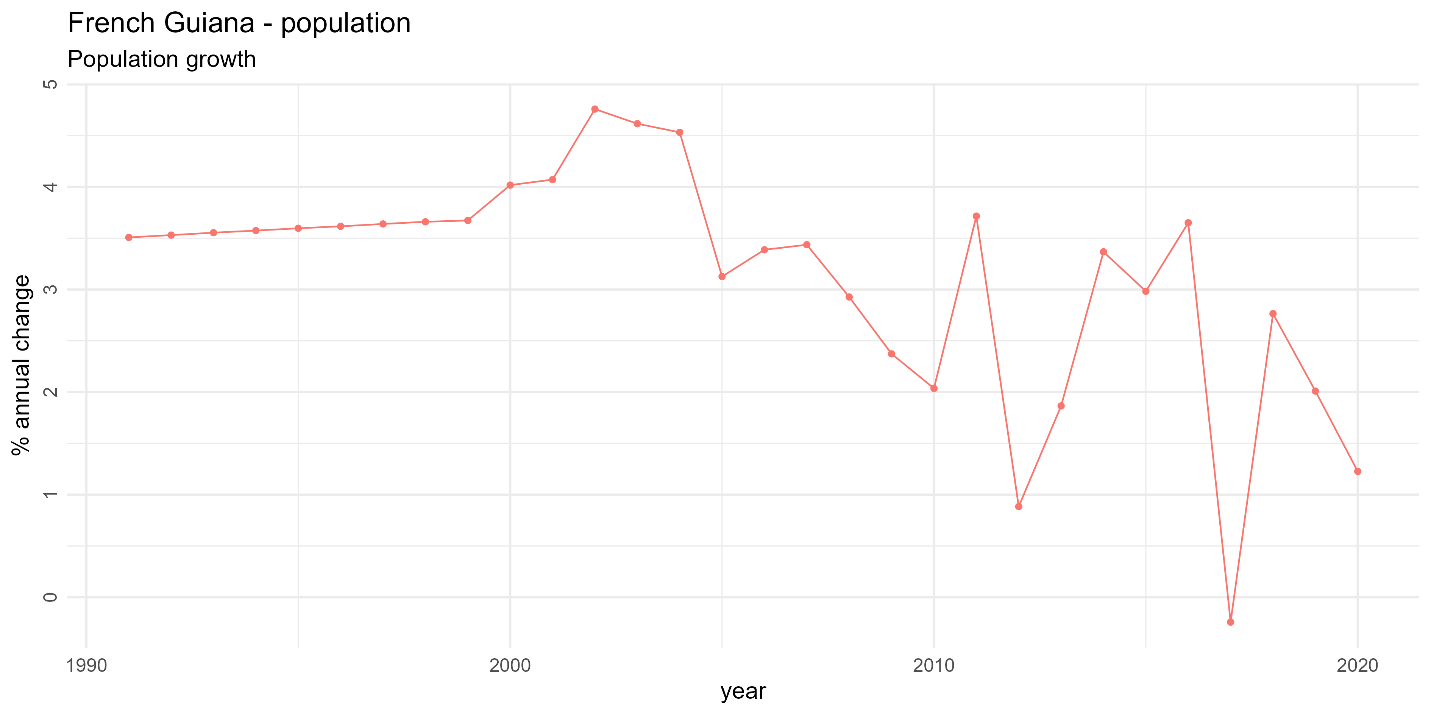 |

**Figure S8. Population in French Guiana.** Total population and annual population percentage change for French Guiana for the period 1990-2020.

| 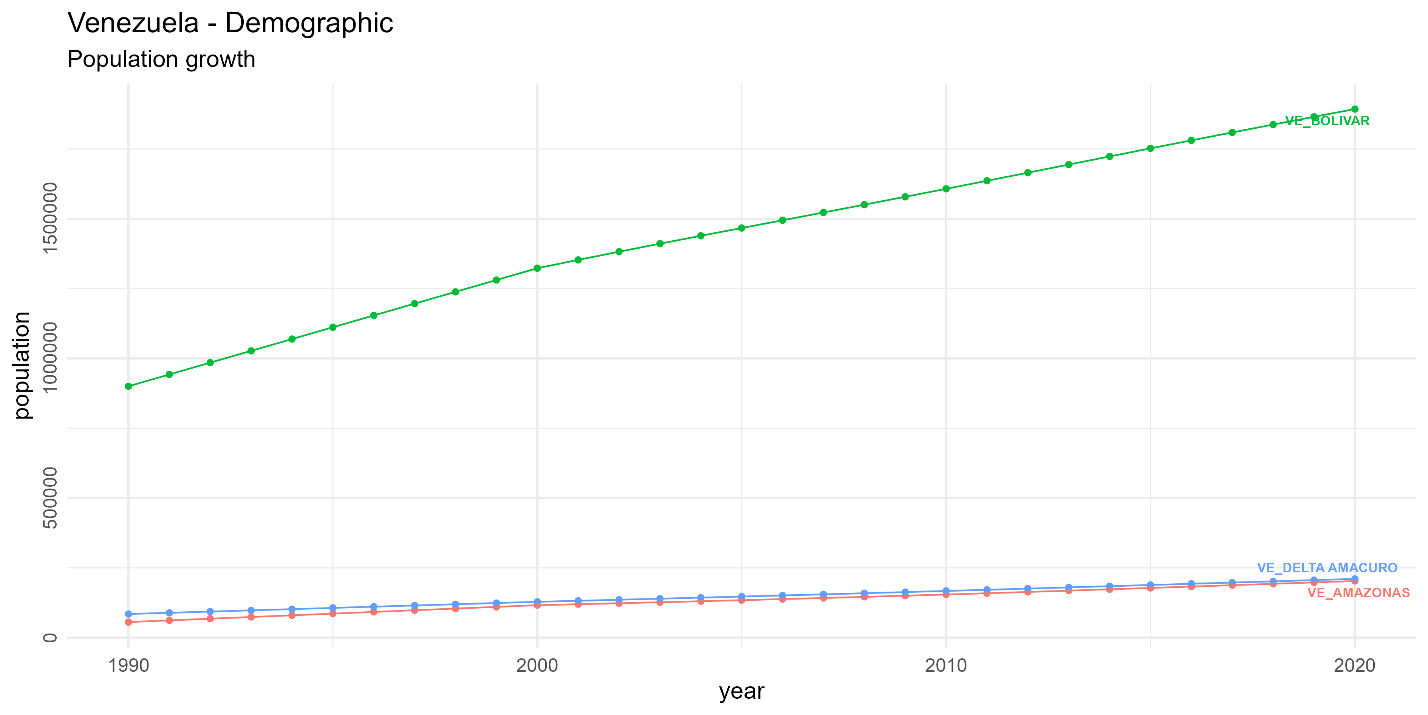 |
| --- |
| 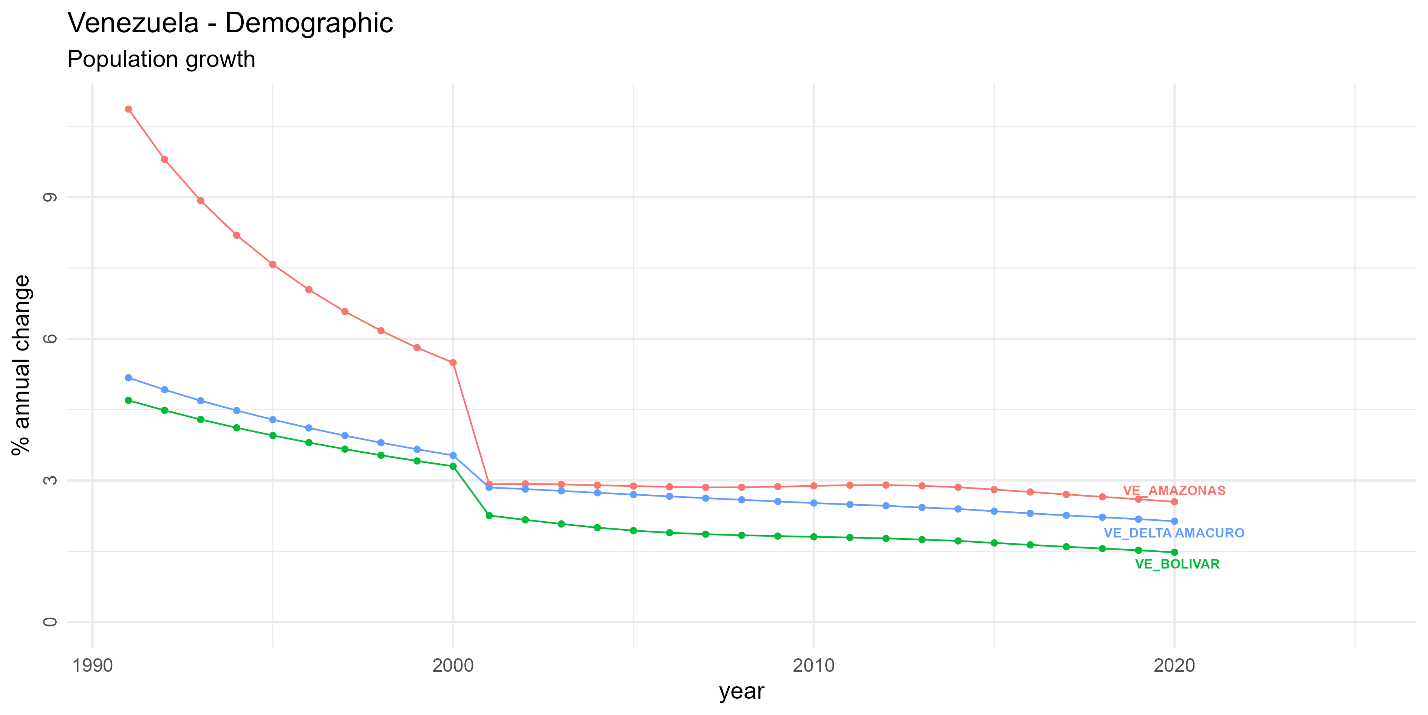 |

**Figure S9. Population in Venezuela.** Total population and annual population percentage change for Venezuela for the period 1990-2020.

### Agriculture


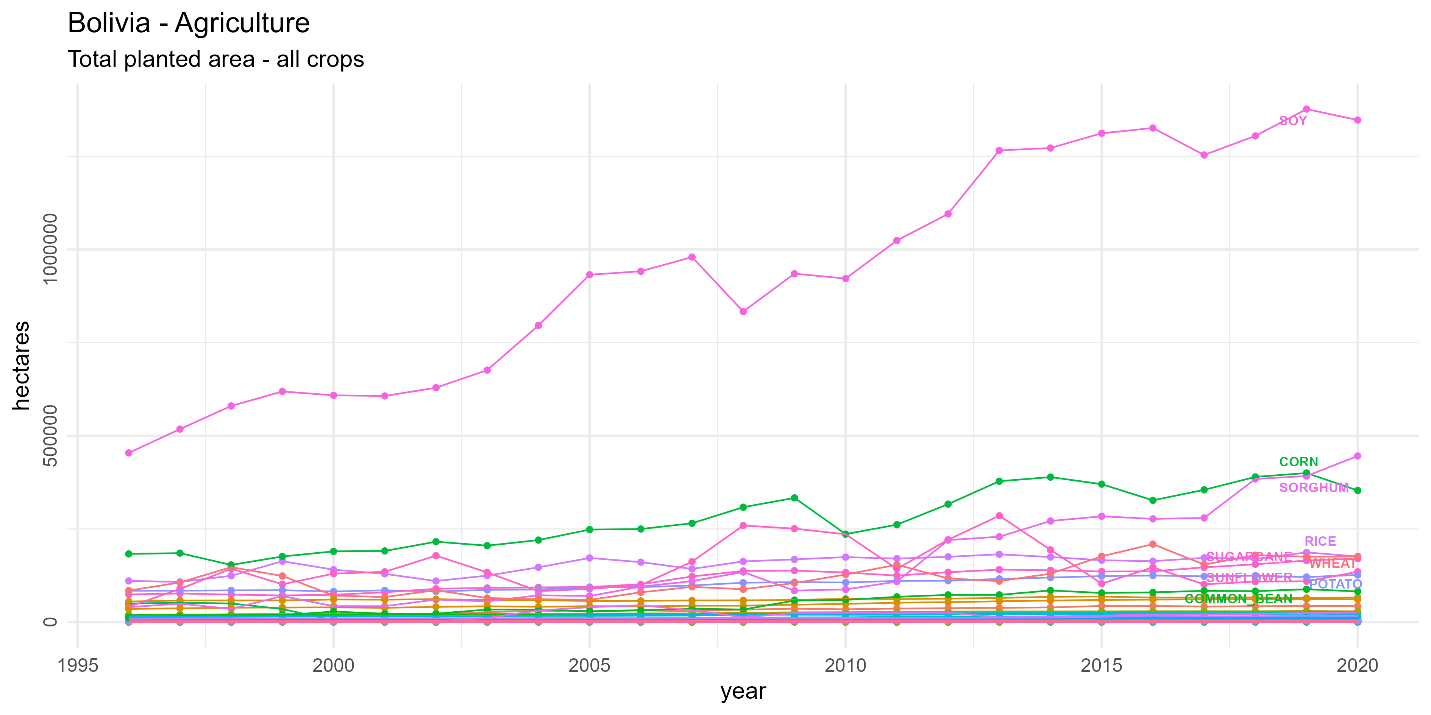


**Figure S10. Agriculture in Bolivia.** Total planted area for all reported crops in Bolivia for the period 1996-2020.

| 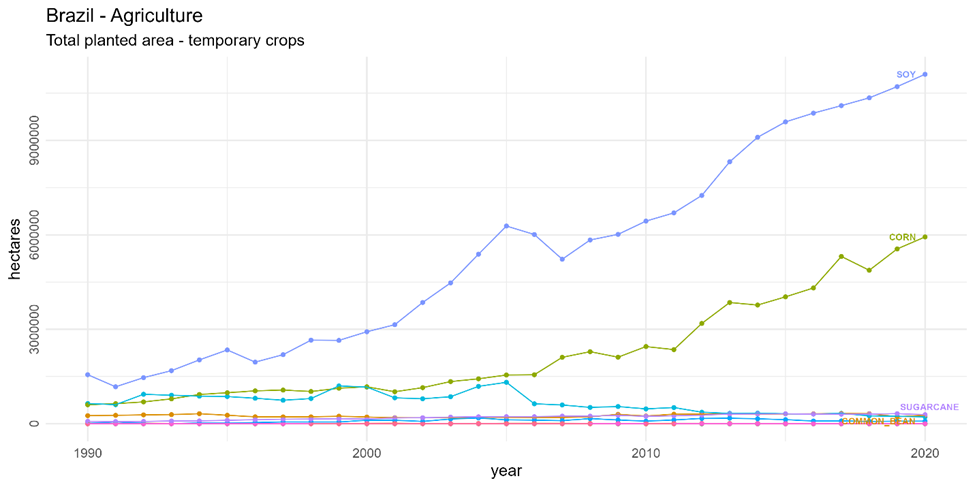 |
| --- |
| 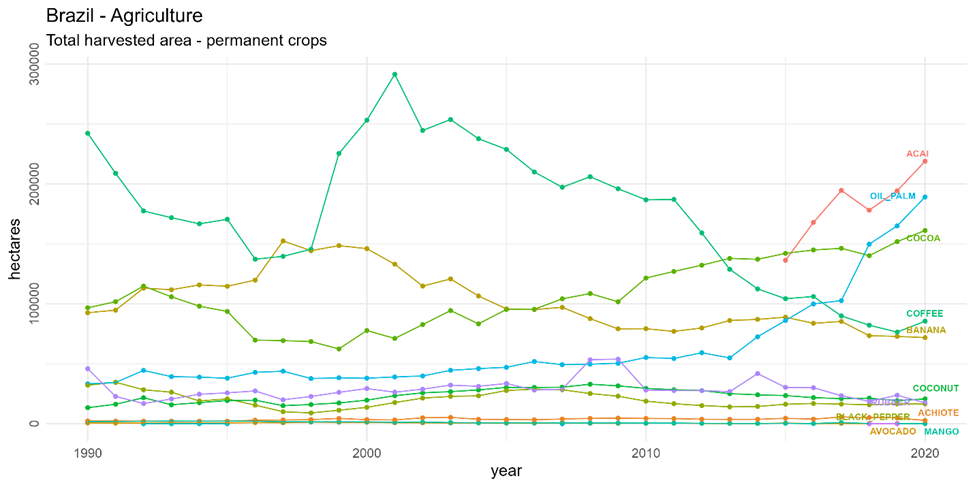 |

**Figure S11. Agriculture in Brazil.** Total planted area for all reported temporary crops and total harvested area for all reported permanent crops in Brazil for the period 1990-2020.

| 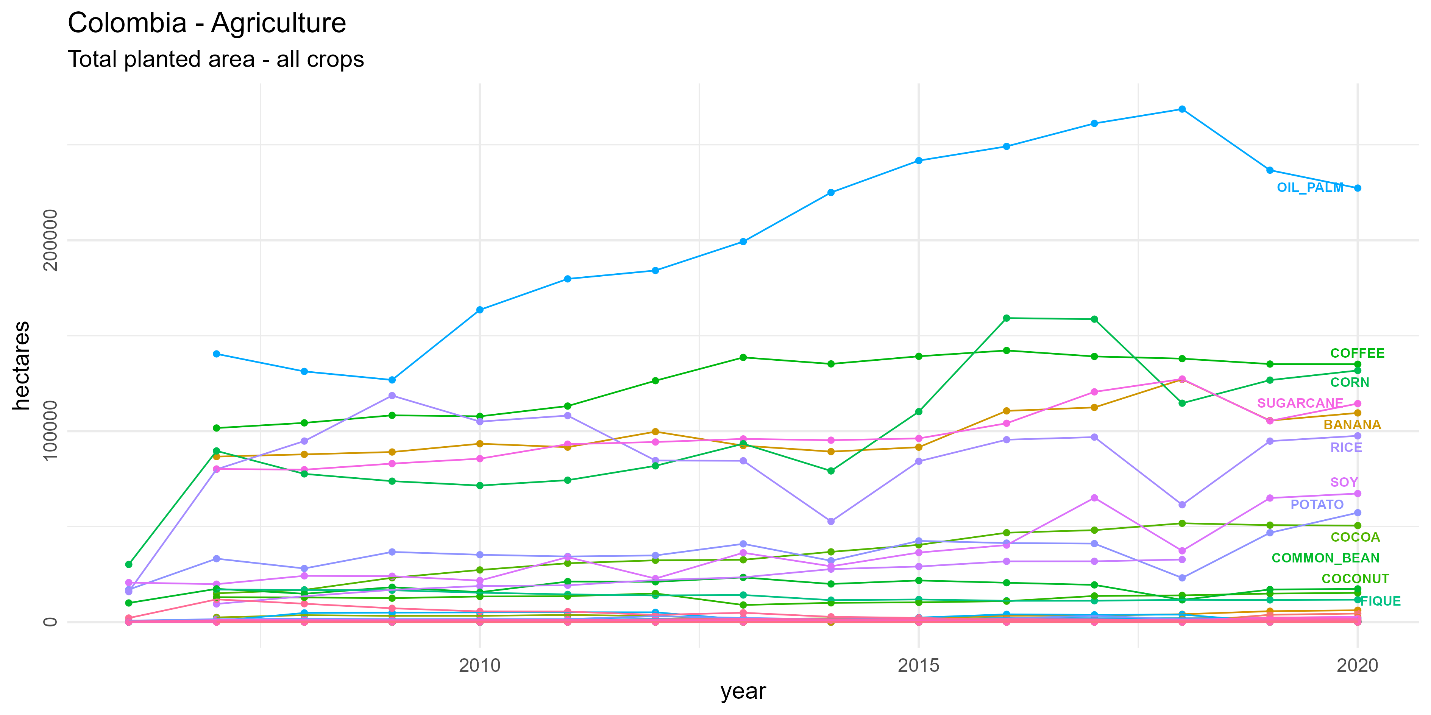 |
| --- |
| 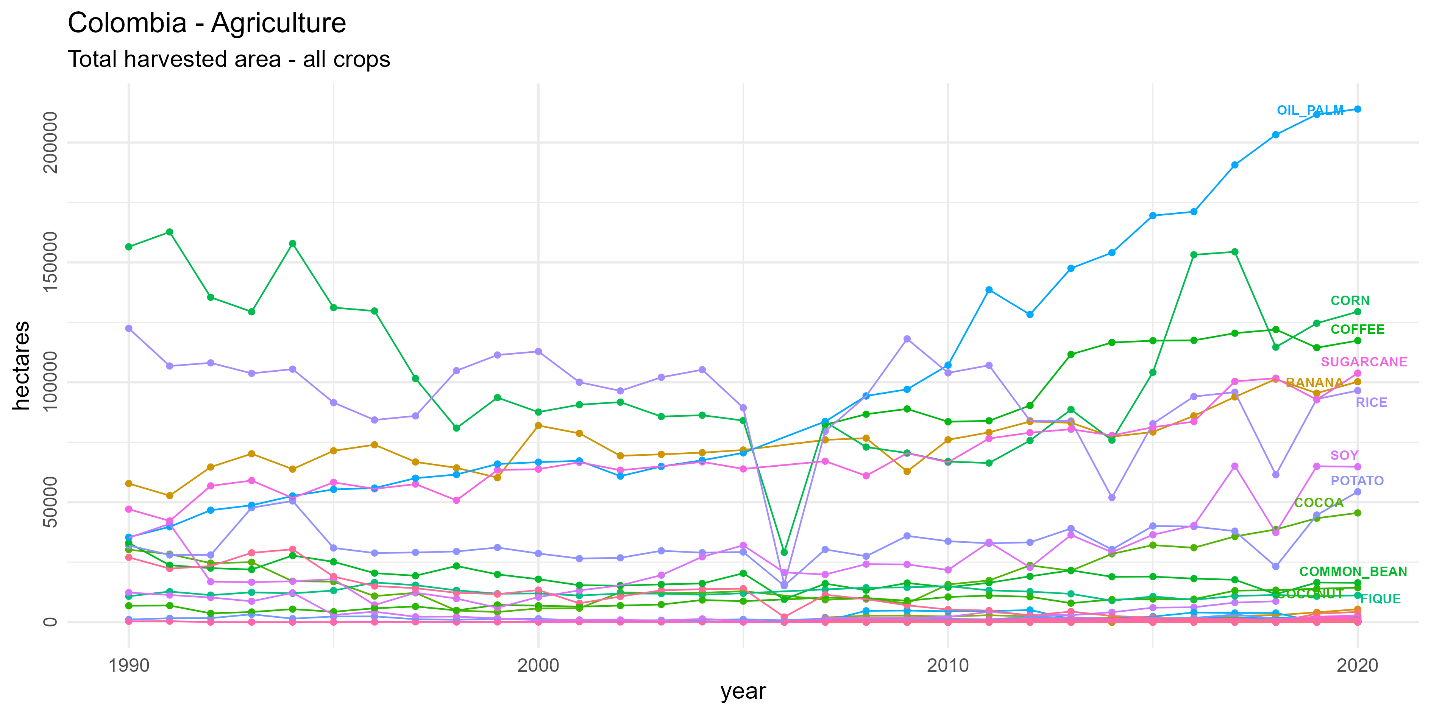 |

**Figure S12. Agriculture in Colombia.** Total planted area for the period 2006-2020 and total harvested area for the period 1996-2020 for crops reported in Colombia.


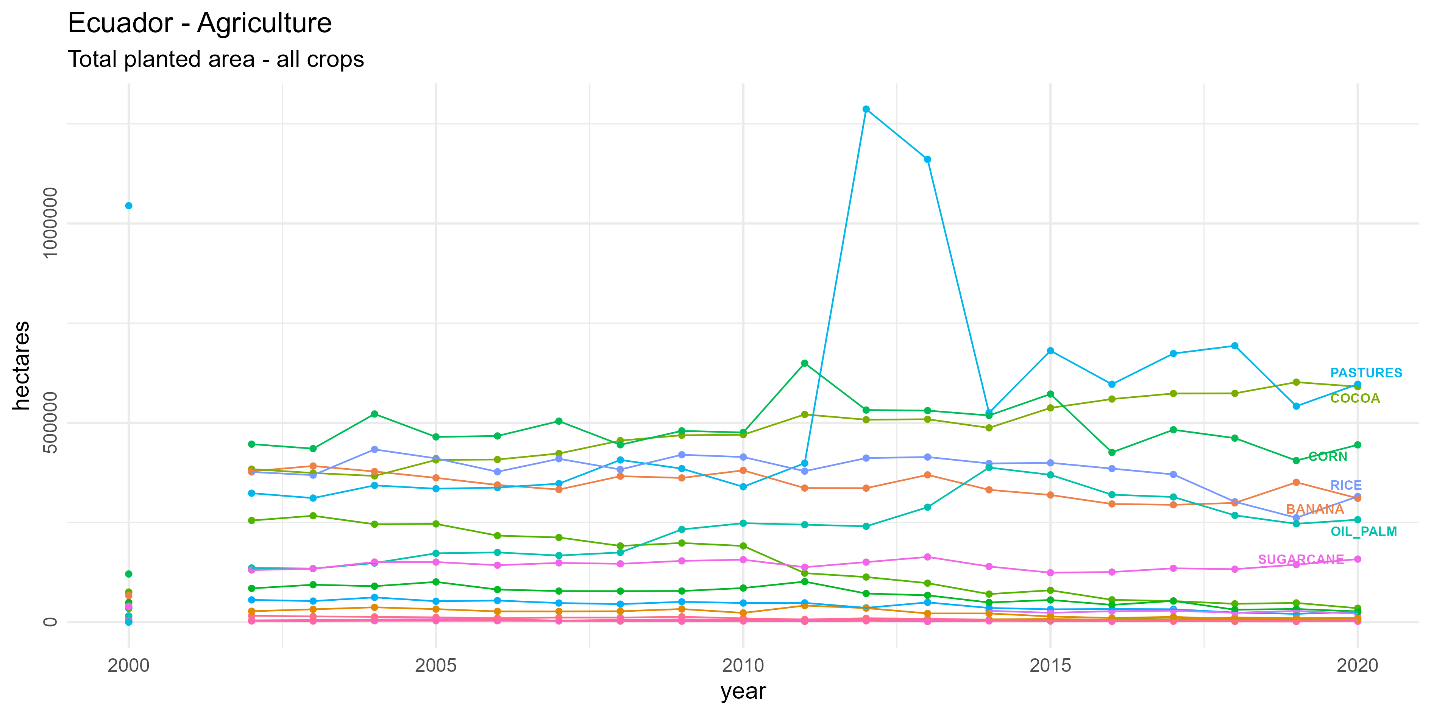


**Figure S13. Agriculture in Ecuador.** Total planted area for all reported crops in Ecuador for the period 2000-2020.

| 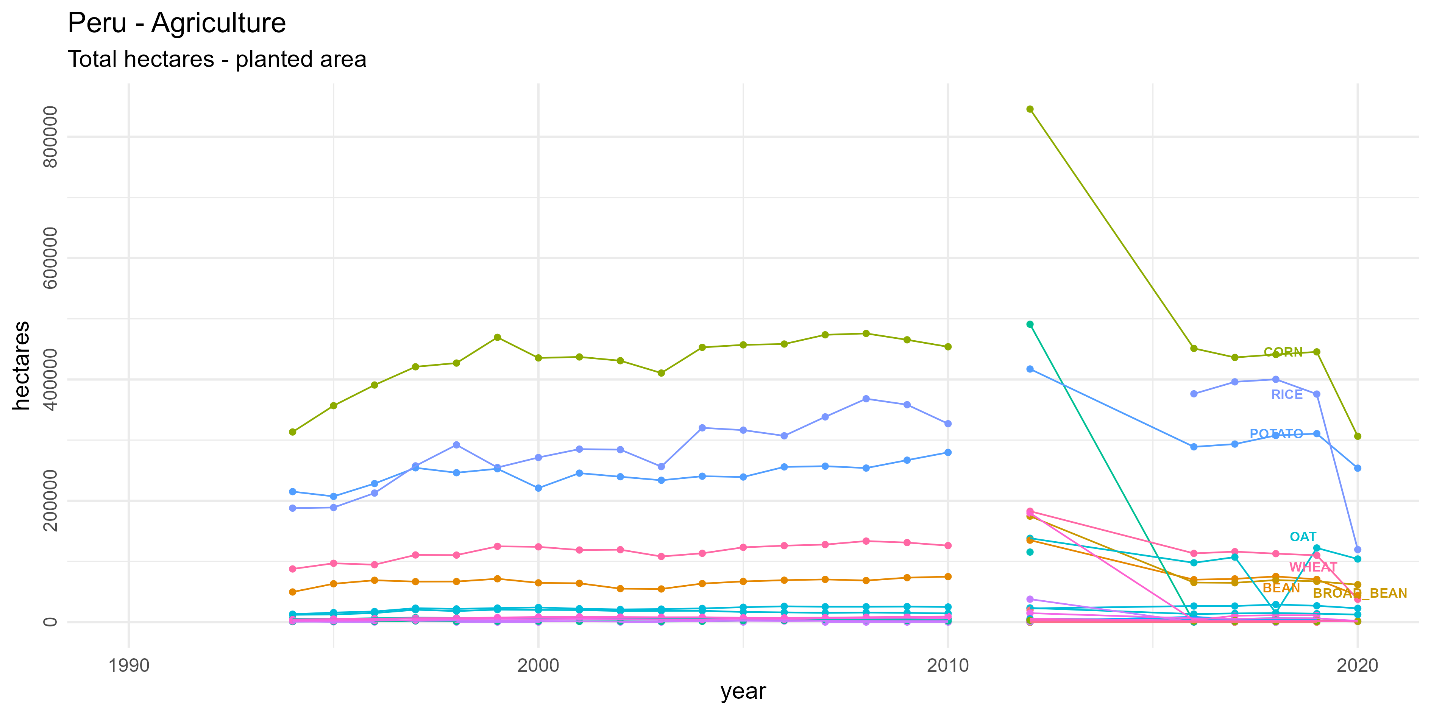 |
| --- |
| 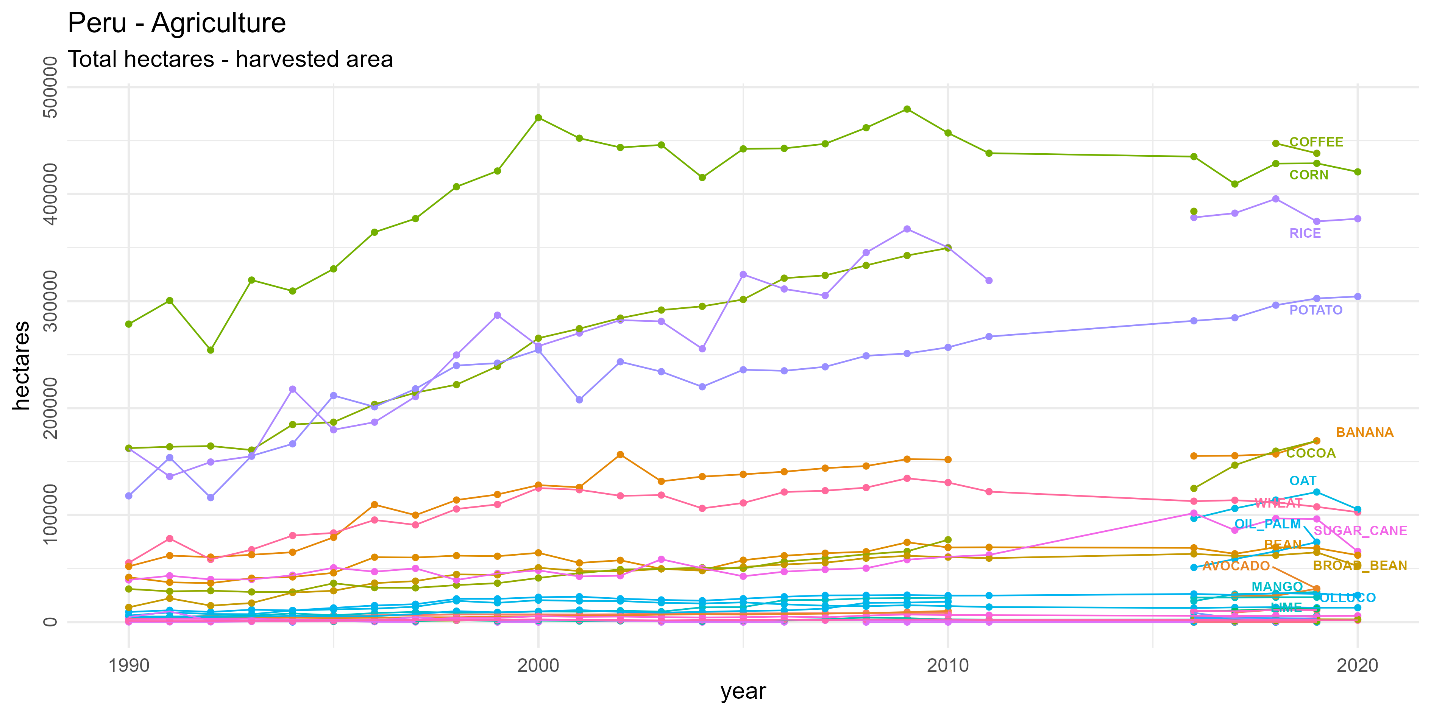 |

**Figure S14. Agriculture in Peru.** Total planted area for the period 1994-2020and total harvested area for the period 1990-2020 for crops reported in Peru. The peak in 2012 corresponds to the agricultural census.


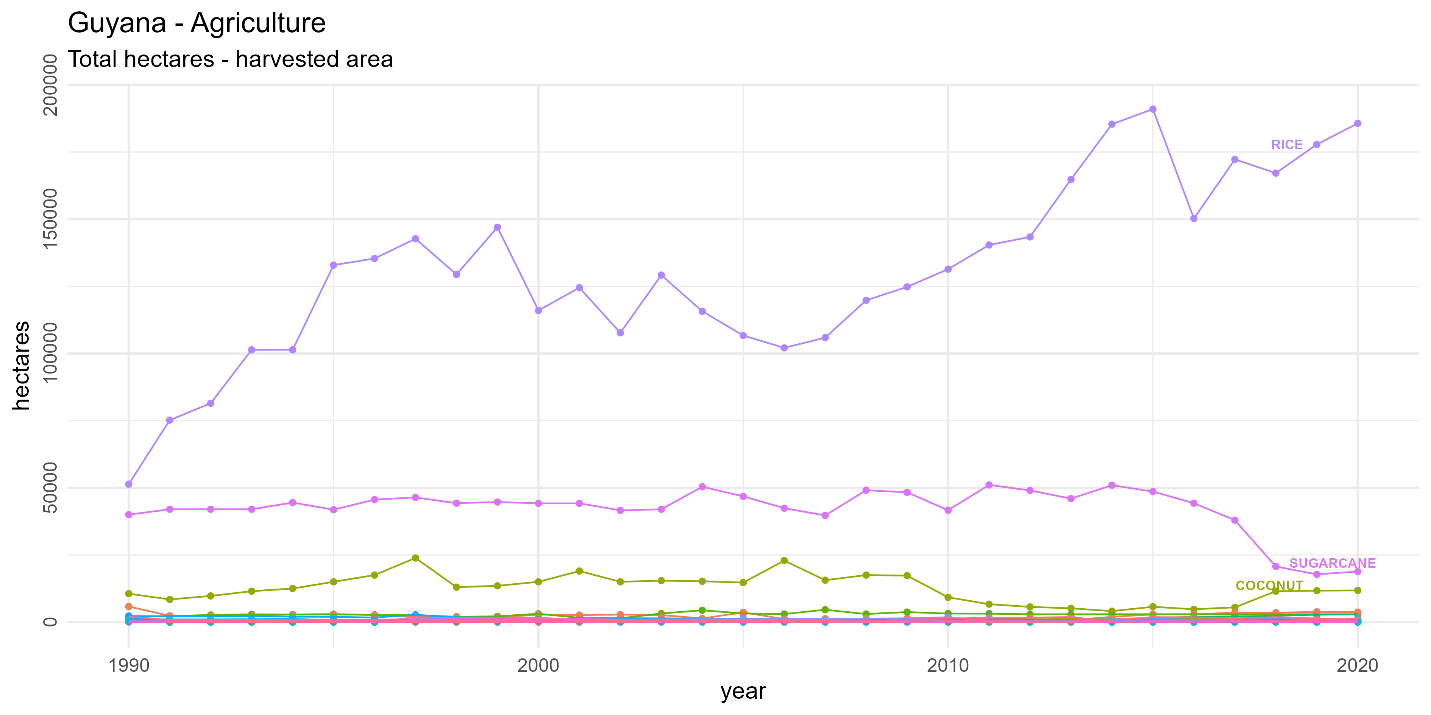


**Figure S15. Agriculture in Guyana.** Total harvested for all reported crops in Guyana for the period 1990-2020.


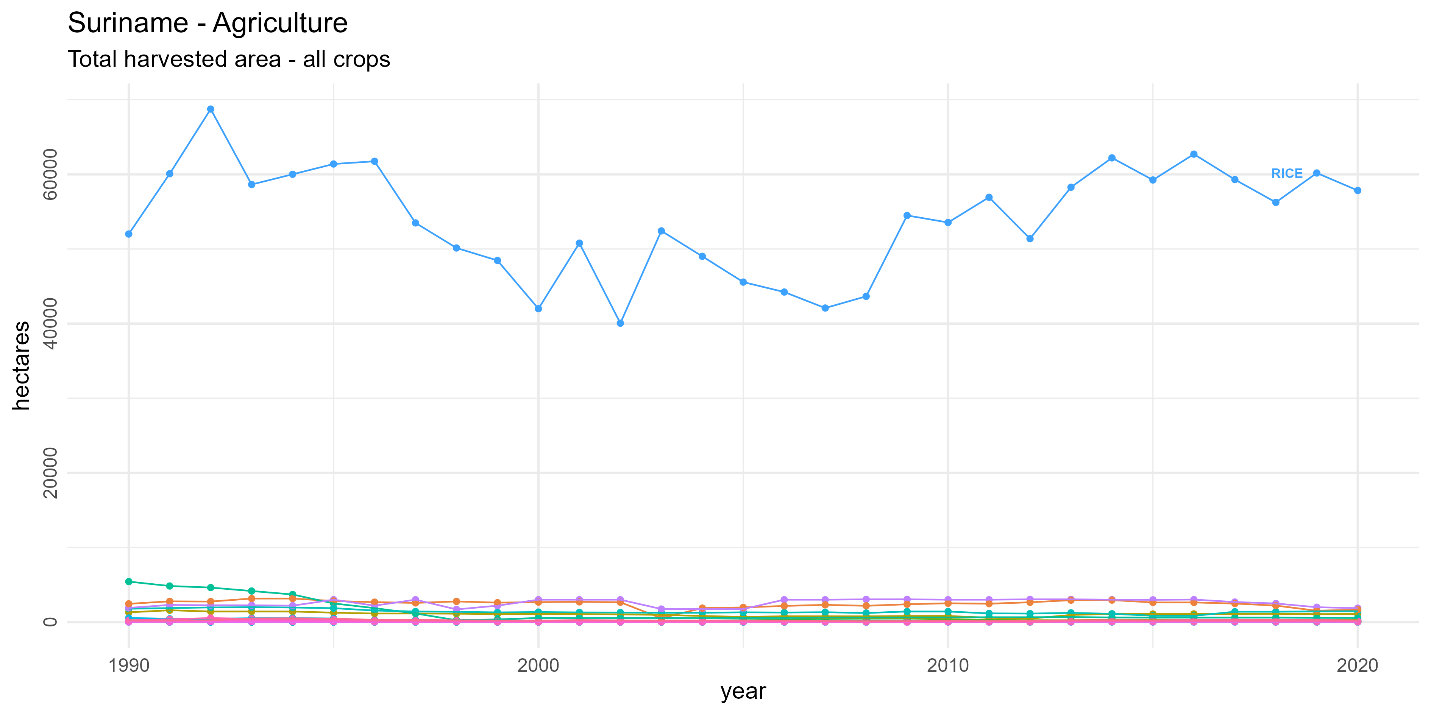


**Figure S16. Agriculture in Suriname.** Total harvested for all reported crops in Suriname for the period 1990-2020.


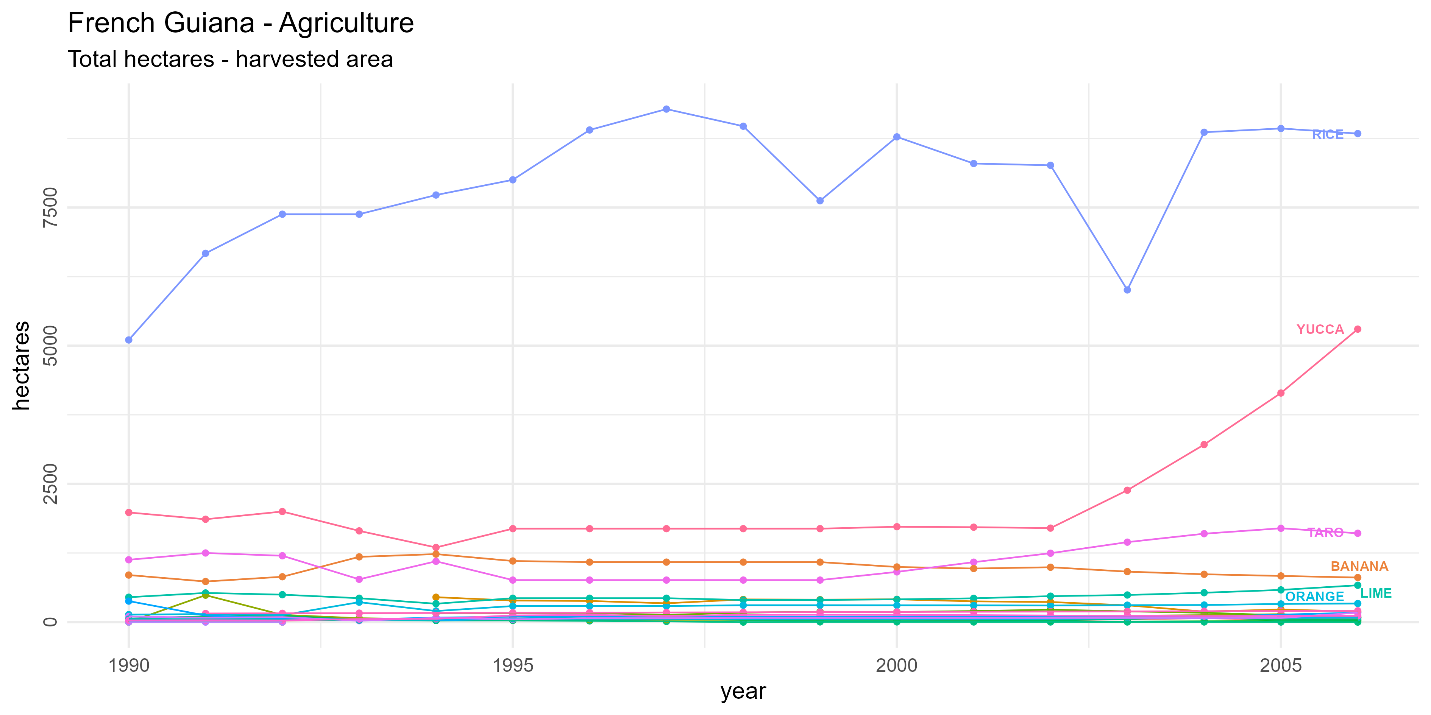


**Figure S17. Agriculture in French Guiana.** Total harvested for all reported crops in French Guiana for the period 1990-2006.

### Livestock


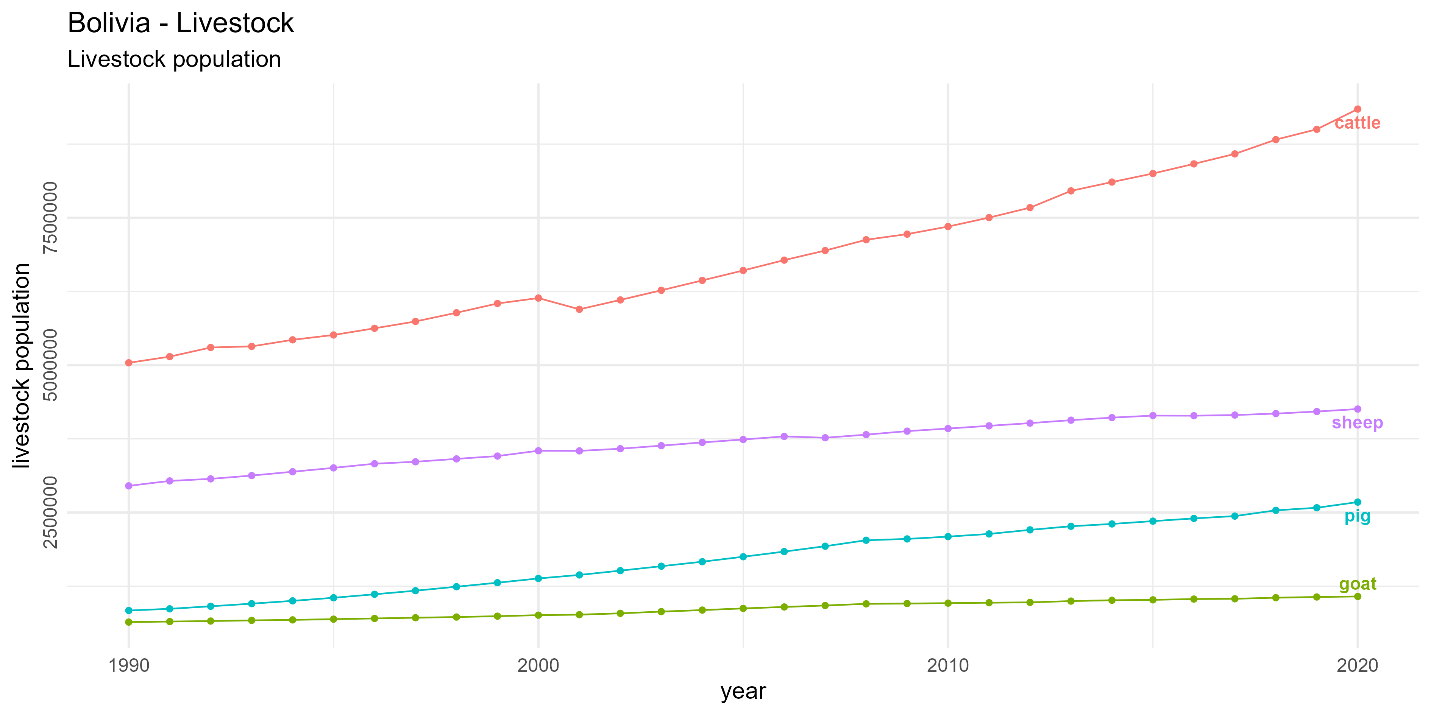


**Figure S18. Livestock in Bolivia.** Total livestock population for all major livestock species reported in Bolivia for the period 1990-2020.


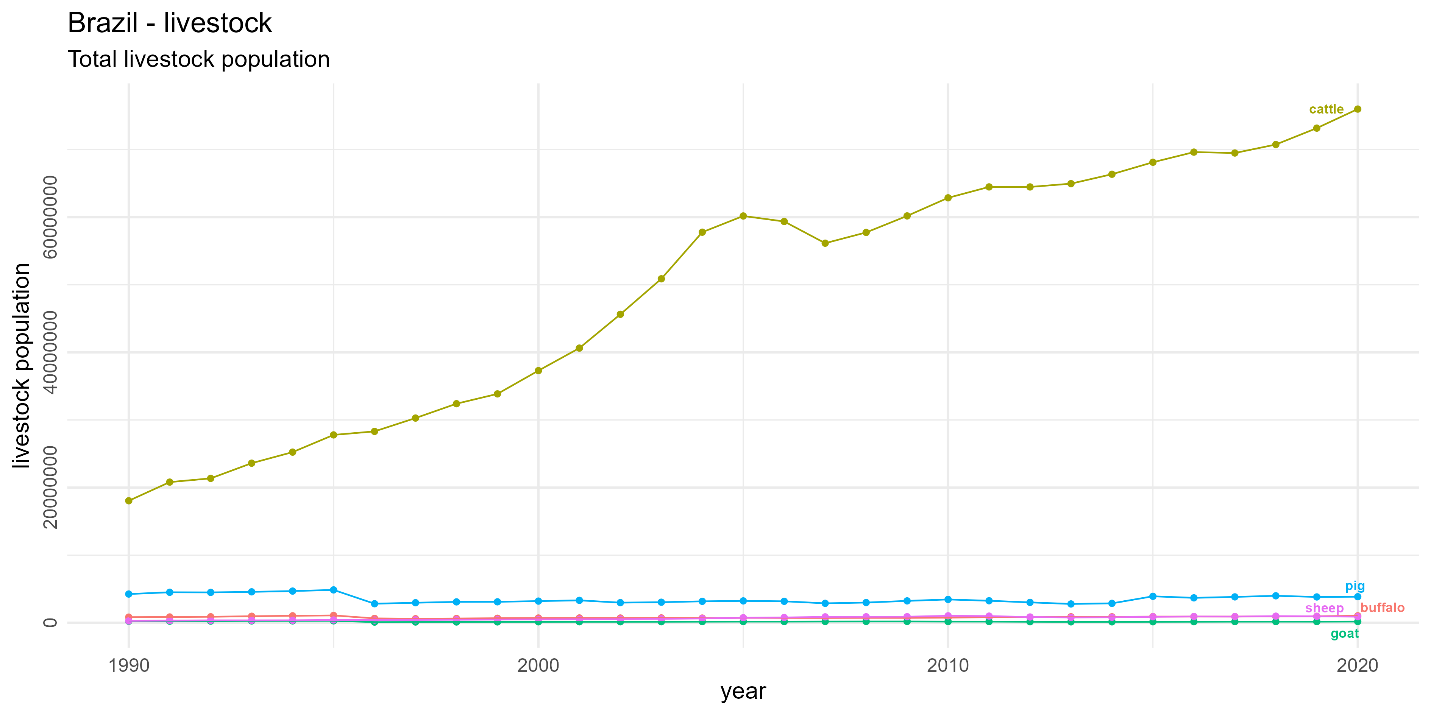


**Figure S19. Livestock in Brazil.** Total livestock population for all major livestock species reported in Brazil for the period 1990-2020.


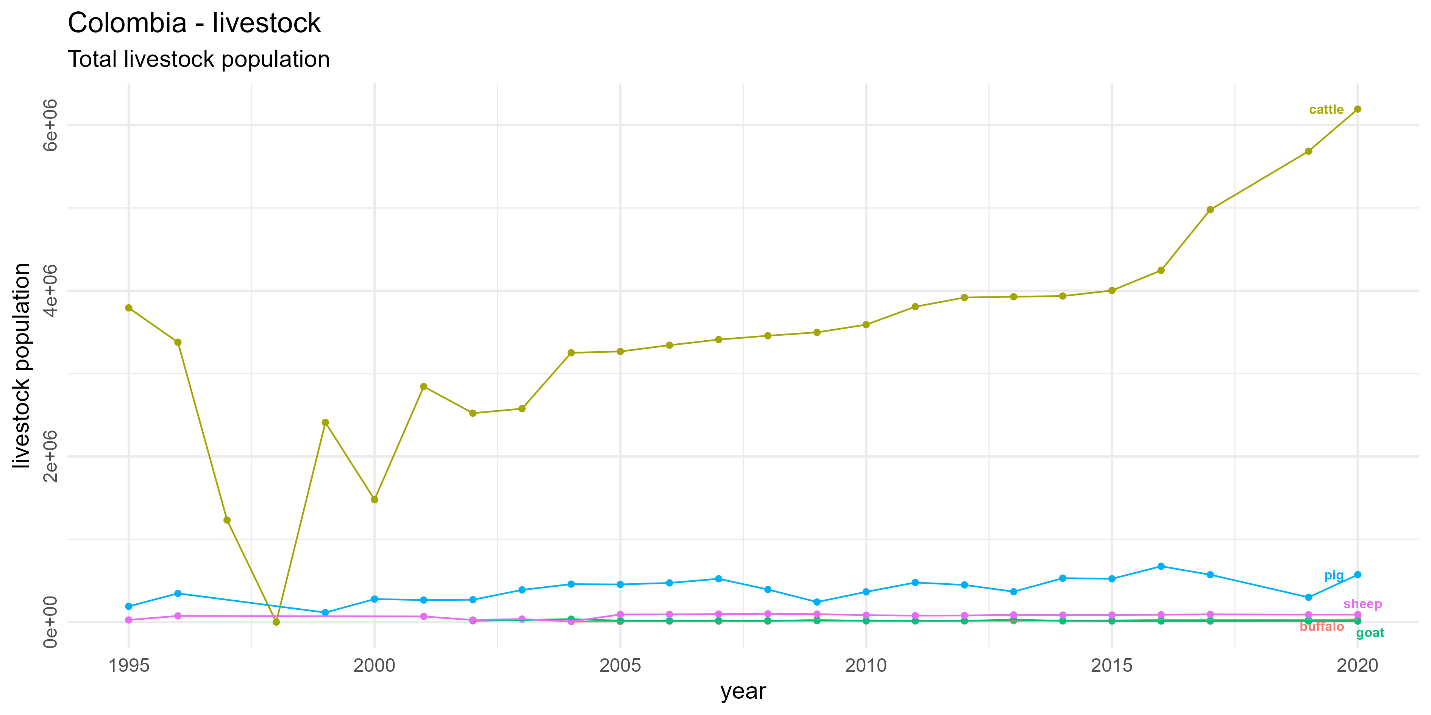


**Figure S20. Livestock in Colombia.** Total livestock population for all major livestock species reported in Colombia for the period 1995-2020.


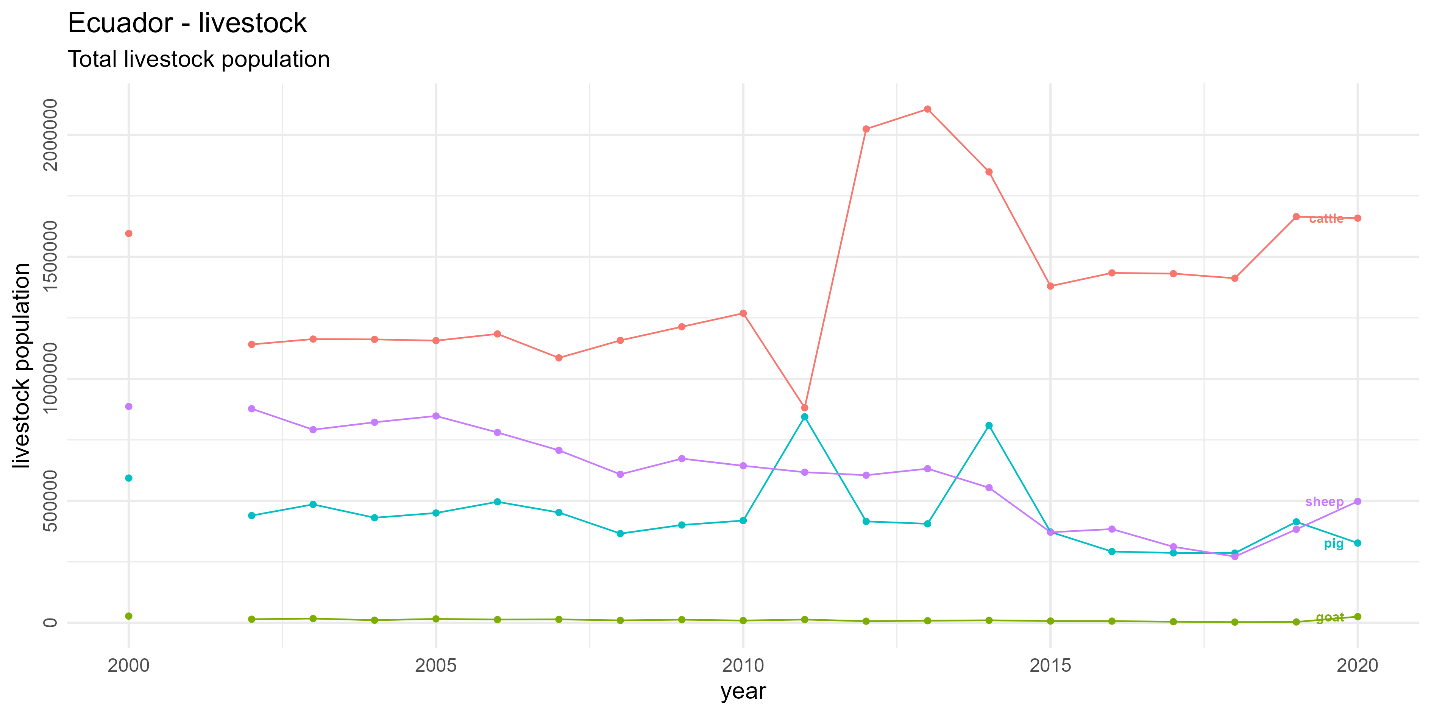


**Figure S21. Livestock in Ecuador.** Total livestock population for all major livestock species reported in Ecuador for the period 2000-2020.


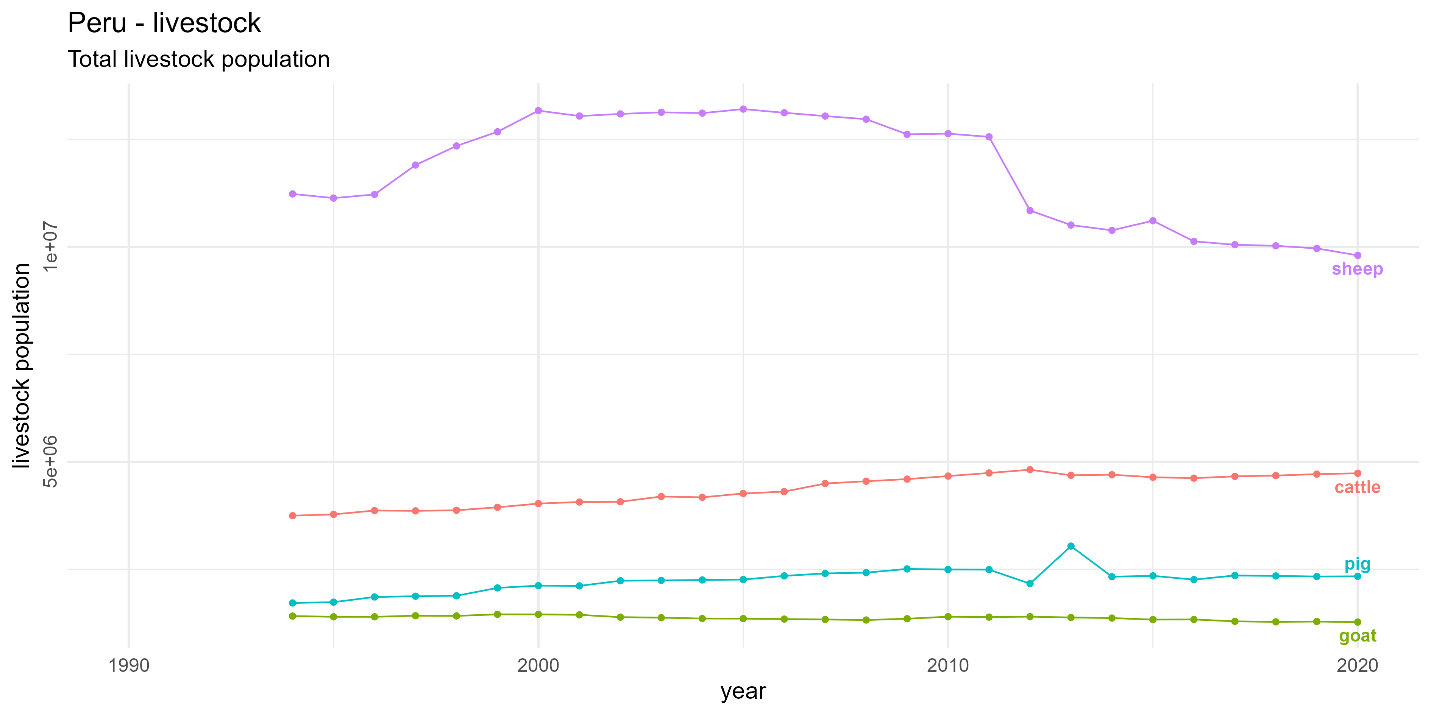


**Figure S22. Livestock in Peru.** Total livestock population for all major livestock species reported in Peru for the period 1994-2020.


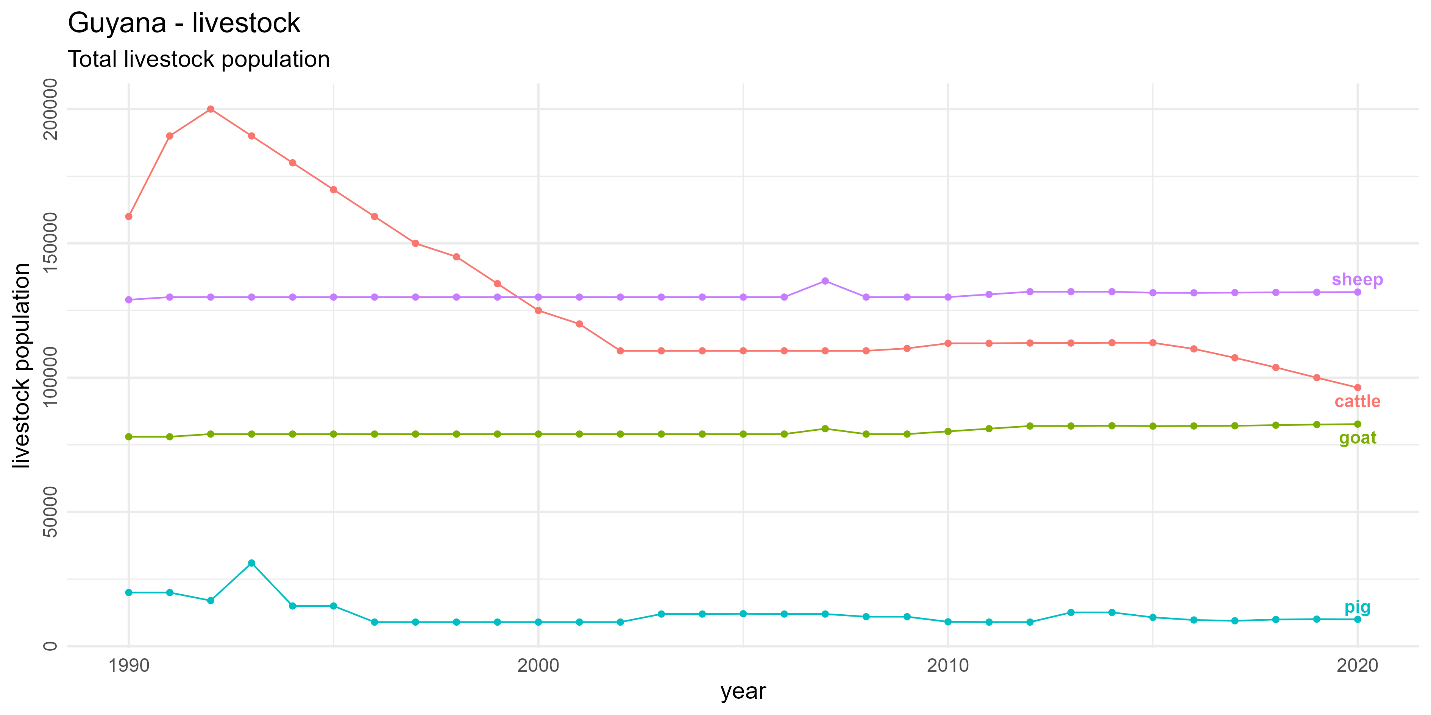


**Figure S23. Livestock in Guyana.** Total livestock population for all major livestock species reported in Guyana for the period 1990-2020**.**


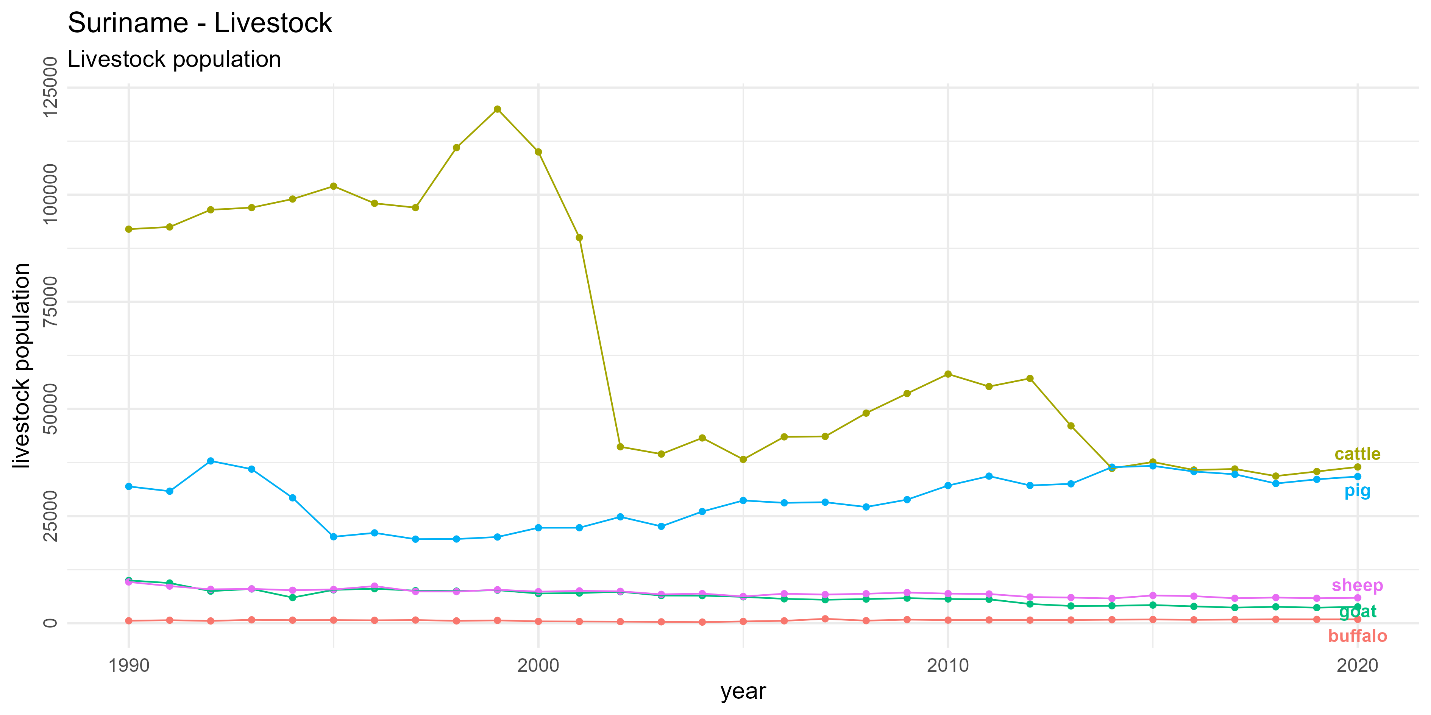


**Figure S24. Livestock in Suriname.** Total livestock population for all major livestock species reported in Suriname for the period 1990-2020.


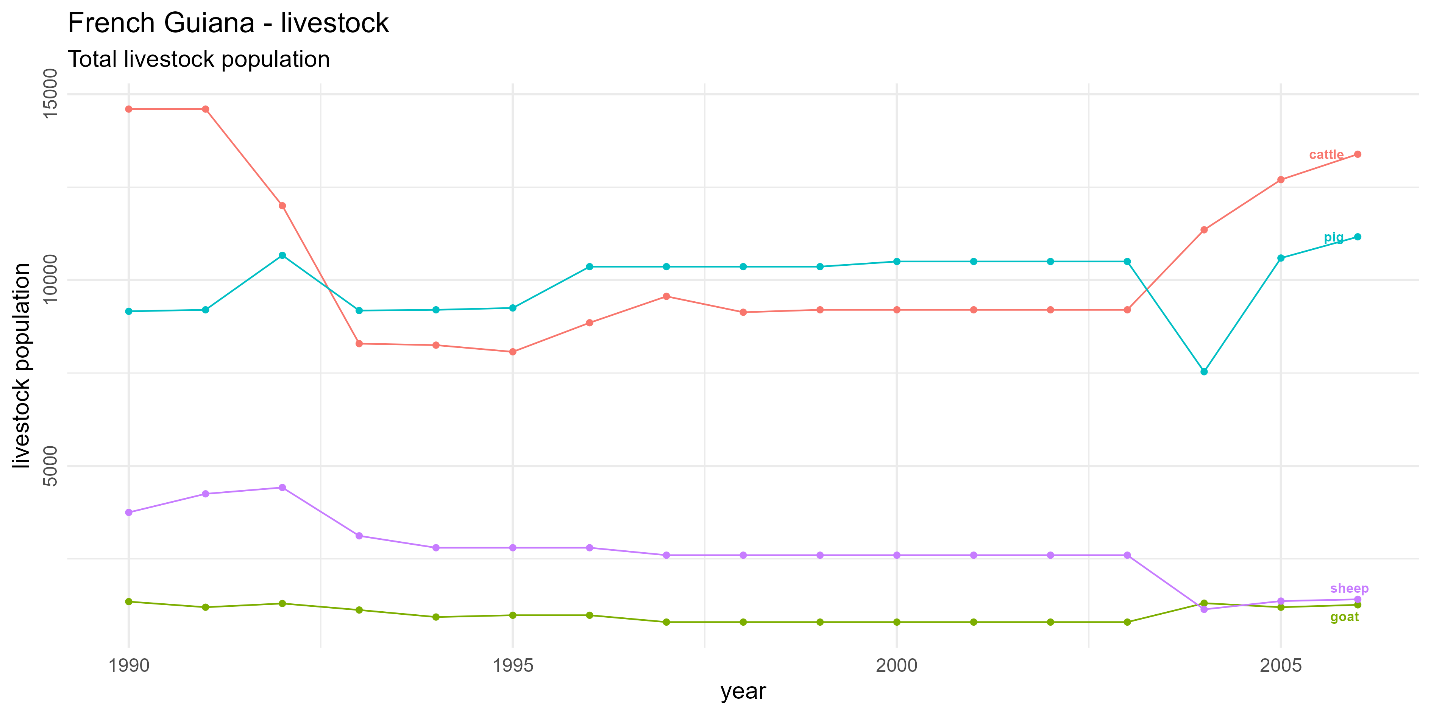


**Figure S25. Livestock in French Guiana.** Total livestock population for all major livestock species reported in French Guiana for the period 1990-2006.

### Mining

| 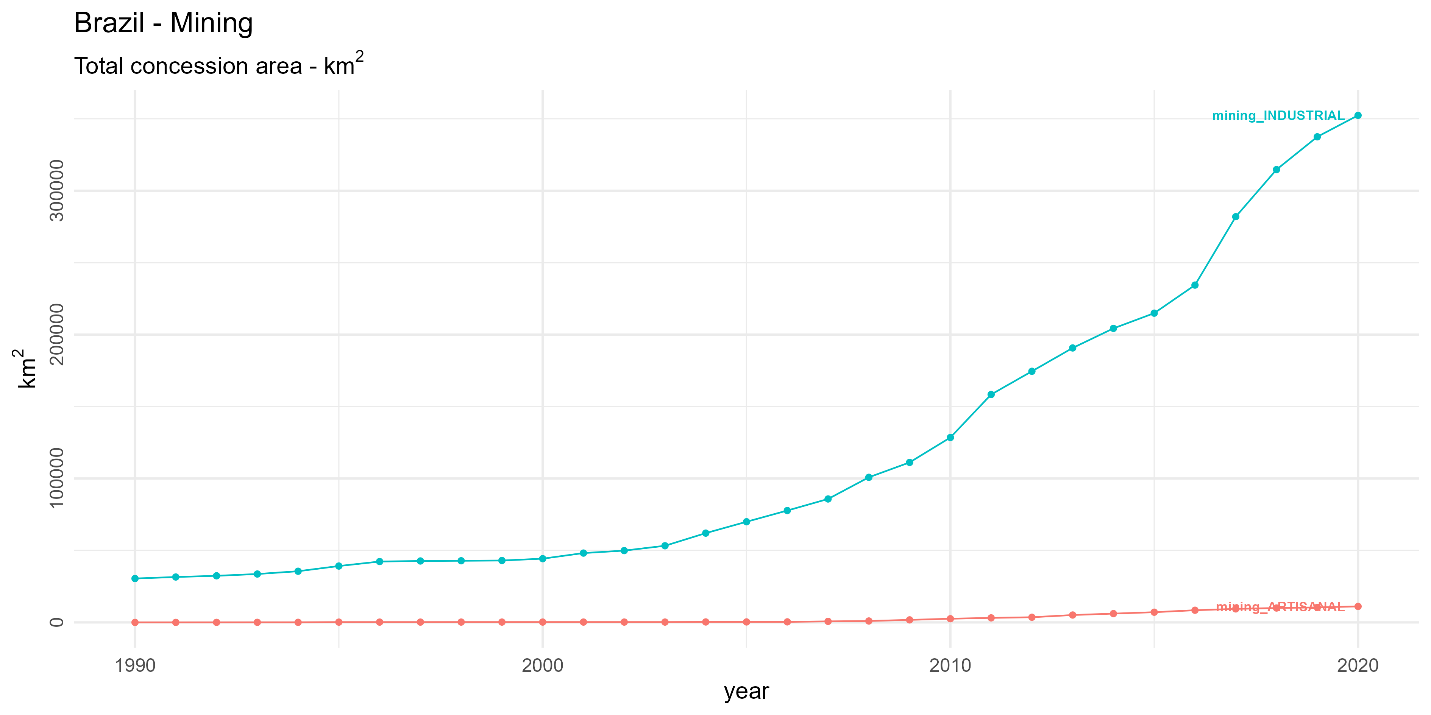 |
| --- |
| 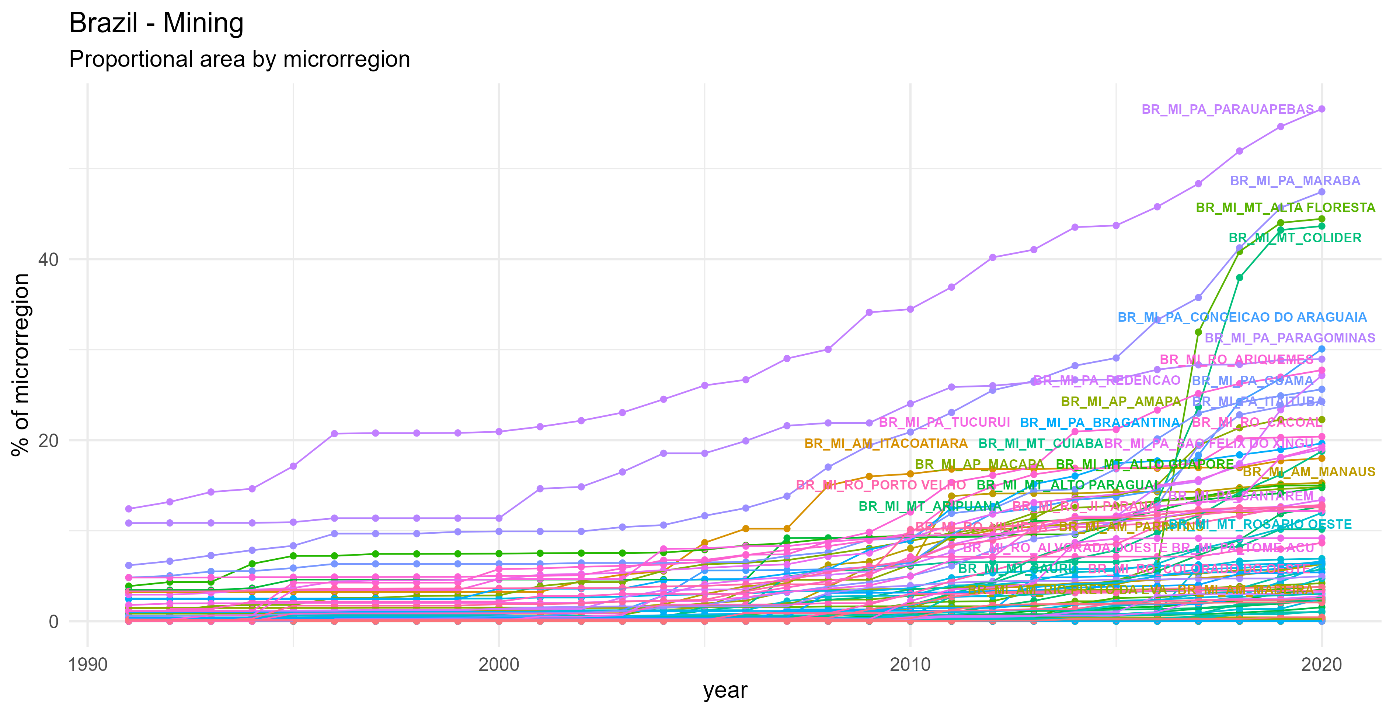 |

**Figure S26. Mining concessions in Brazil.** Total area in mining concessions and permit areas for industrial and artisanal mining, respectively, and proportional area in all categories of mining concessions by microregion for the period 1990-2020.


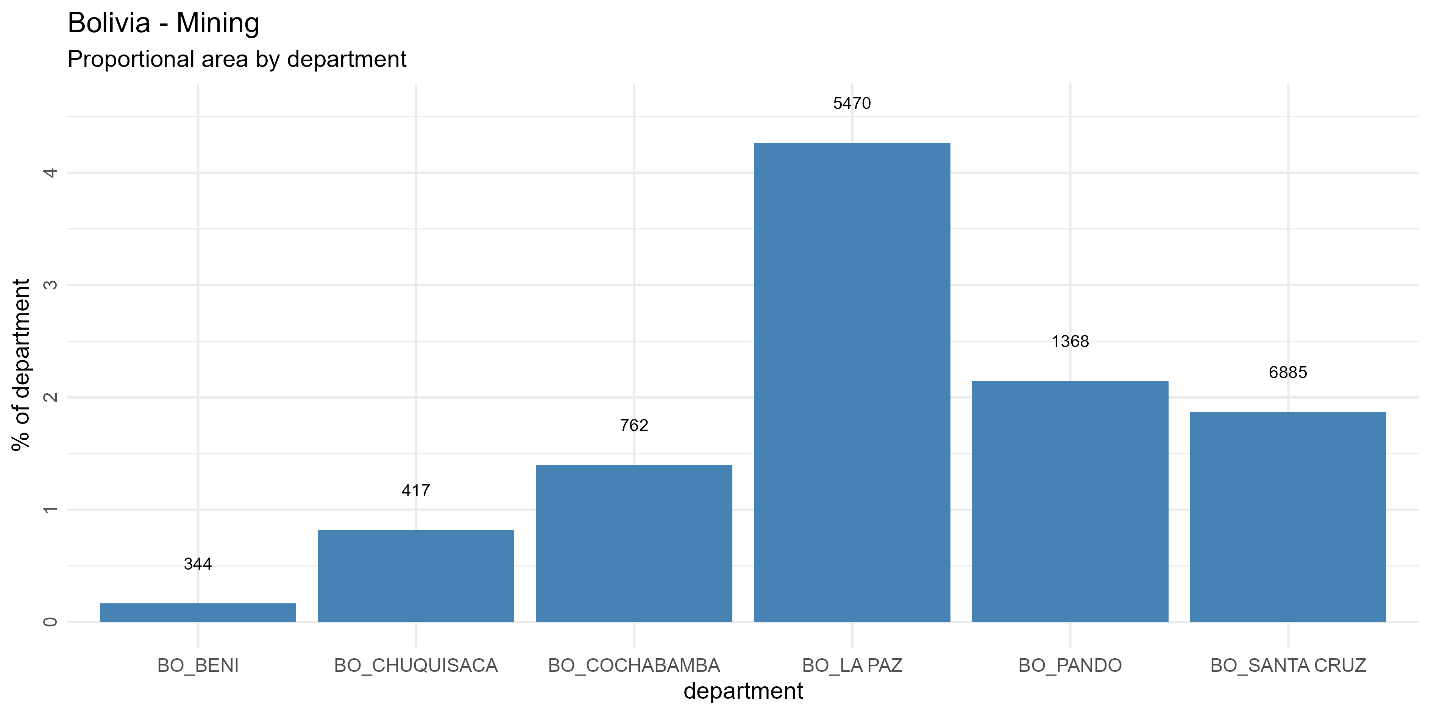


**Figure S 27. Mining concessions in Bolivia.** Proportional area in mining concessions by department in Bolivia for the year 2020. Numbers at the top of each bar correspond to the total concessions area in km^2^.

| 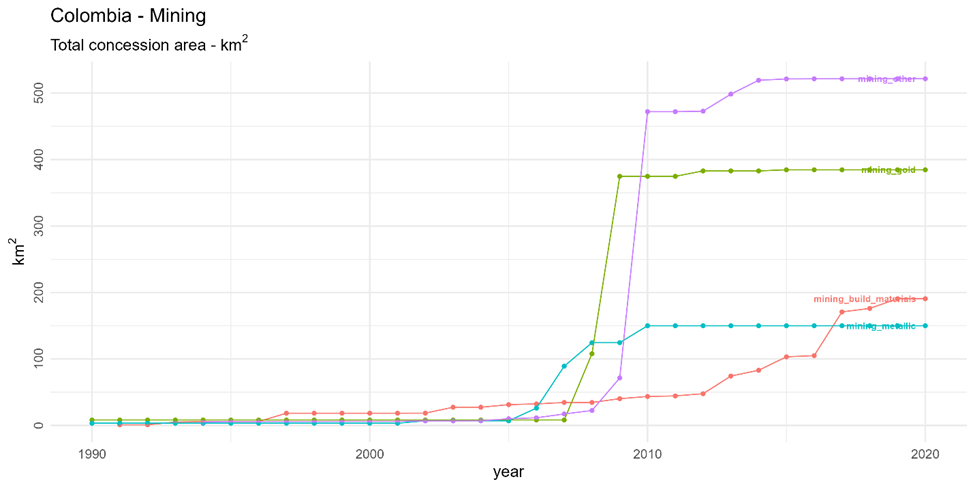 |
| --- |
| 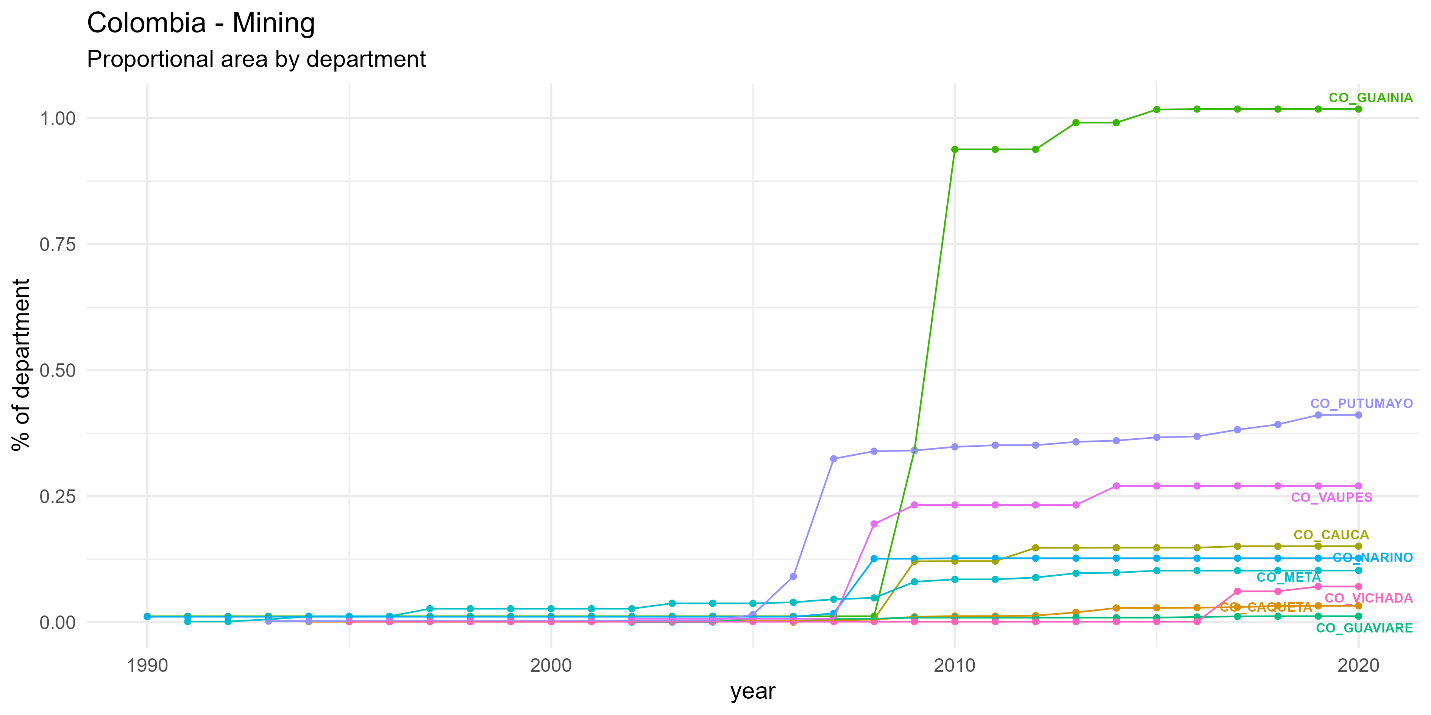 |

**Figure S28. Mining concessions in Colombia.** Total area in mining concessions and permit areas by mineral type, and proportional area in all categories of mining concessions by department for the period 1990-2020.

| 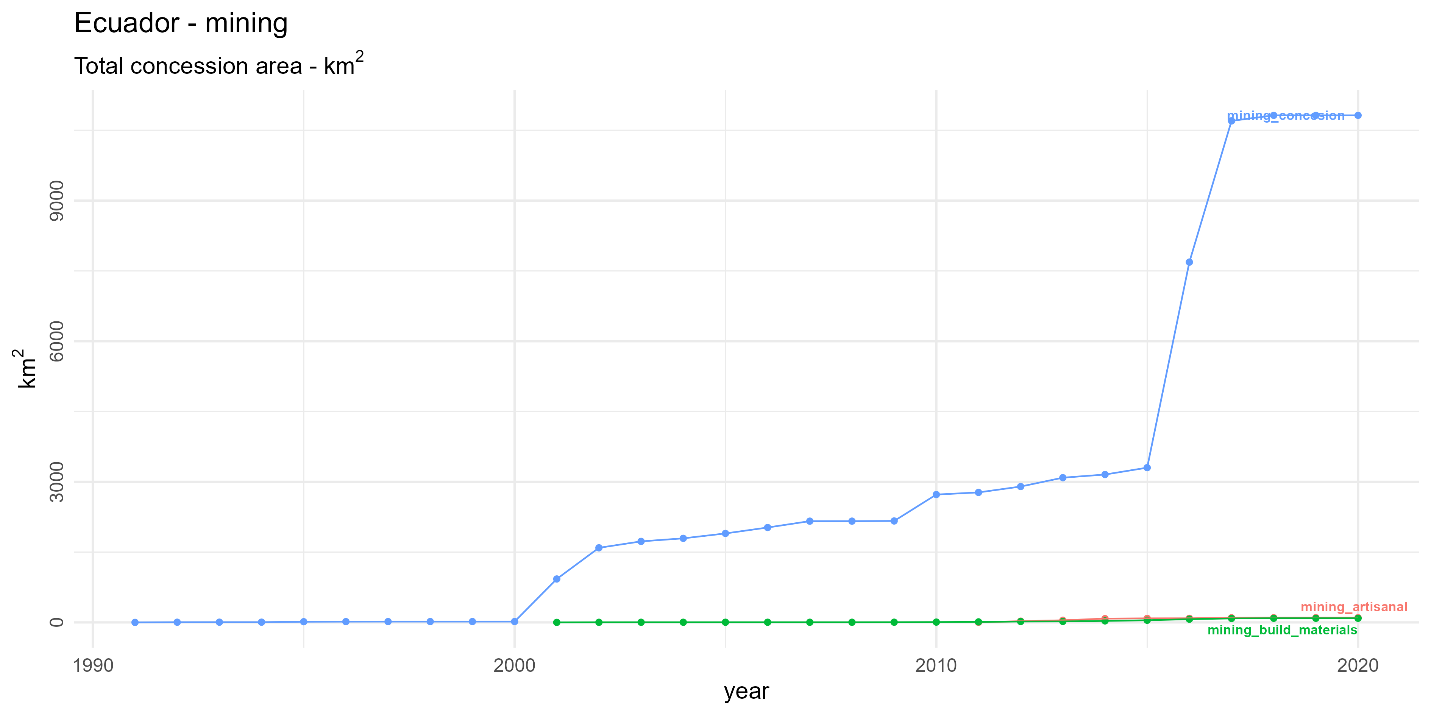 |
| --- |
| 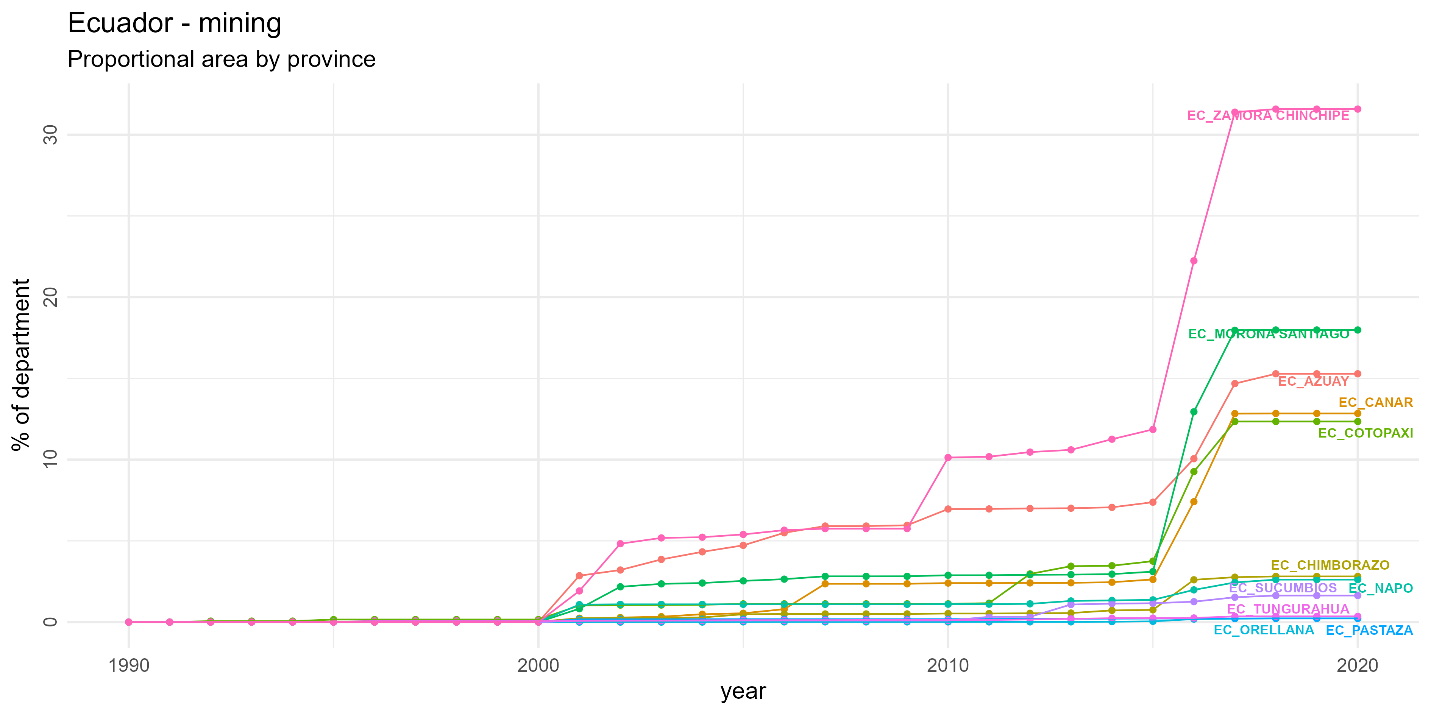 |

**Figure S29. Mining concessions in Ecuador.** Total area in mining concessions and permit areas for industrial, artisanal and building materials mining, respectively, and total area in all categories, by province for the period 1990-2020.

| 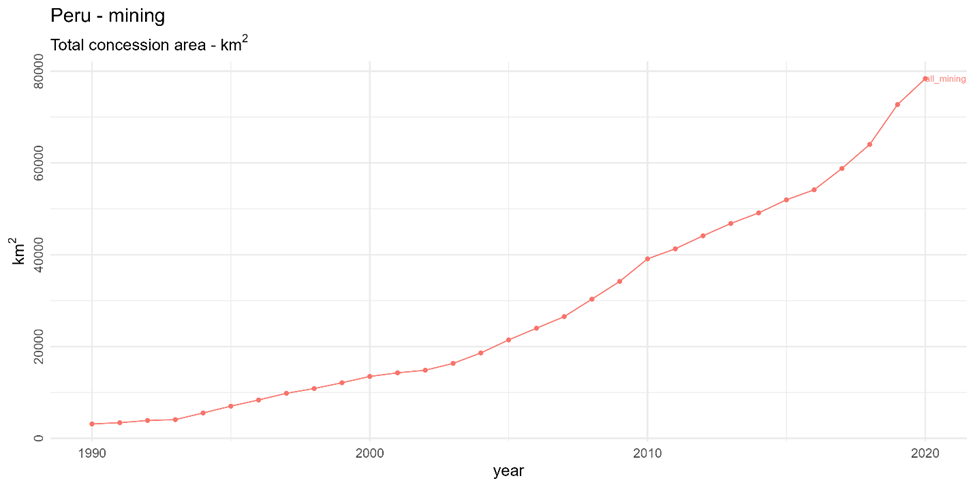 |
| --- |
| 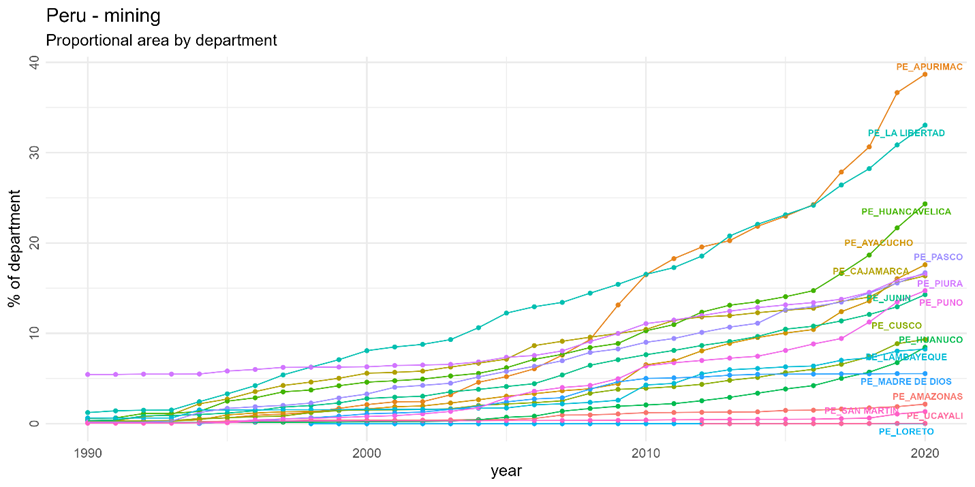 |

**Figure S30. Mining concessions in Peru.** Total area in mining concessions and proportional area by department for the period 1990-2020.

| 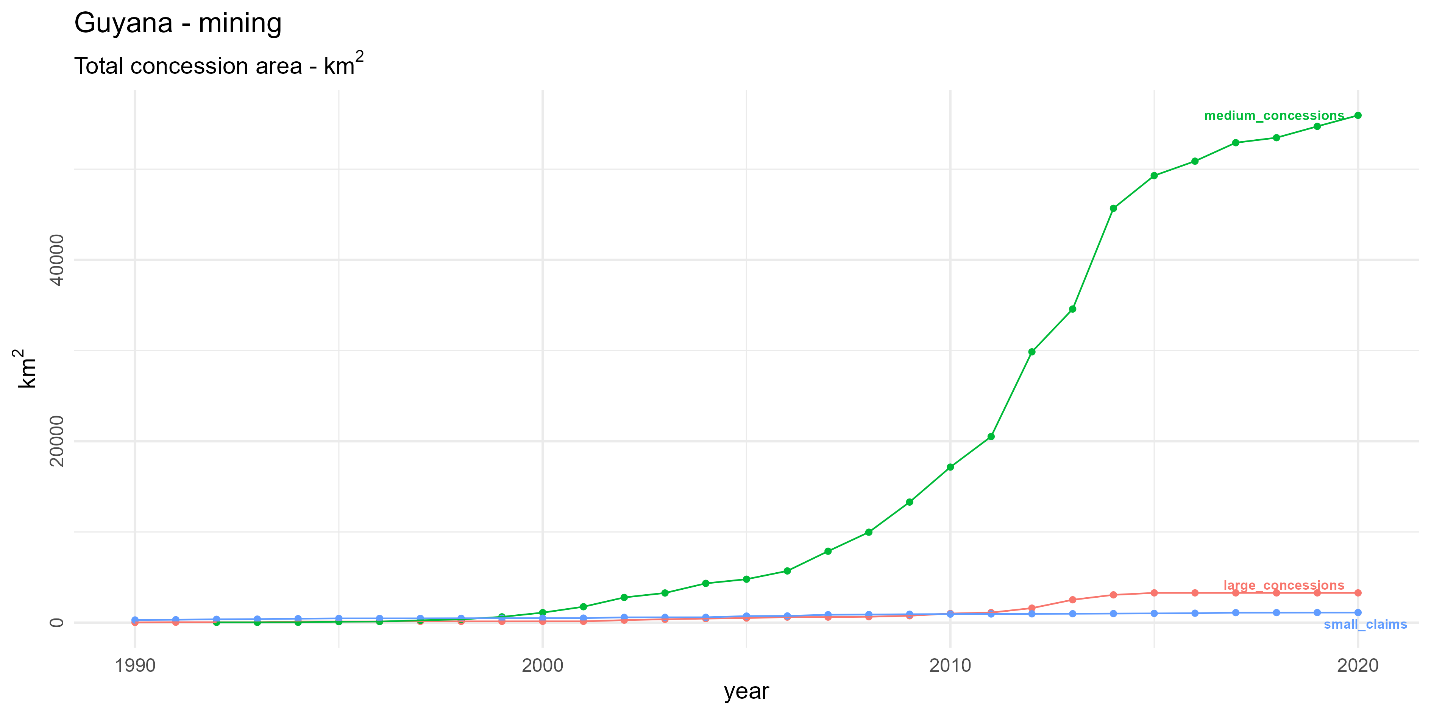 |
| --- |
| 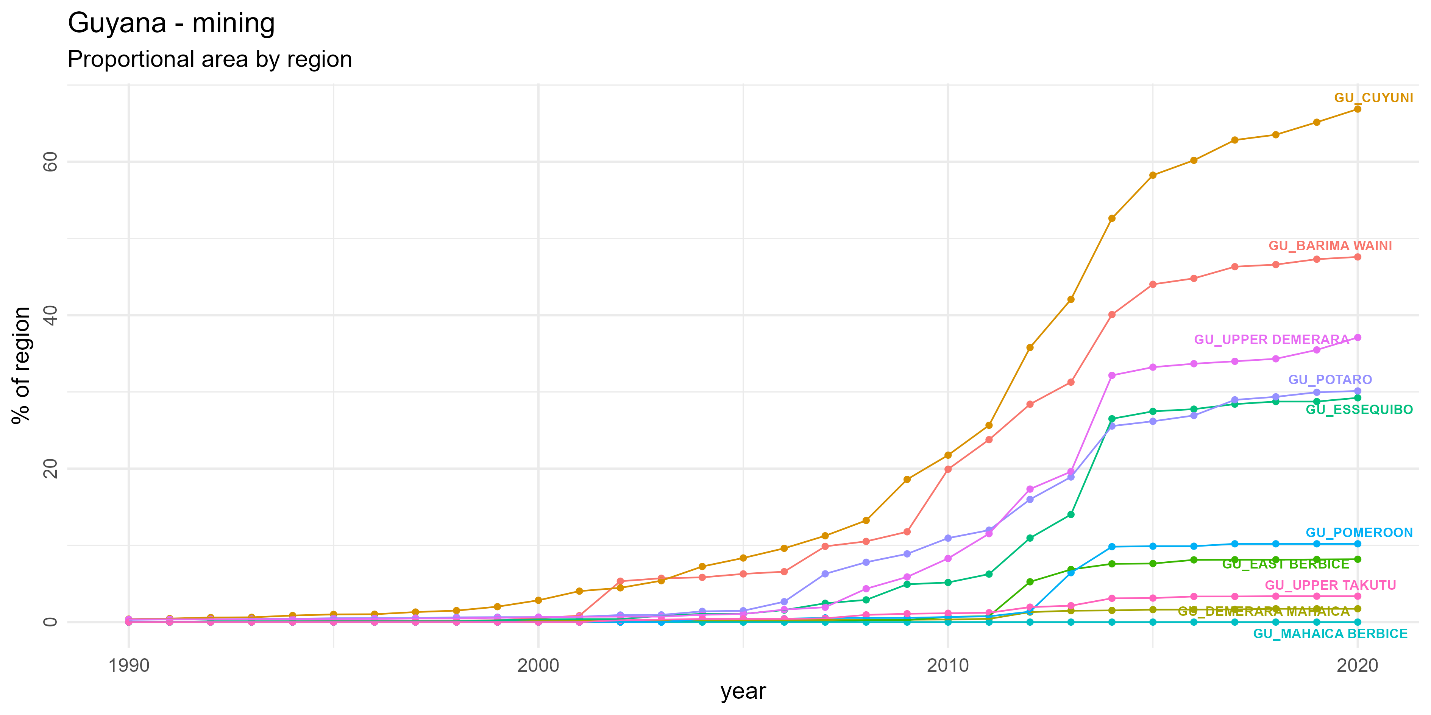 |

**Figure S31. Mining concessions in Guyana.** Total area in mining concessions by size of concession, and proportional area for all categories by region, for the period 1990-2020.

| 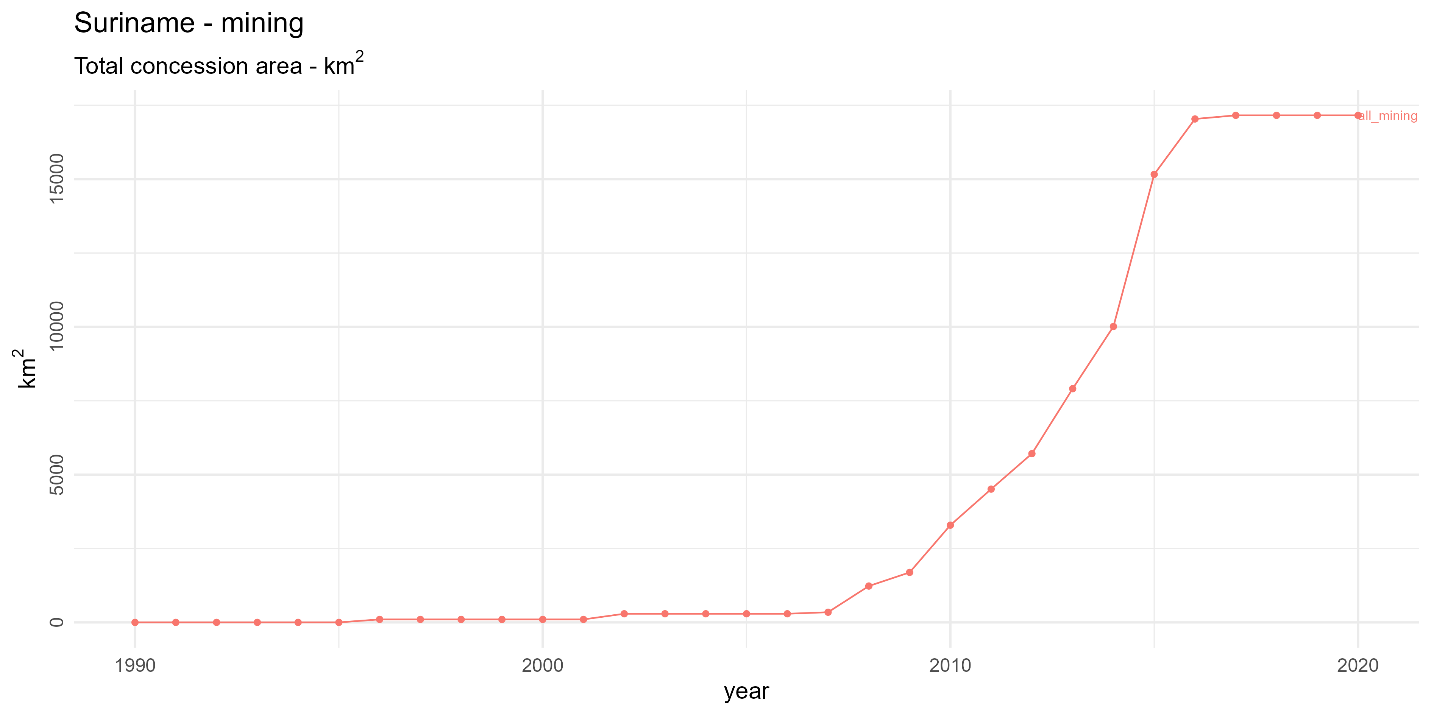 |
| --- |
| 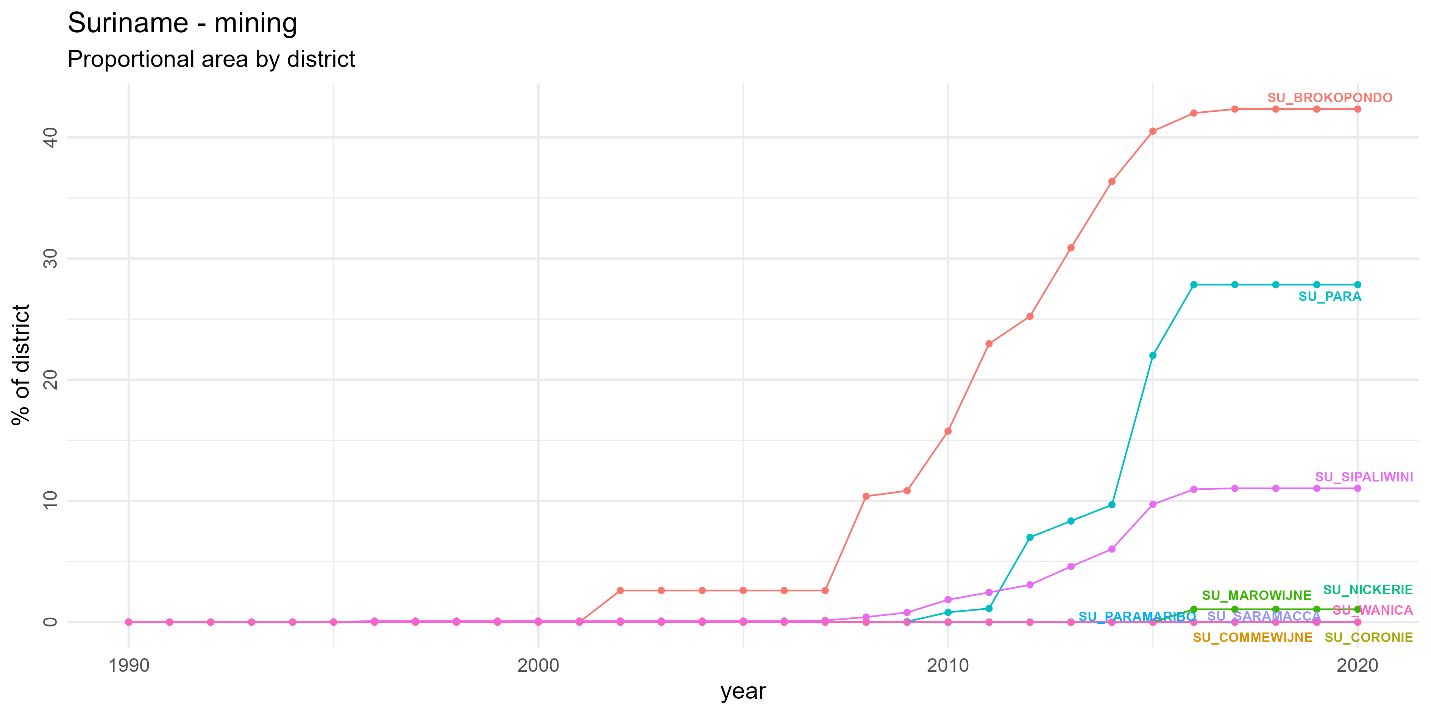 |

**Figure S32. Mining concessions in Suriname.** Total area in mining concessions and proportional area by district for the period 1990-2020.


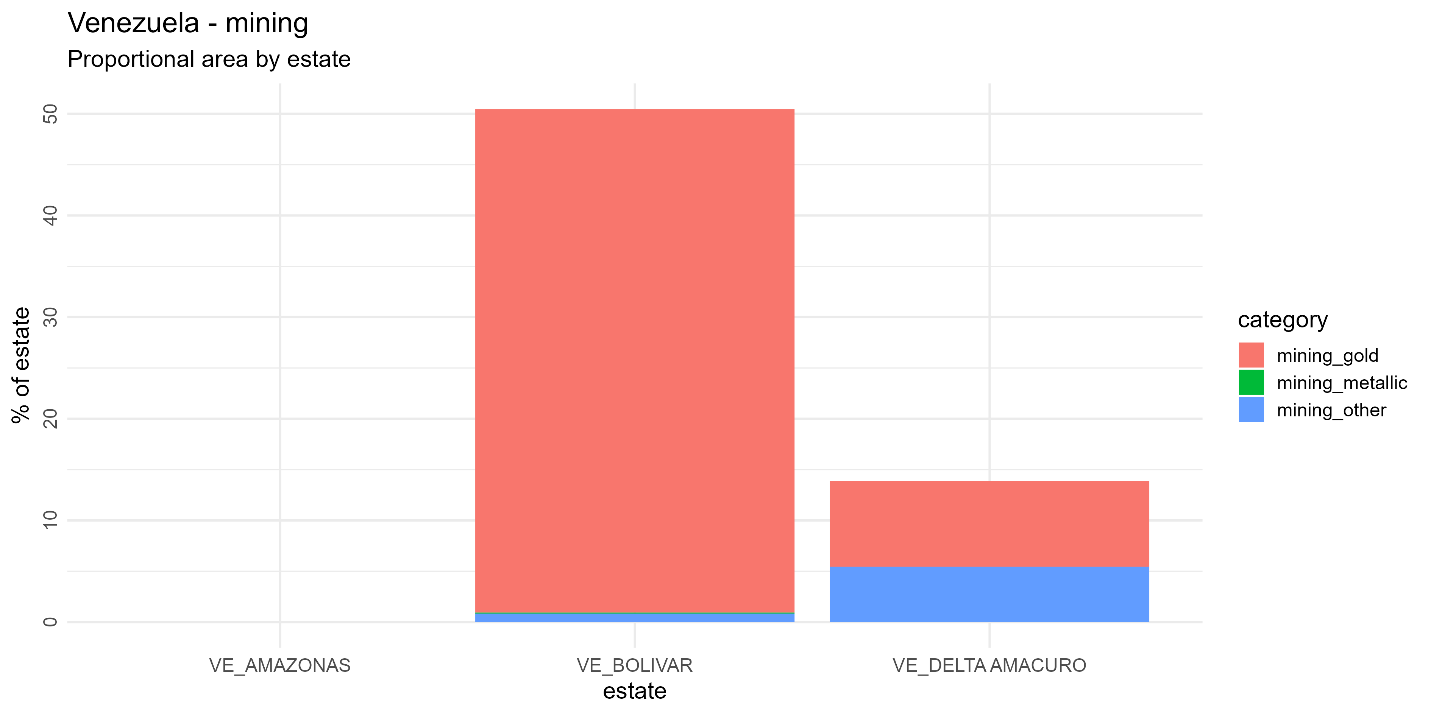


**Figure S33. Mining concessions in Venezuela.** Total area in mining concessions and proportional area by estate for the year 2020.

### Oil

| 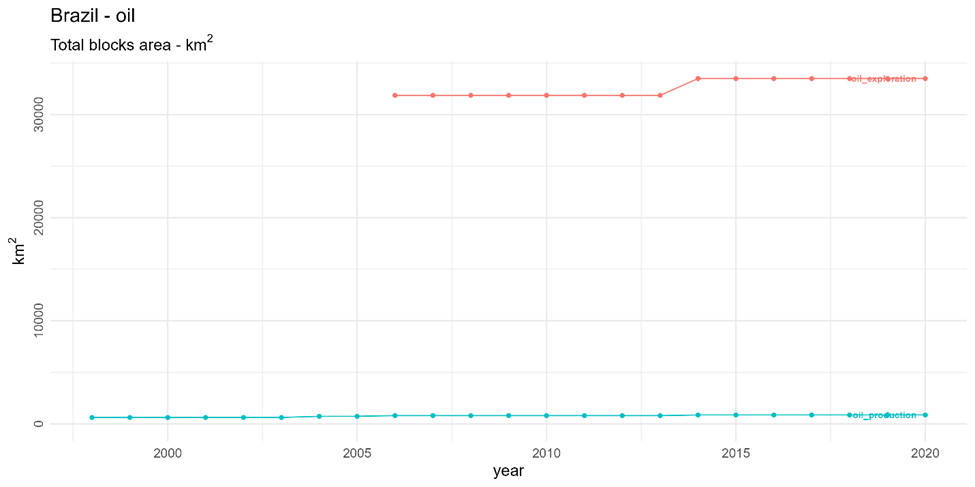 |
| --- |
| 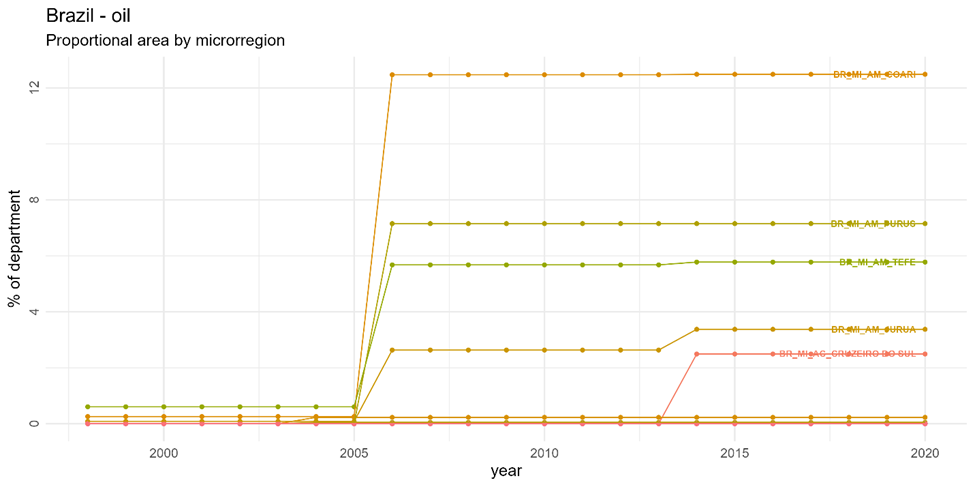 |

**Figure S34. Oil blocks in Brazil.** Above: total area in oil blocks by type of lot; below: proportional area in oil blocks by microregion in Brazil for the period 1998-2020.

| 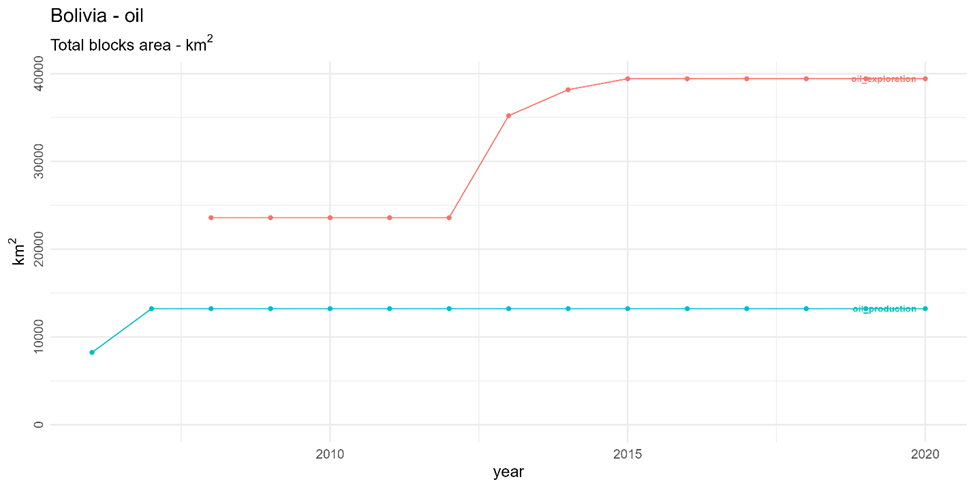 |
| --- |
| 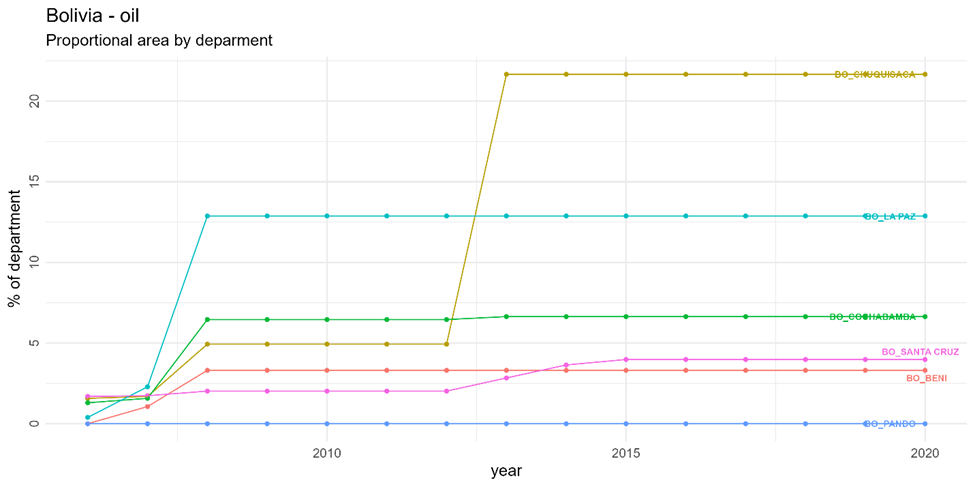 |

**Figure S35. Oil blocks in Bolivia.** Above: total area in oil blocks by type of lot; below: total area in oil blocks by department in Bolivia for the period 2006-2020.

| 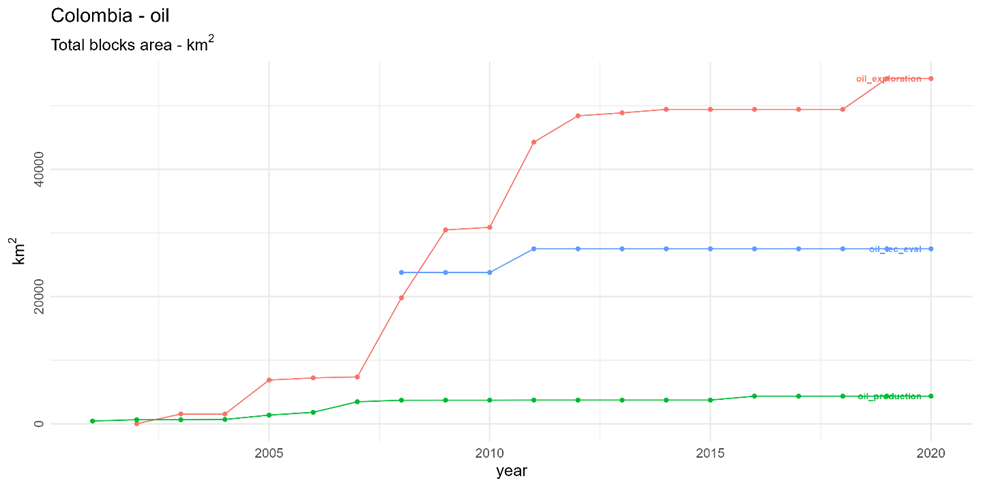 |
| --- |
| 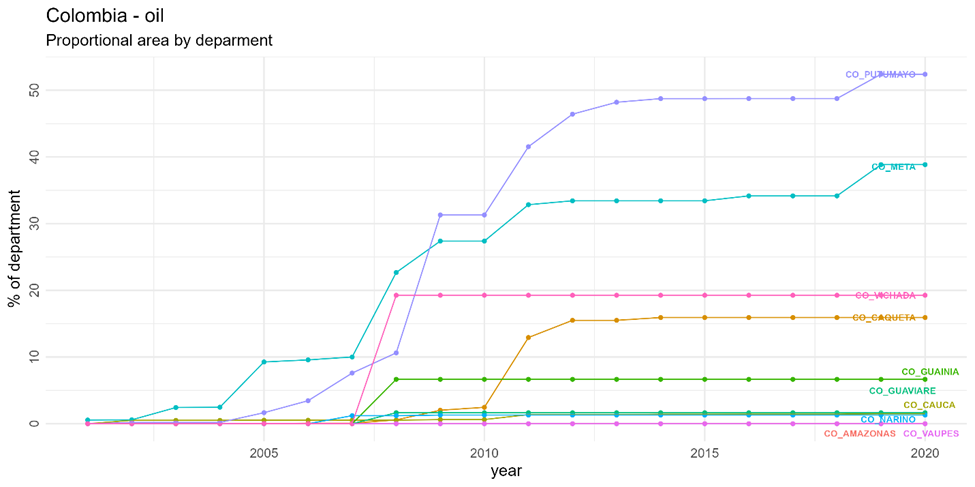 |

**Figure S36. Oil blocks in Colombia.** Above: total area in oil blocks by type of lot; below: total area in oil blocks by department in Colombia for the period 2001-2020.


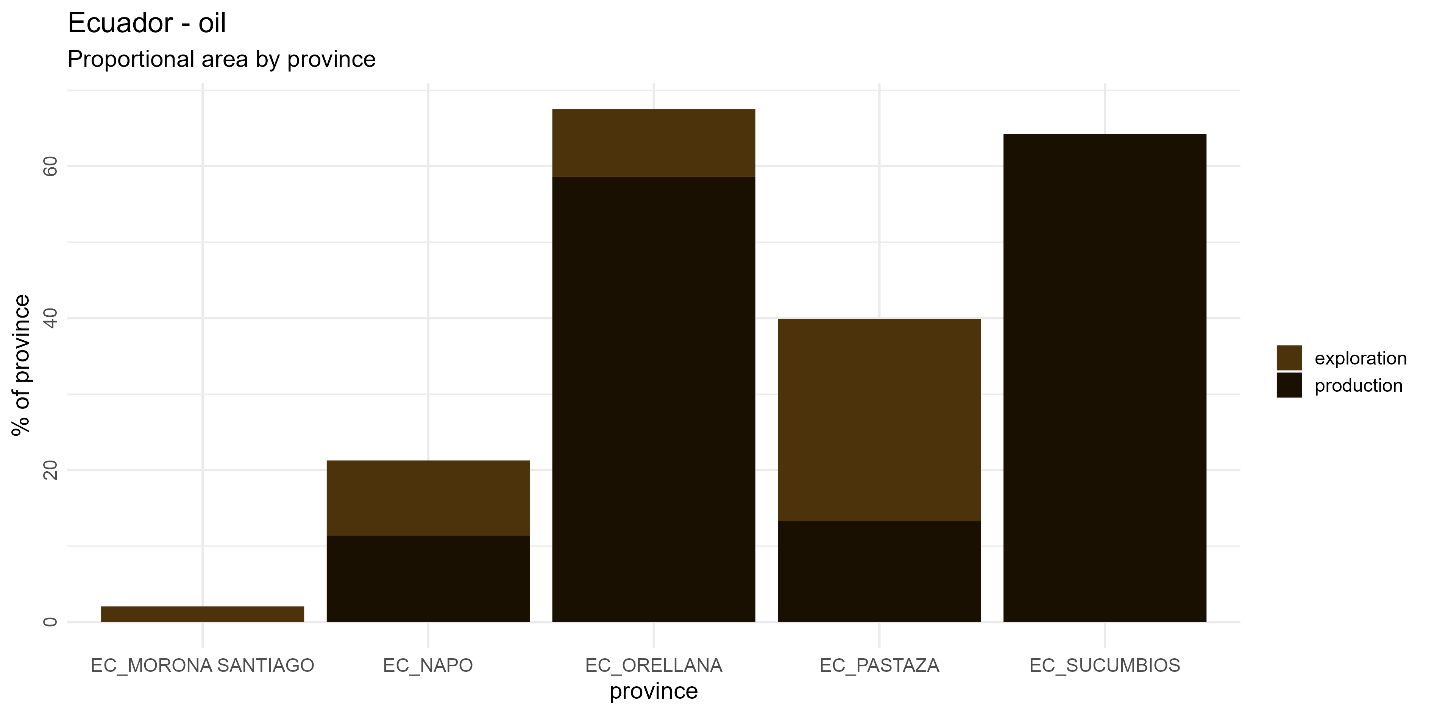


**Figure SB.37. Oil blocks in Ecuador.** Proportional area of different type of oil blocks by province, in Ecuador for the year 2017.

| 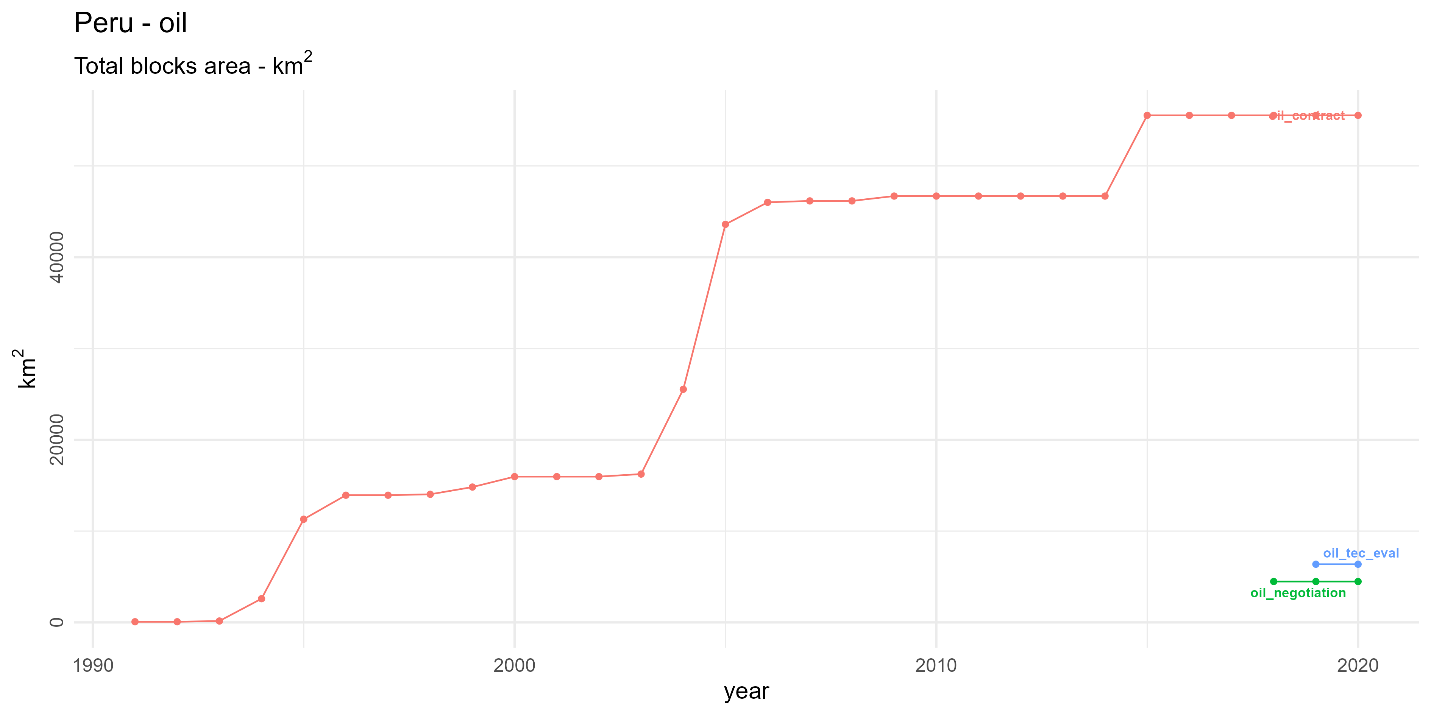 |
| --- |
| 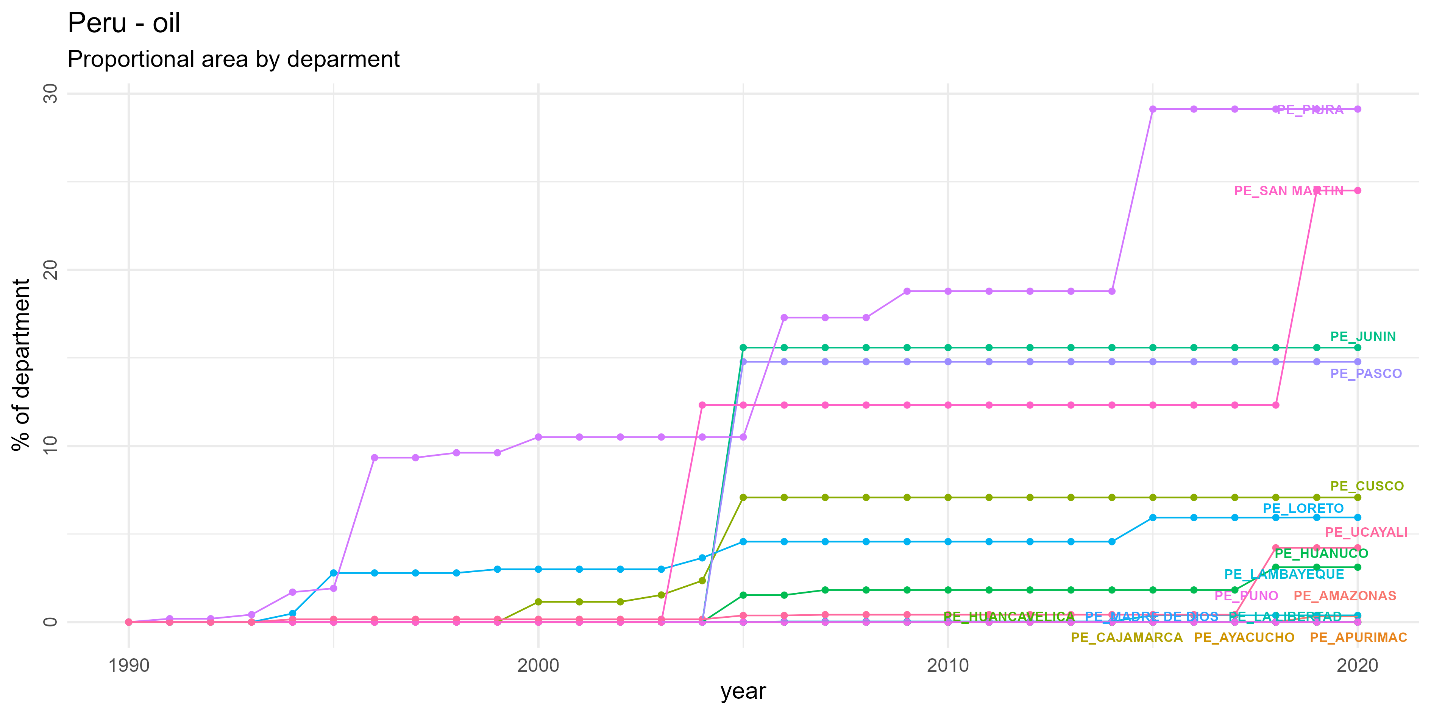 |

**Figure S37. Oil blocks in Peru.** Above: total area in oil blocks by type of lot; below: total area in oil blocks by department in Peru for the period 1990-2020.


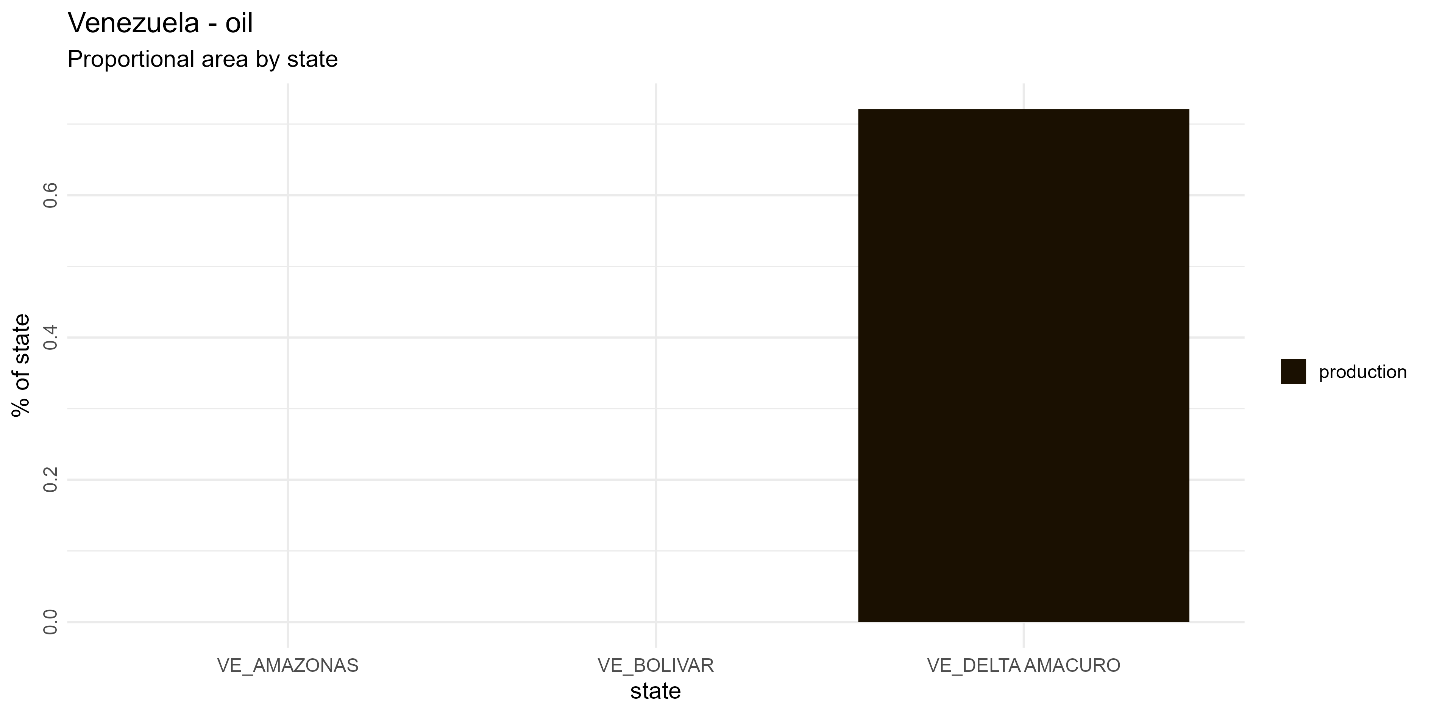


**Figure S38. Oil blocks in Venezuela.** Proportional area of different type of oil blocks by state, in Venezuela for the year 2019.

### Roads

| 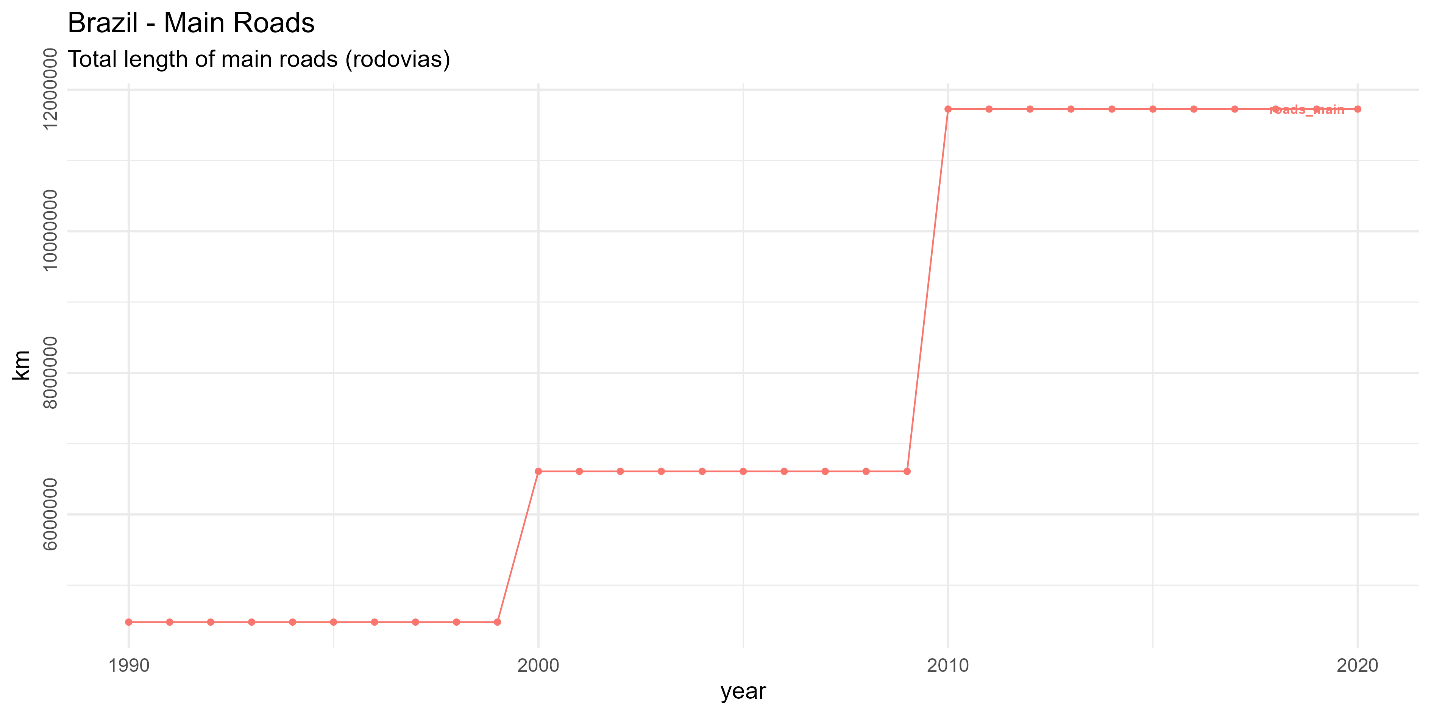 |
| --- |
| 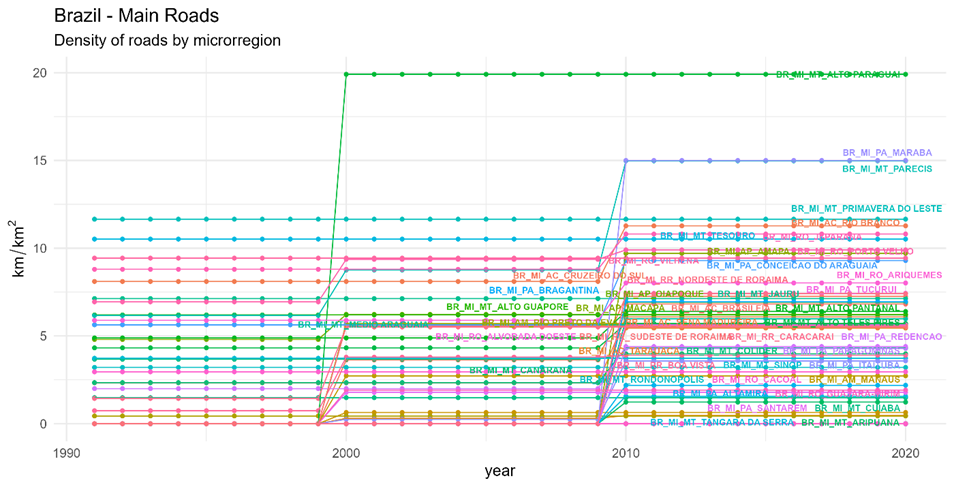 |

**Figure S39. Main roads extension in Brazil.** Above, total extension of main roads in Brazil for the period 1990-2020. Below, main roads density by microregion.

| 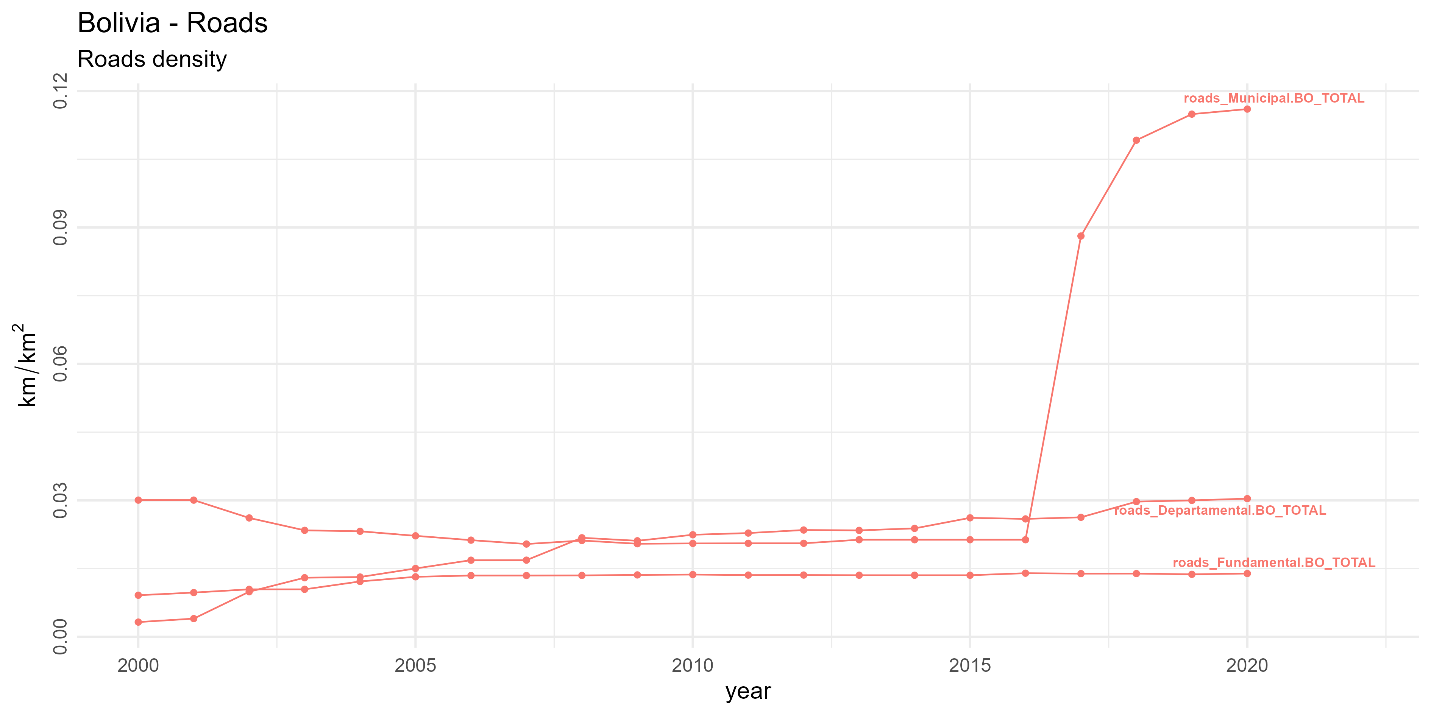 |
| --- |
| 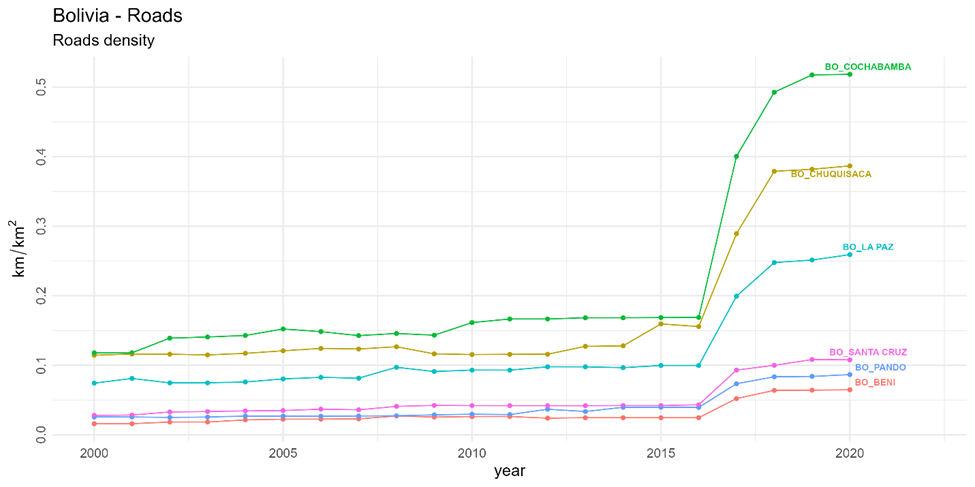 |

**Figure S40. Roads extension in Bolivia.** Above, total roads extension for fundamental, departmental and municipal roads for the period 1990-2020. Below, density of total roads (roads km/ department km^2^) by department.

### Protected Areas

| 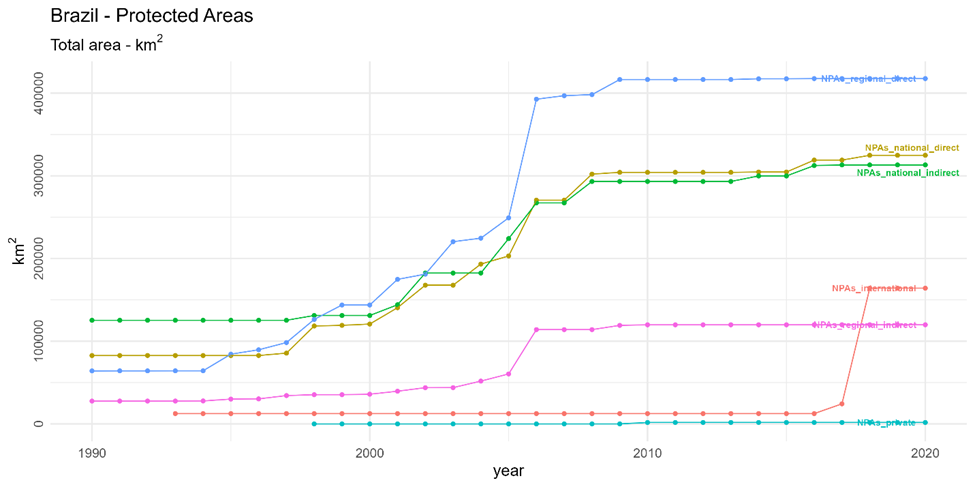 |
| --- |
| 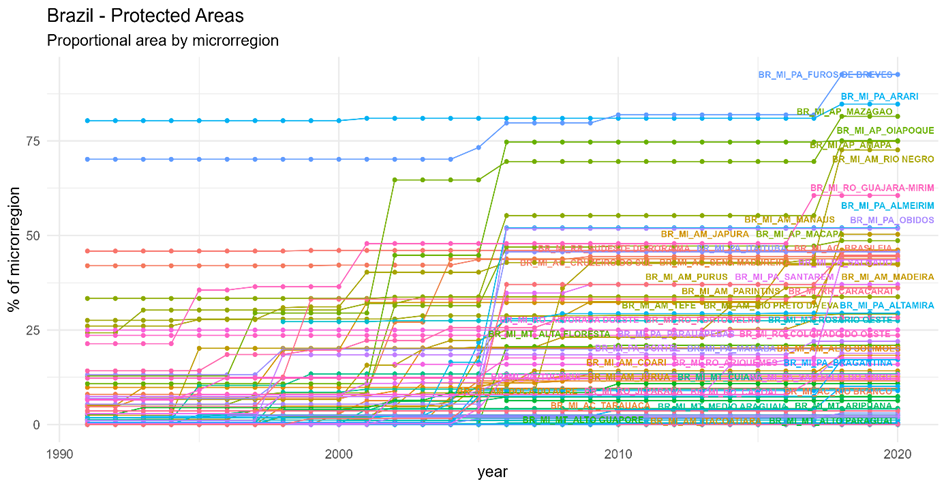 |

**Figure S41. Protected Areas in Brazil.** Above, total area in Protected Areas in Brazil by the management categories we defined for the period 1990-2020. Below, proportional area of microregions in all Protected Areas.

| 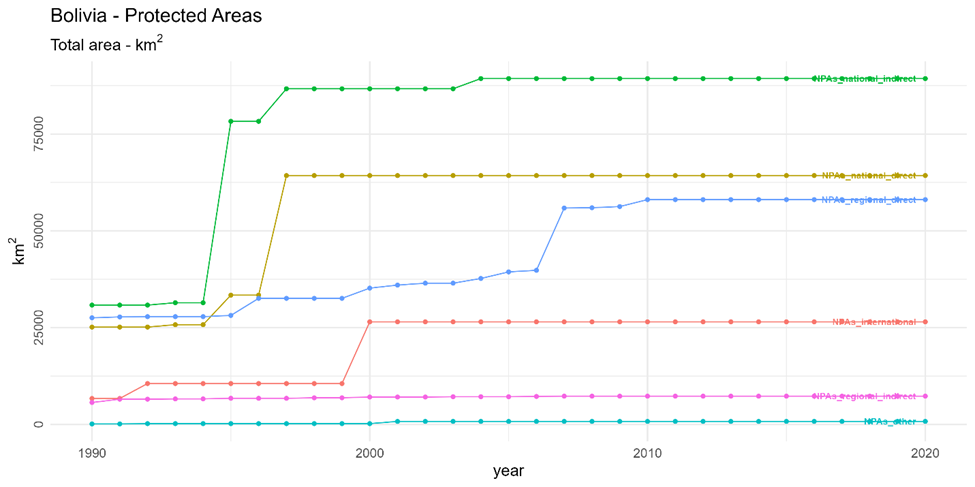 |
| --- |
| 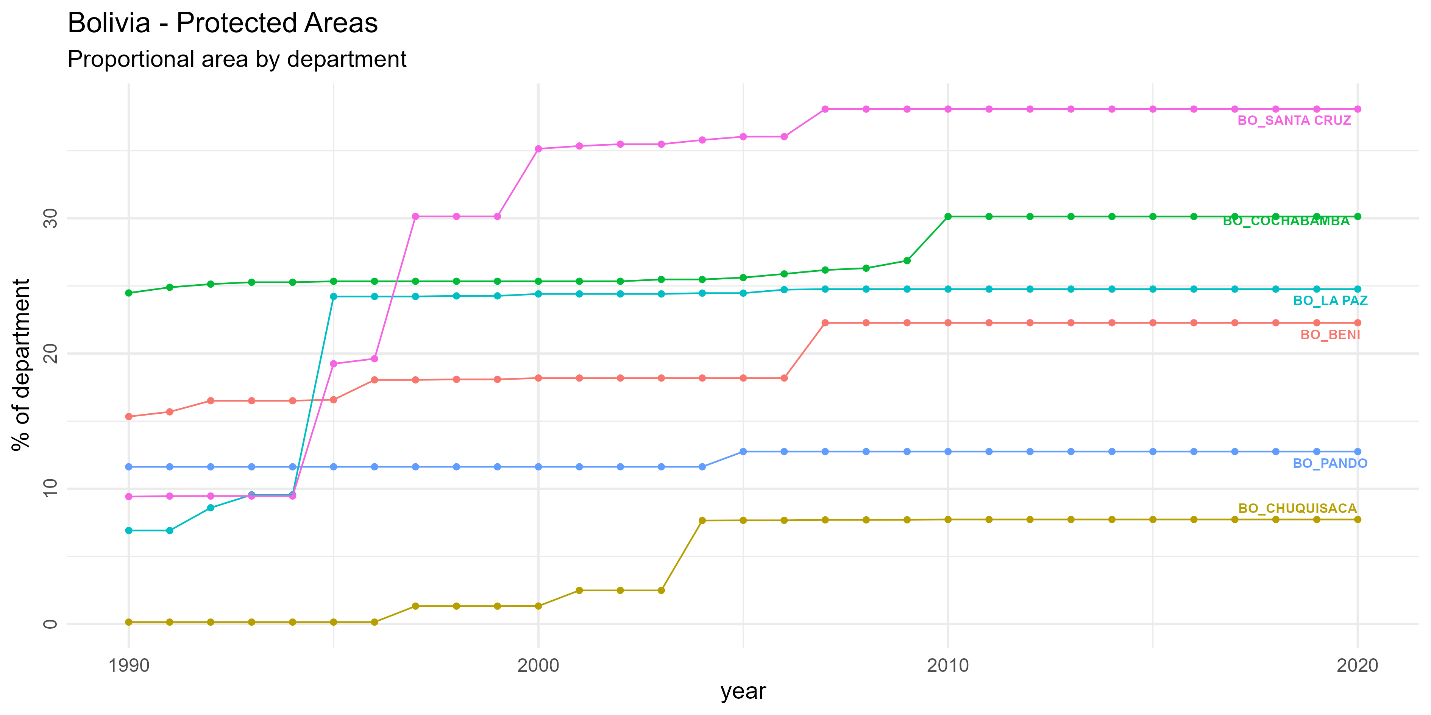 |

**Figure S42. Protected areas in Bolivia.** Above, total area in Protected Areas in Bolivia by the management categories we defined for the period 1990-2020. Below, proportional area of departments in all Protected Areas.

| 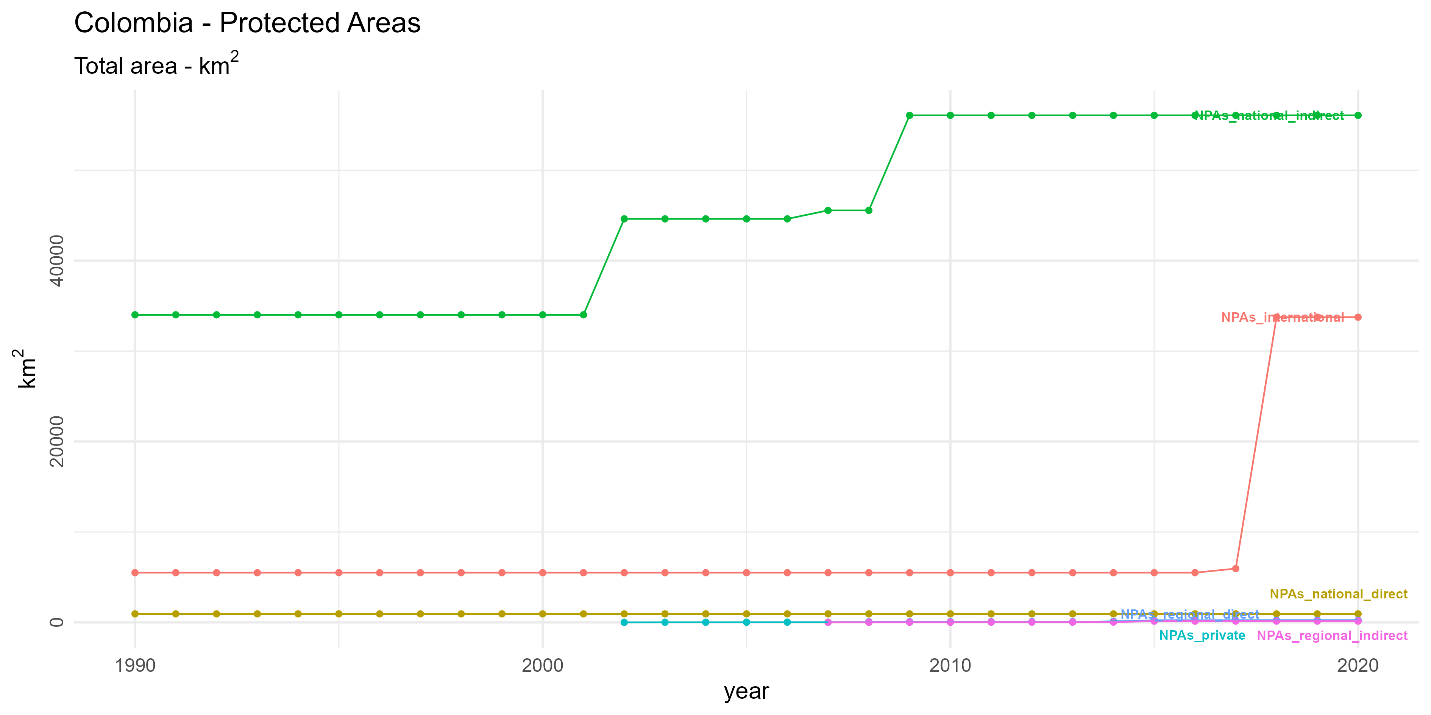 |
| --- |
| 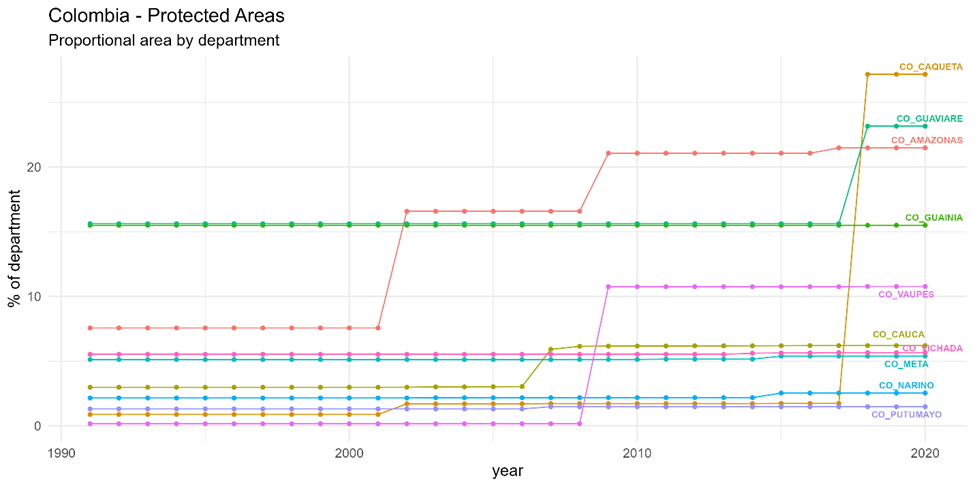 |

**Figure S43. Protected areas in Colombia.** Above, total area in Protected Areas in Colombia by the management categories we defined for the period 1990-2020. Below, total area for all categories by department.

| 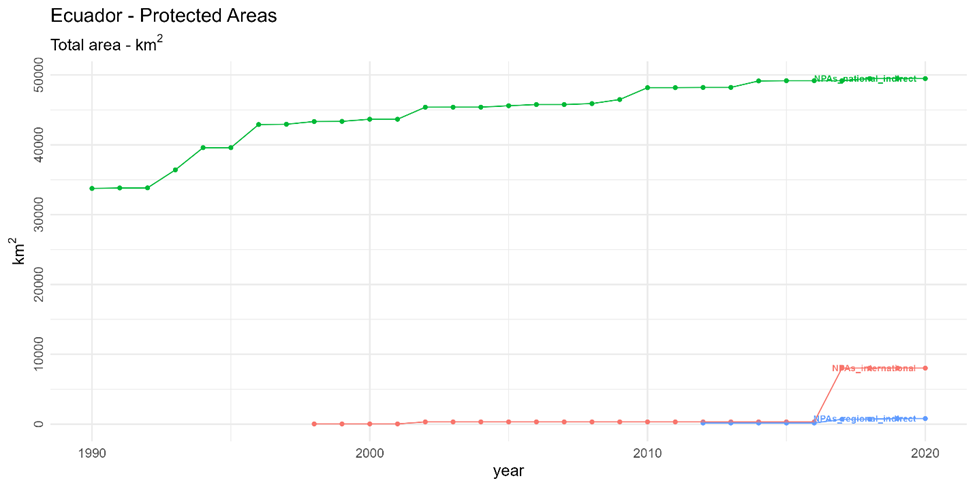 |
| --- |
| 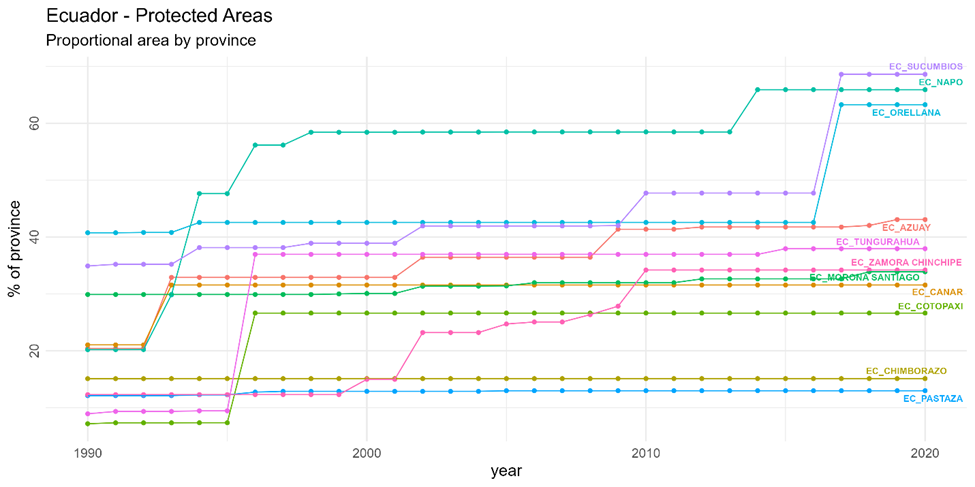 |

**Figure S44. Protected areas in Ecuador.** Above, total area in Protected Areas in Colombia by the management categories we defined for the period 1990-2020. Below, total area for all categories by province.

| 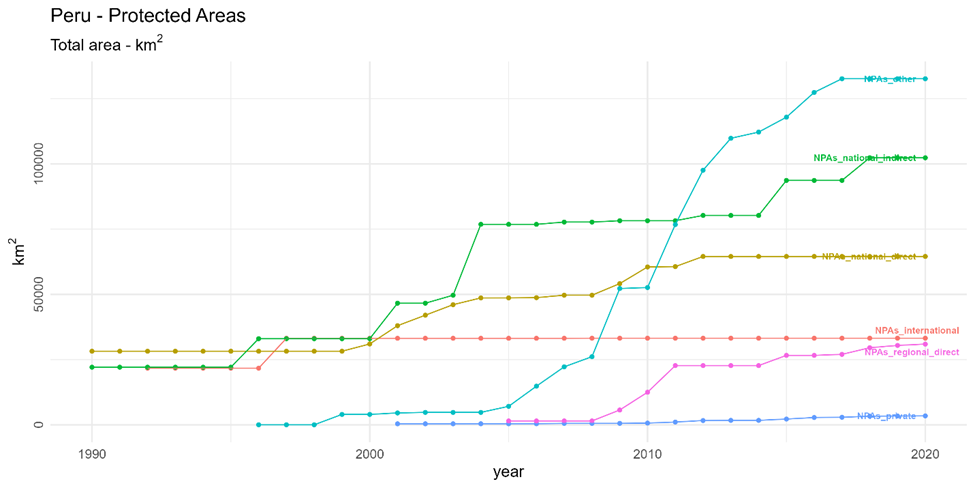 |
| --- |
| 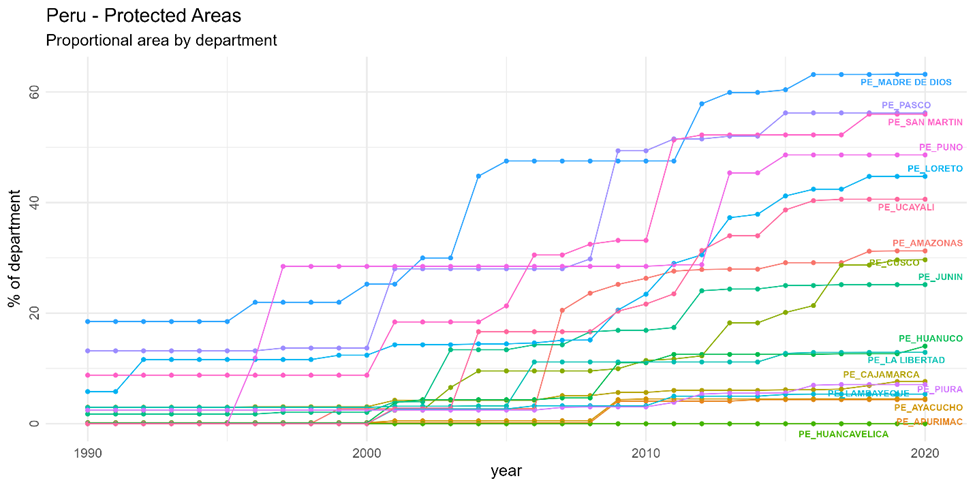 |

**Figure S45. Protected areas in Peru.** Above, total area in Protected Areas in Peru by the management categories we defined for the period 1990-2020. Below, total area for all categories by department.

| 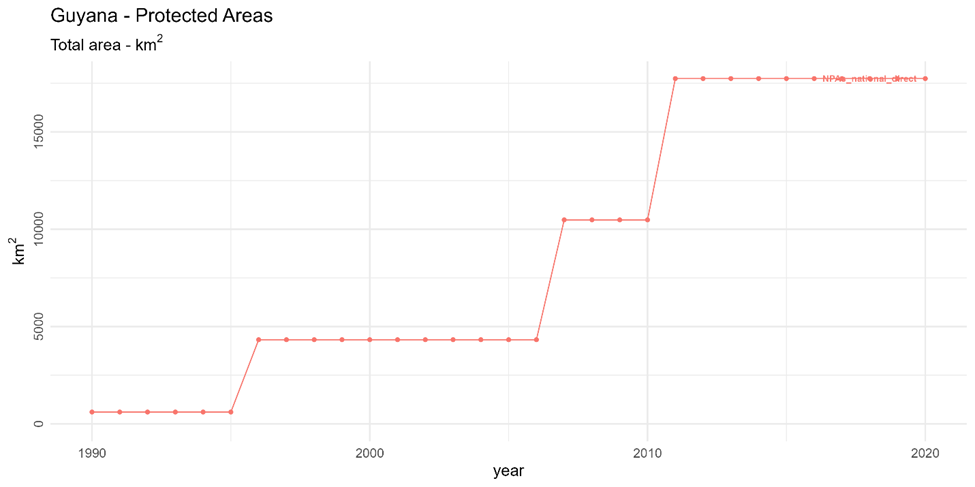 |
| --- |
| 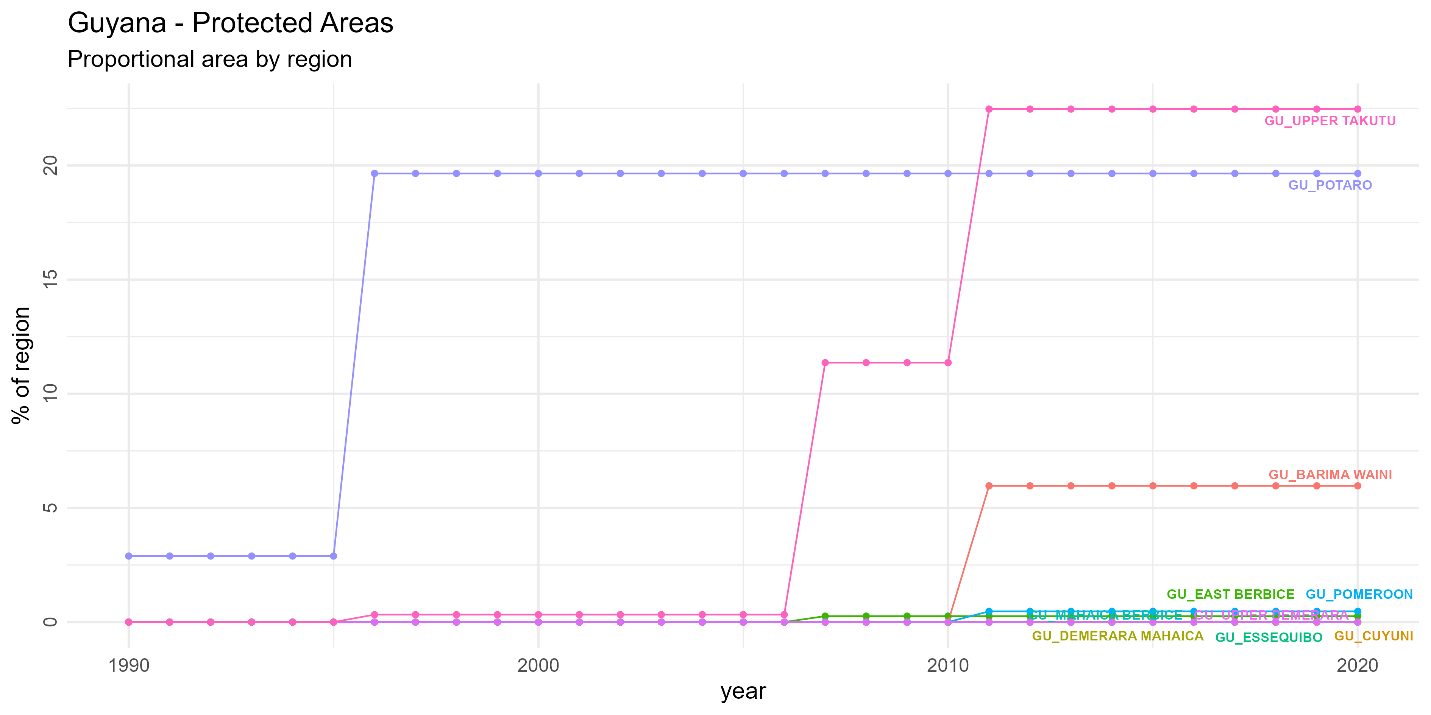 |

**Figure S 46.** Protected areas in Guyana. Above, total area in Protected Areas in Guyana by the management categories we defined for the period 1990-2020. Below, total area for all categories by region.

| 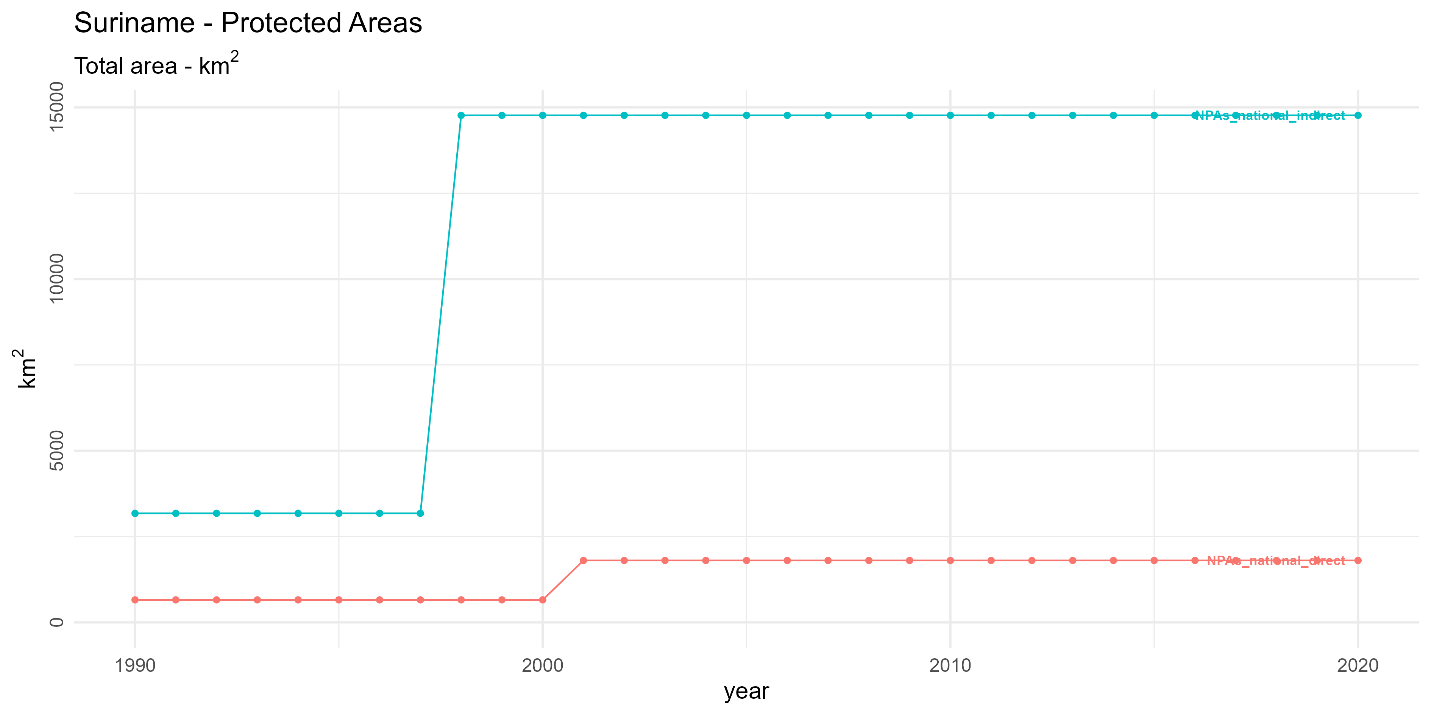 |
| --- |
| 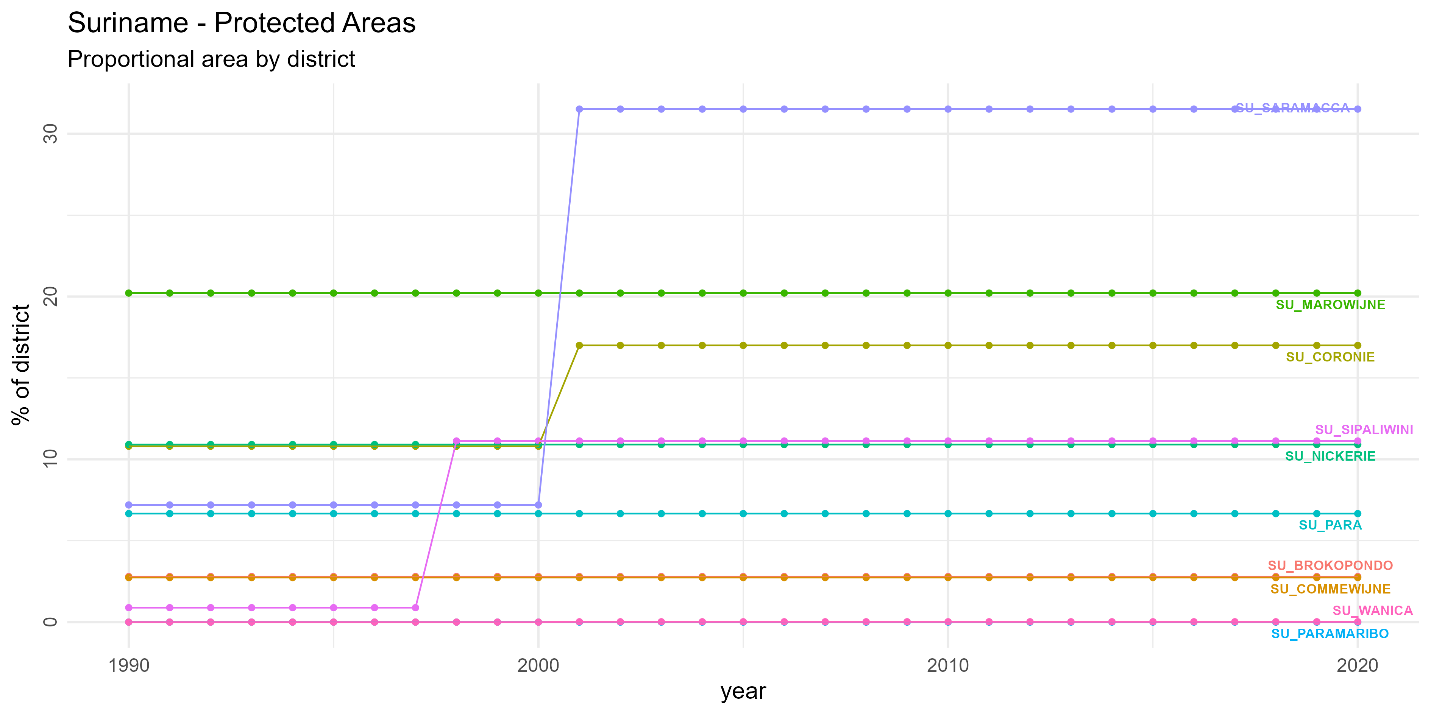 |

**Figure S47. Protected areas in Suriname.** Above, total area in Protected Areas in Suriname by the management categories we defined for the period 1990-2020. Below, total area for all categories by district.


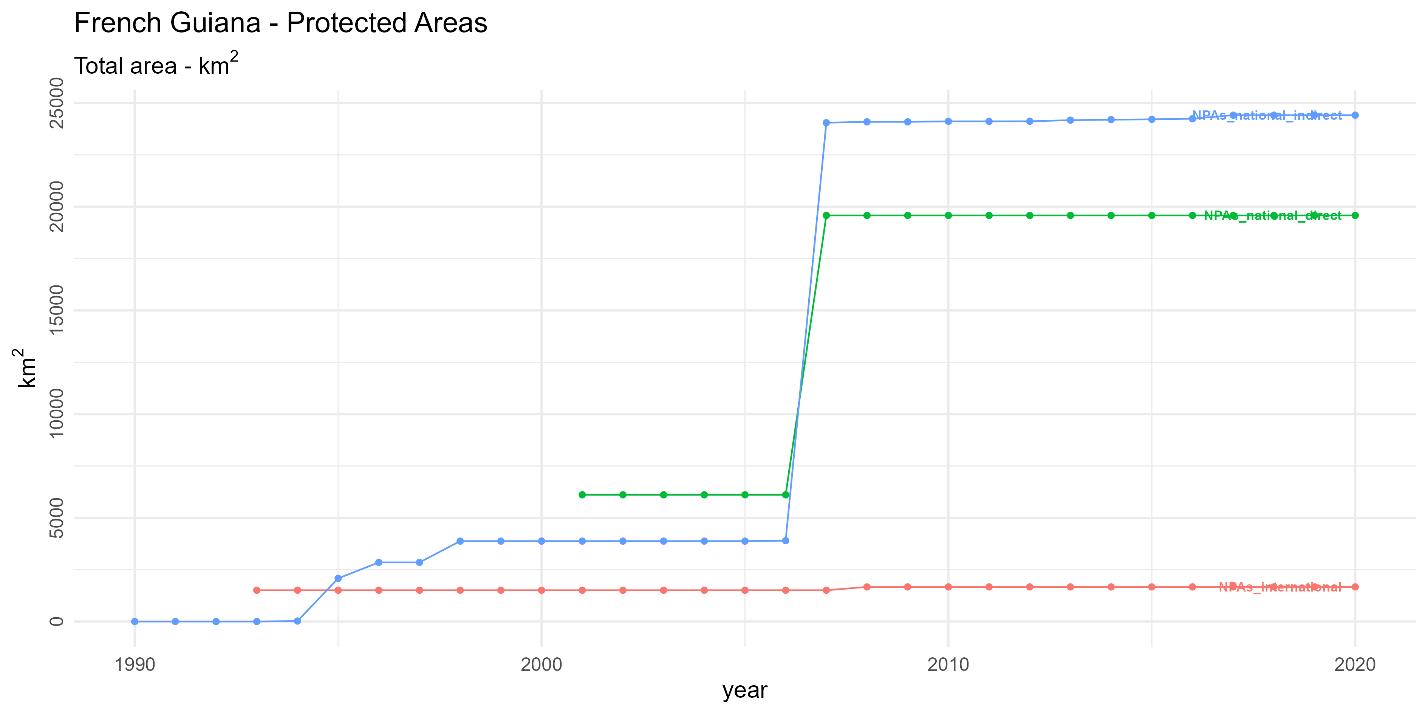


**Figure S48. Protected areas in French Guiana**. Total area in Protected Areas in French Guiana by the management categories we defined for the period 1990-2020.


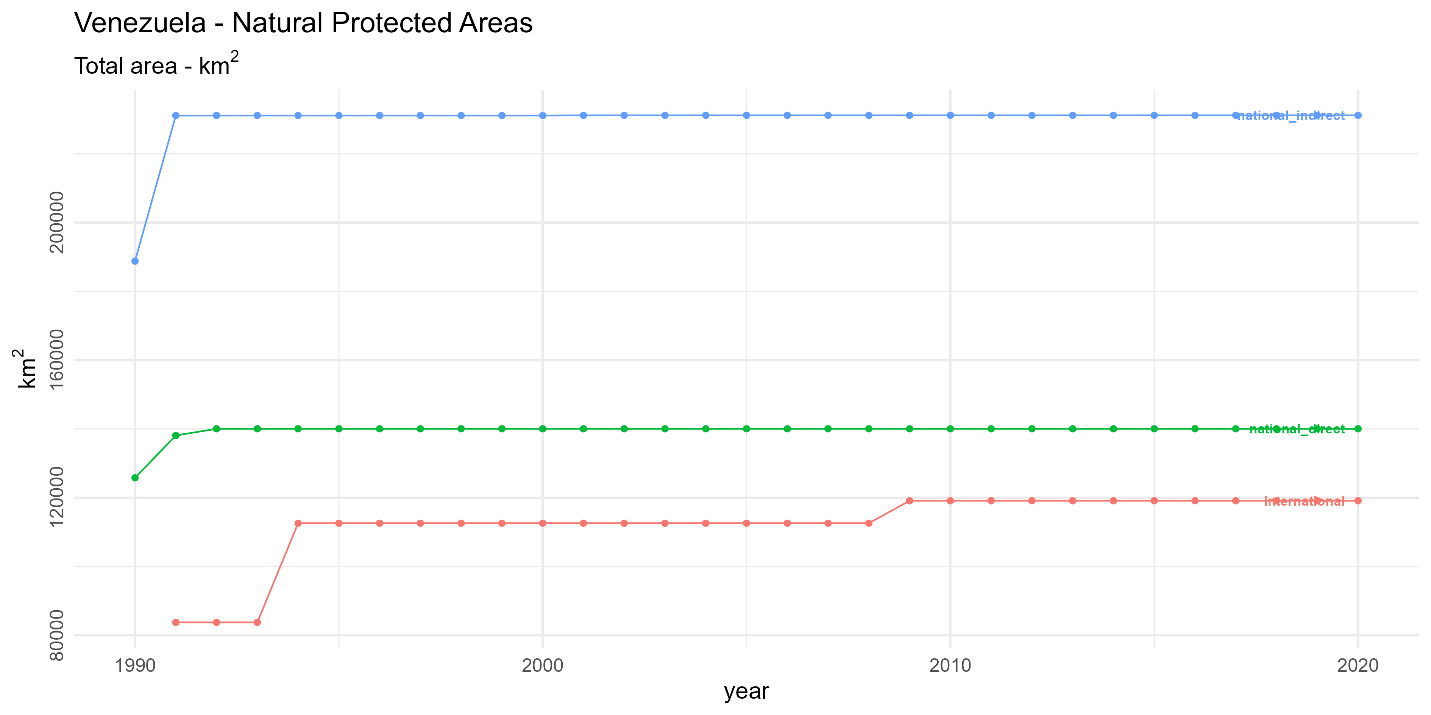


**Figure S 49. Protected areas in Venezuela.** Total area in Protected Areas in Venezuela by the management categories we defined for the period 1990-2020.

### Indigenous Territories

| 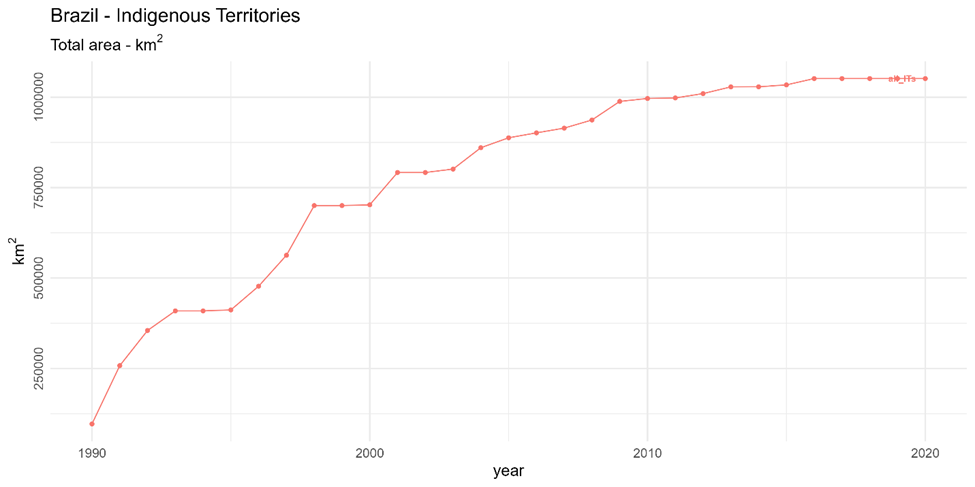 |
| --- |
| 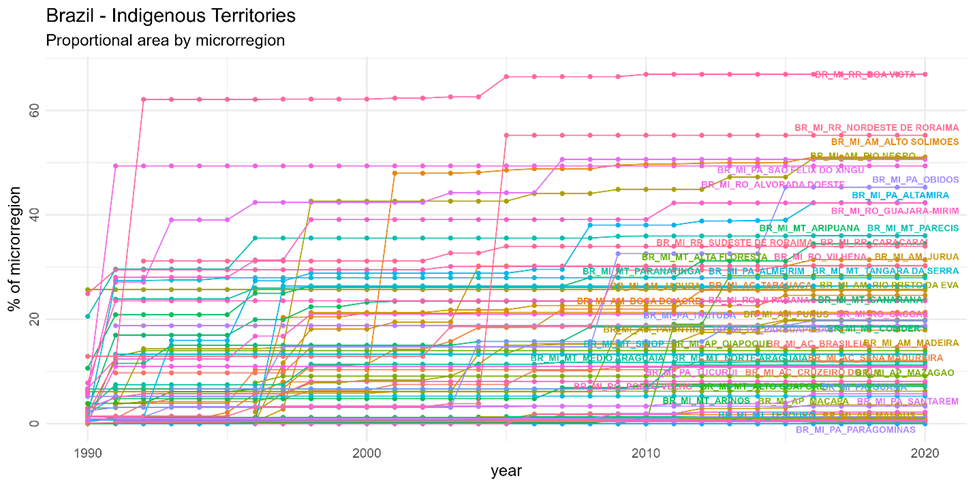 |

**Figure S50. Indigenous Territories in Brazil.** Above, total area in legally recognized indigenous territories for the period 1990-2020. Below, proportional area in legally recognized indigenous territories by microregion.

| 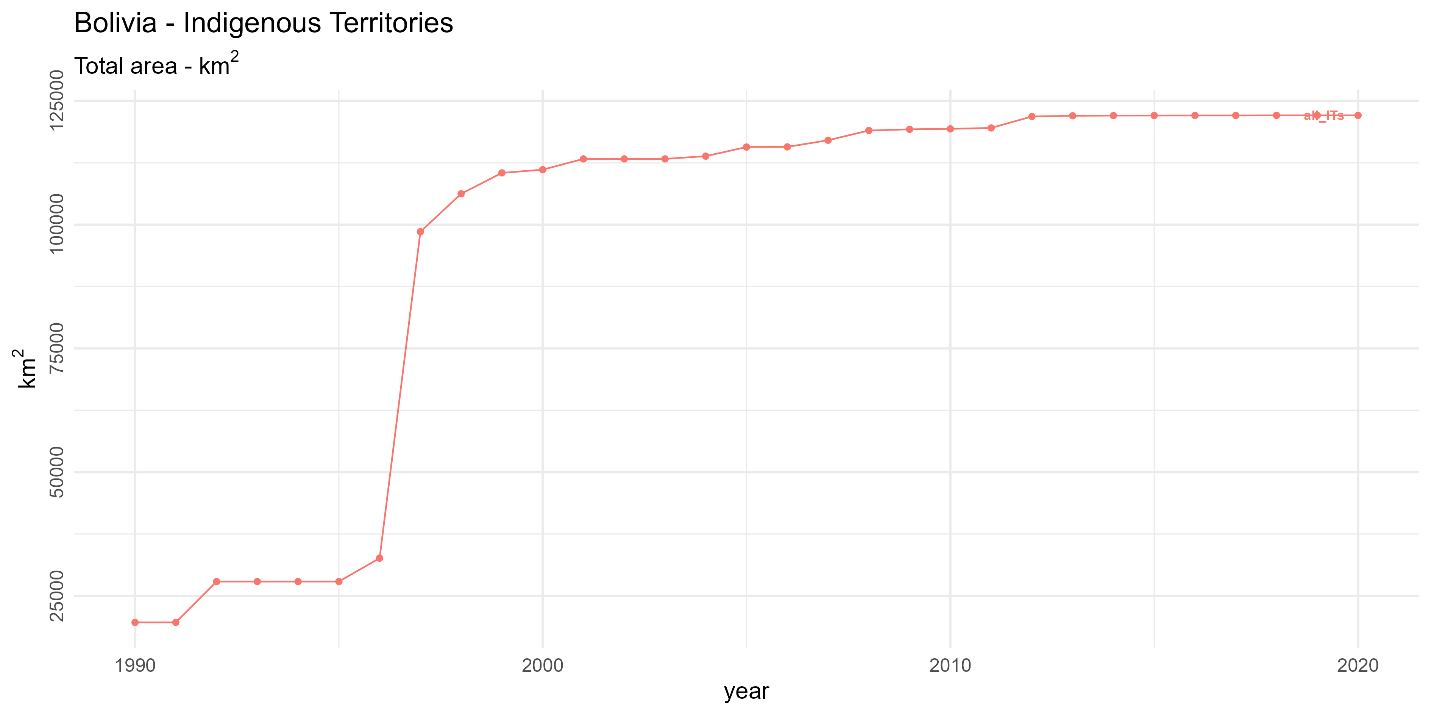 |
| --- |
| 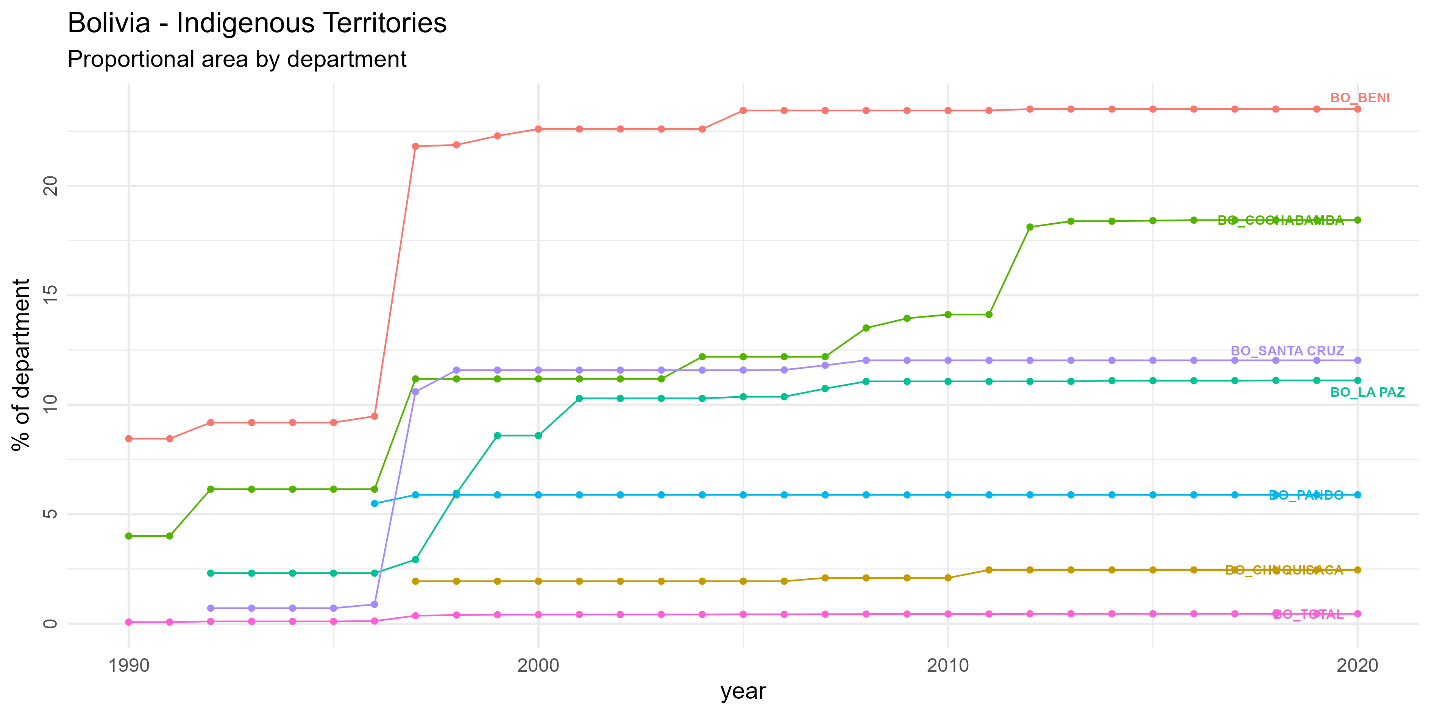 |

**Figure S51. Indigenous Territories in Bolivia.** Above, total area in Indigenous Territories for the period 1990-2020. Below, proportional area in legally recognized indigenous territories by department.

| 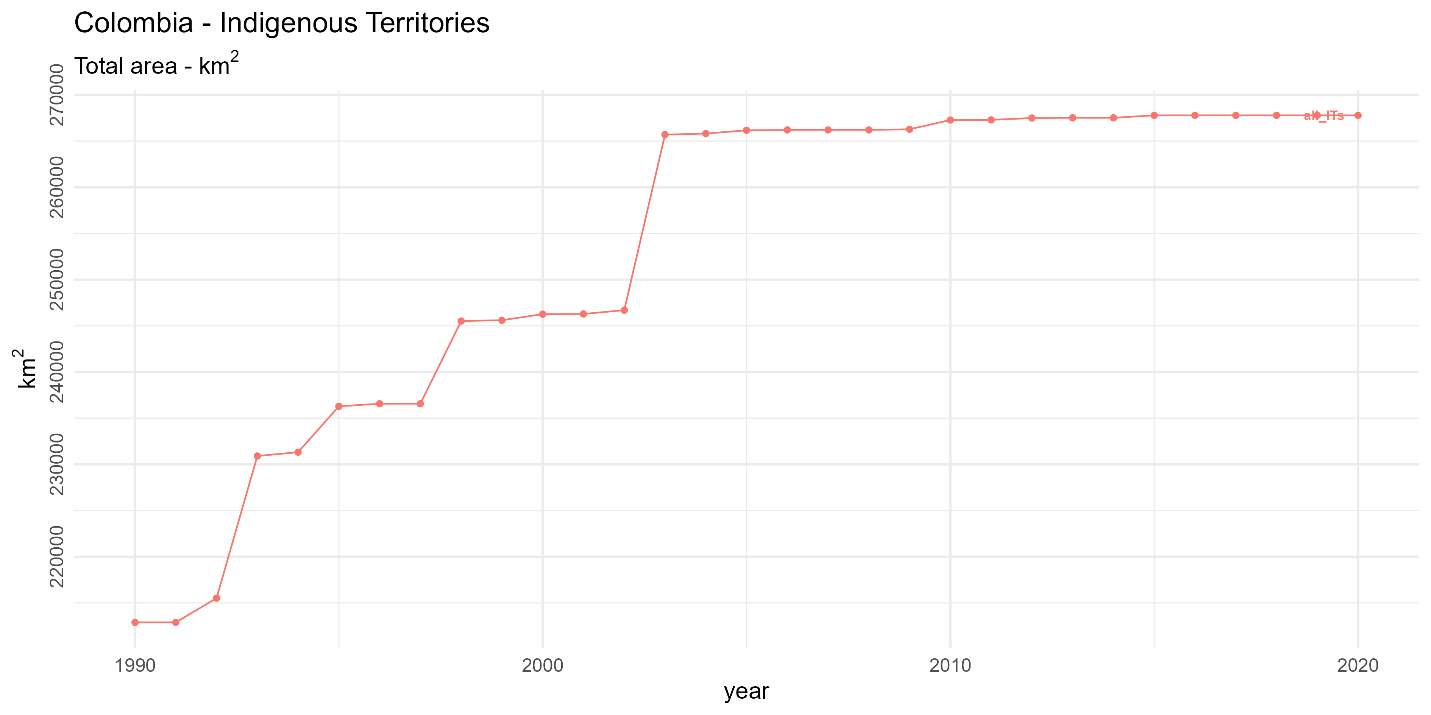 |
| --- |
| 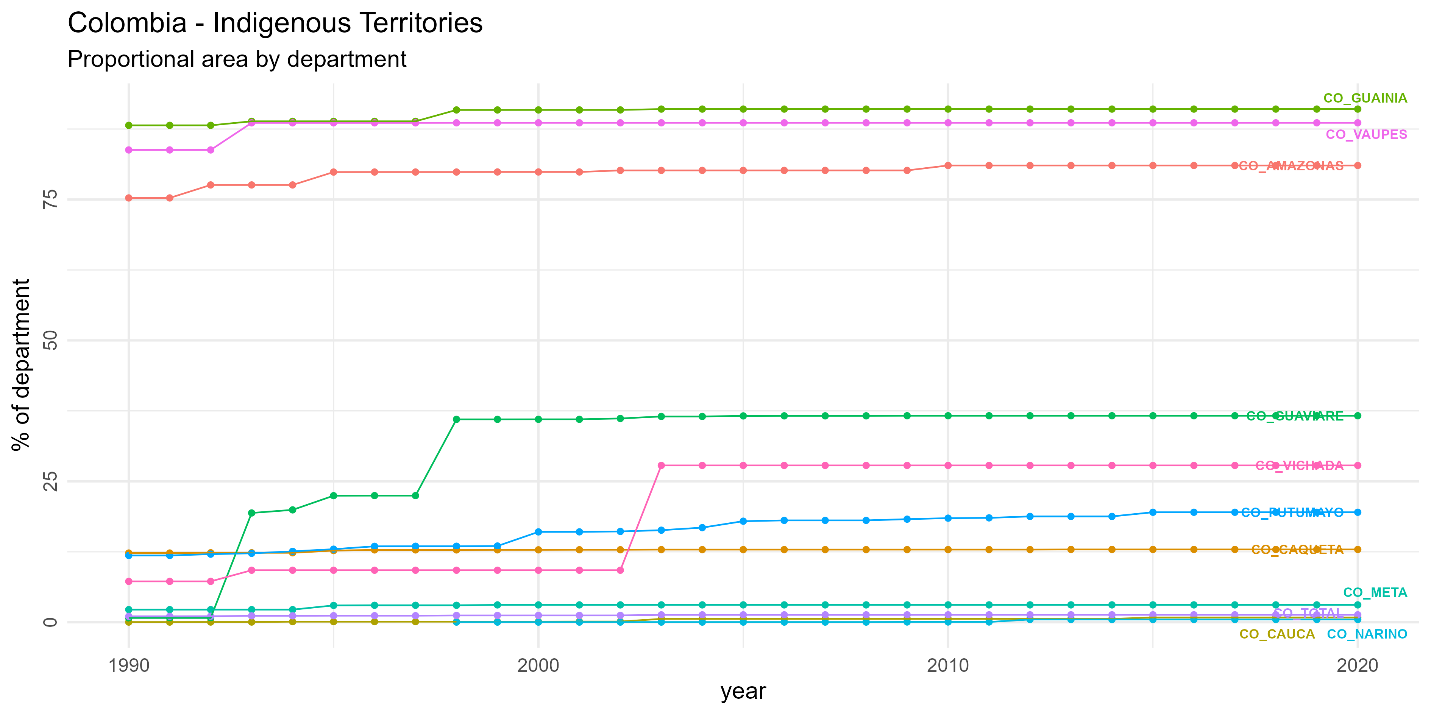 |

**Figure S52. Indigenous Territories in Colombia.** Above, total area in Indigenous Territories for the period 1990-2020. Below, proportional area in legally recognized indigenous territories by department.


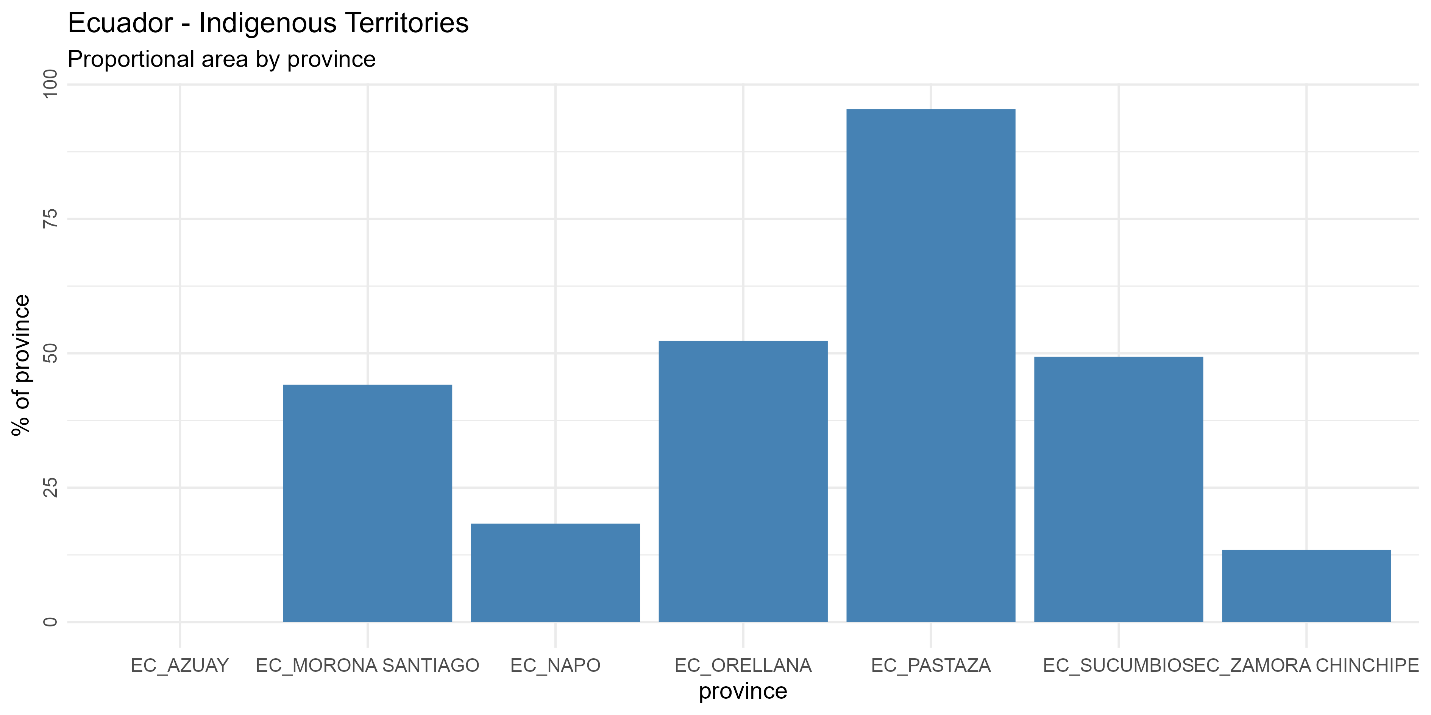


**Figure S53. Indigenous Territories in Ecuador.** Proportional area in legally recognized indigenous territories by province in Ecuador for the year 2020.

| 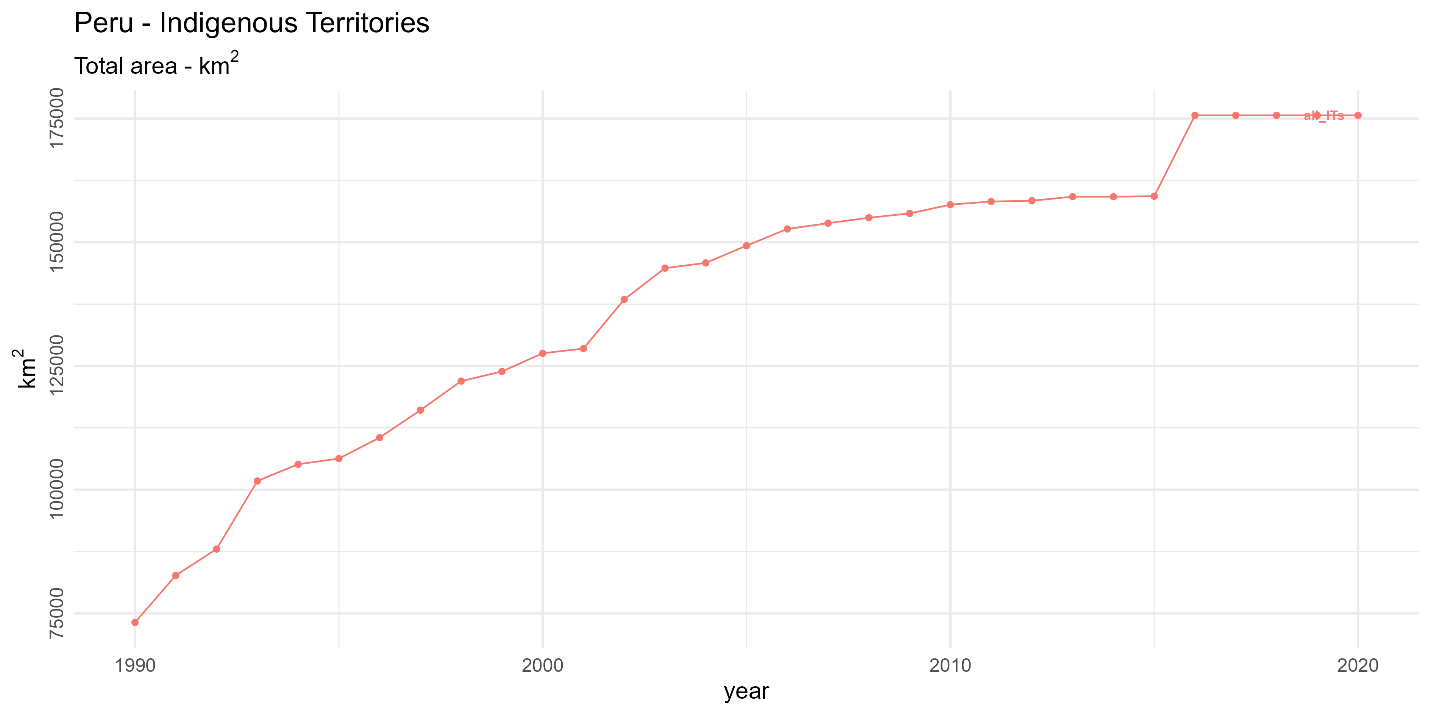 |
| --- |
| 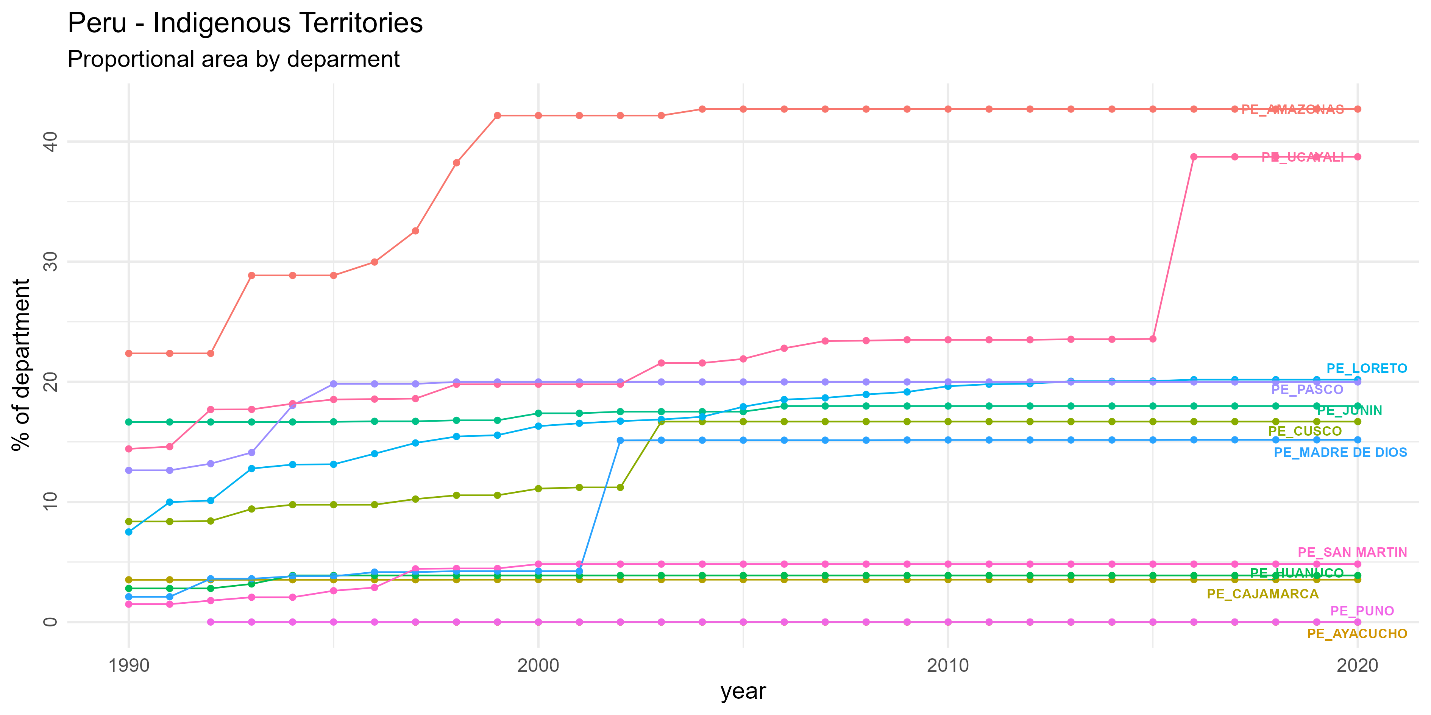 |

**Figure S54. Indigenous Territories in Peru.** Above, total area in Indigenous Territories for the period 1990-2020. Below, proportional area in legally recognized indigenous territories by department.


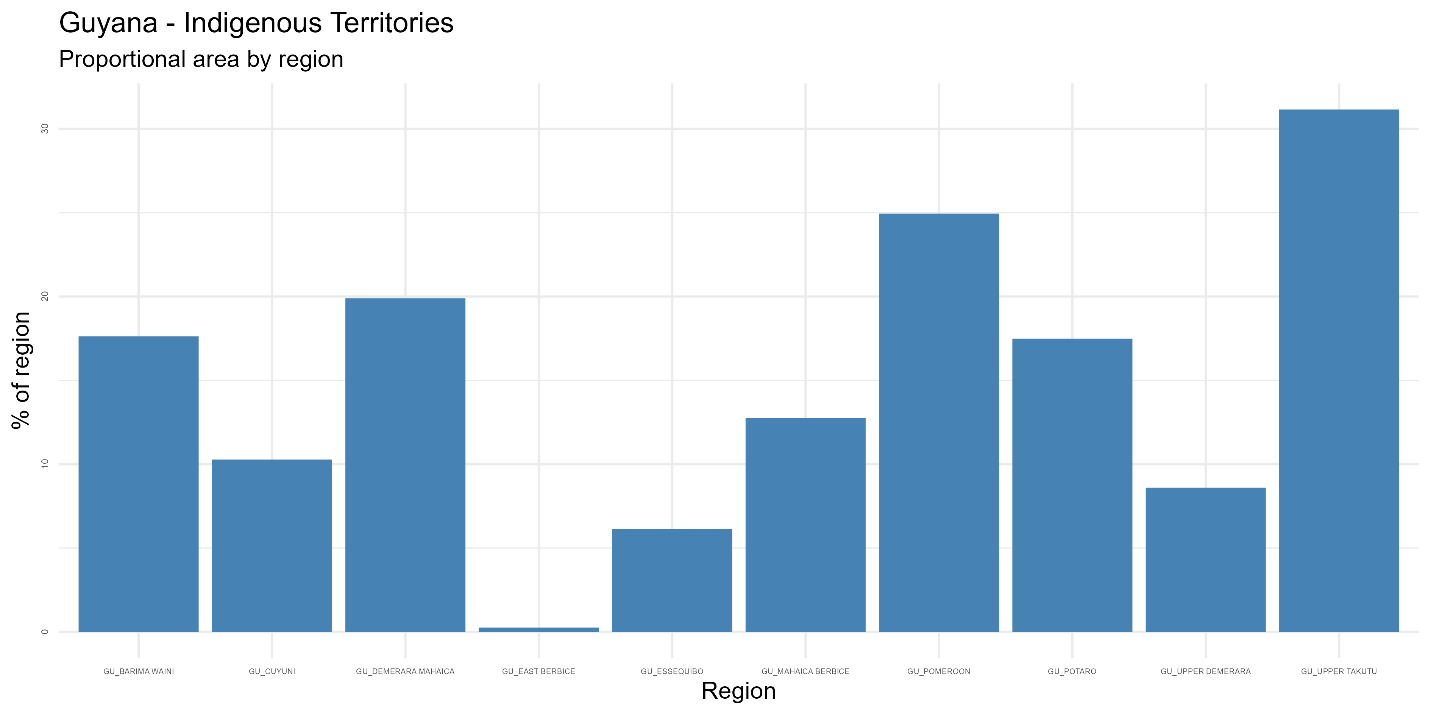


**Figure S55. Indigenous Territories in Guyana.** Proportional area in legally recognized indigenous territories by department in Guyana for the year 2020.


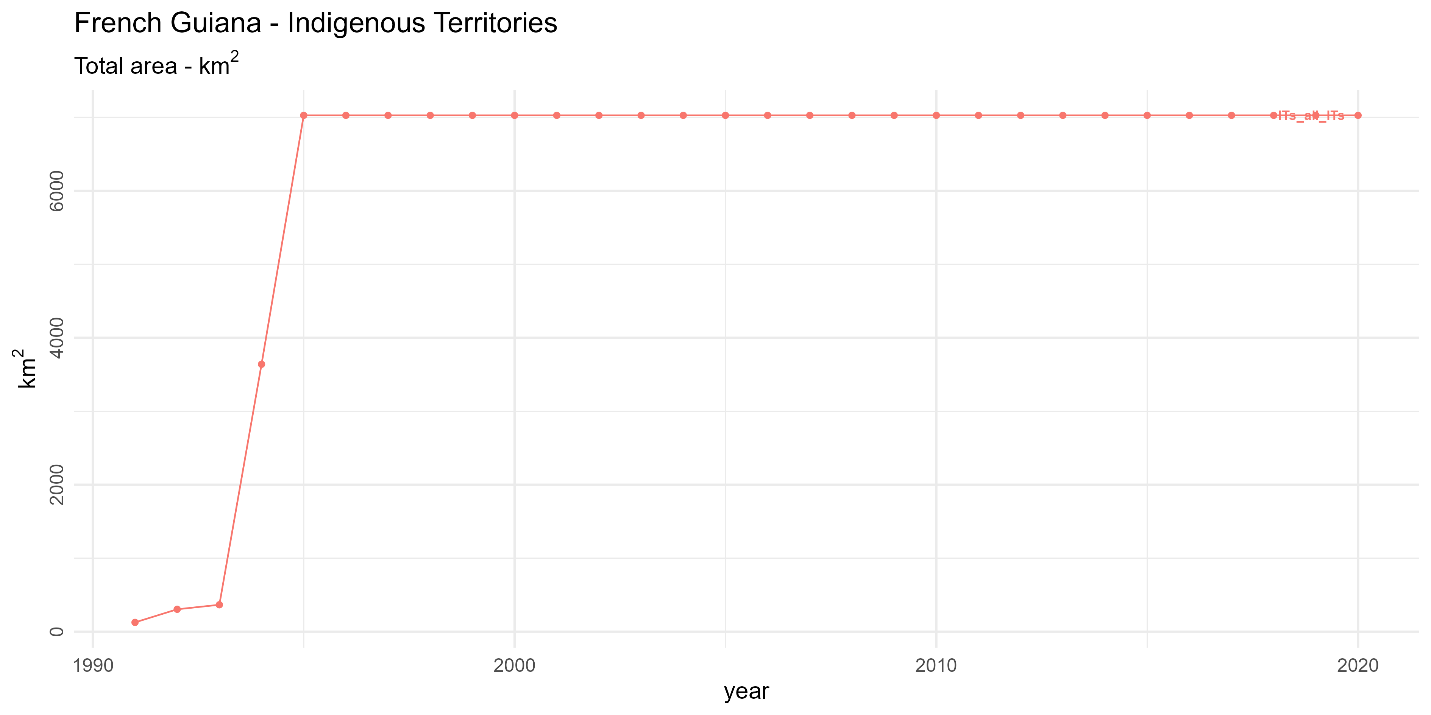


**Figure S56. Indigenous Territories in French Guiana.** Total area in legally recognized indigenous territories in French Guiana for the period 1991-2020.

**Figure S57. Indigenous Territories in Venezuela.** Above, total area in Indigenous Territories for the period 1990-2020. Below, proportional area in legally recognized indigenous territories by State.

# SUPPLEMENTARY MATERIAL C

Biplots from principal components analysis (PCAs) and k-means clusters cross principal components one and two.

## Pan-amazonian

**Figure S58. Pan-amazonian PCA biplot and k-means cluster.** PCA biplot and k-means clustering across components 1 and 2 for the year 1996.

**Figure S59. Pan-amazonian PCA biplot and k-means cluster.** PCA biplot and k-means clustering across components 1 and 2 for the year 2000.

**Figure S60.** **Pan-amazonian PCA biplot and k-means cluster.** PCA biplot and k-means clustering across components 1 and 2 for the year 2010.

**Figure S61.** **Pan-amazonian PCA biplot and k-means cluster.** PCA biplot and k-means clustering across components 1 and 2 for the year 2019.

## Brazil

**Figure S62. Brazil PCA biplot and k-means cluster** **1995.** PCA biplot and k-means clustering across components 1 and 2 for Brazilian microregions for the year 1995.

**Figure S63. Brazil PCA biplot and k-means cluster** **2000.** PCA biplot and k-means clustering across components 1 and 2 for the year 2000.

**Figure S64. Brazil PCA biplot and k-means cluster 2005.** PCA biplot and k-means clustering across components 1 and 2 for the year 2005.

**Figure S65. Brazil PCA biplot and k-means cluster 2010.** PCA biplot and k-means clustering across components 1 and 2 for the year 2010.

**Figure S66. Brazil PCA biplot and k-means cluster 2015.** PCA biplot and k-means clustering across components 1 and 2 for the year 2015.

**Figure S67. Brazil PCA biplot and k-means cluster 2020.** PCA biplot and k-means clustering across components 1 and 2 for the year 2020.

## Bolivia

**Figure S68.** **Bolivia PCA biplot and k-means cluster 1996.** PCA biplot and k-means clustering across components 1 and 2 for the year 1996.

**Figure S69. Bolivia PCA biplot and k-means cluster 2000.** PCA biplot and k-means clustering across components 1 and 2 for the year 2000.

**Figure S70. Bolivia PCA biplot and k-means cluster 2005.** PCA biplot and k-means clustering across components 1 and 2 for the year 2005.

**Figure S71. Bolivia PCA biplot and k-means cluster 2010.** PCA biplot and k-means clustering across components 1 and 2 for the year 2010.

**Figure S72. Bolivia PCA biplot and k-means cluster 2016.** PCA biplot and k-means clustering across components 1 and 2 for the year 2016.

**Figure S73. Bolivia PCA biplot and k-means cluster 2020.** PCA biplot and k-means clustering across components 1 and 2 for the year 2020.

## Colombia

**Figure S74. Colombia PCA biplot and k-means cluster 1995.** PCA biplot and k-means clustering across components 1 and 2 for the year 1995.

**Figure S75. Colombia PCA biplot and k-means cluster 2000.** PCA biplot and k-means clustering across components 1 and 2 for the year 2000.

**Figure S76. Colombia PCA biplot and k-means cluster 2005.** PCA biplot and k-means clustering across components 1 and 2 for the year 2005.

**Figure S77. Colombia PCA biplot and k-means cluster 2010.** PCA biplot and k-means clustering across components 1 and 2 for the year 2010.

**Figure S78. Colombia PCA biplot and k-means cluster 2015.** PCA biplot and k-means clustering across components 1 and 2 for the year 2015.

**Figure S79. Colombia PCA biplot and k-means cluster 2020.** PCA biplot and k-means clustering across components 1 and 2 for the year 2020.

## Ecuador

**Figure S80. Ecuador PCA biplot and k-means cluster 2000.** PCA biplot and k-means clustering across components 1 and 2 for the year 2000.

**Figure S81. Ecuador PCA biplot and k-means cluster 2005.** PCA biplot and k-means clustering across components 1 and 2 for the year 2005.

**Figure S82. Ecuador PCA biplot and k-means cluster 2010.** PCA biplot and k-means clustering across components 1 and 2 for the year 2010.

**Figure S83. Ecuador PCA biplot and k-means cluster 2015.** PCA biplot and k-means clustering across components 1 and 2 for the year 2015.

**Figure S84. Ecuador PCA biplot and k-means cluster 2020.** PCA biplot and k-means clustering across components 1 and 2 for the year 2000.

## Peru

**Figure S85. Peru PCA biplot and k-means cluster 1995.** PCA biplot and k-means clustering across components 1 and 2 for the year 1995.

**Figure S86. Peru PCA biplot and k-means cluster 2000.** PCA biplot and k-means clustering across components 1 and 2 for the year 2000.

**Figure S87. Peru PCA biplot and k-means cluster 2005.** PCA biplot and k-means clustering across components 1 and 2 for the year 2005.

**Figure S88. Peru PCA biplot and k-means cluster 2010.** PCA biplot and k-means clustering across components 1 and 2 for the year 2010.

**Figure S89.** **Peru PCA biplot and k-means cluster 2016.** PCA biplot and k-means clustering across components 1 and 2for the year 2016.

**Figure S90. Peru PCA biplot and k-means cluster 2019.** PCA biplot and k-means clustering across components 1 and 2 for the year 2019.

# SUPPLEMENTARY MATERIAL D

This section contains additional information about the comparison of the dataset presented with the data provided by MapBiomas. For this analysis we used the 5.0 version excel file available at <https://amazonia.mapbiomas.org/estadisticas/> (MapBiomas 2024), accessed on 5.21.2024.

The methodology is detailed at <https://amazonia.mapbiomas.org/atbd-entienda-cada-etapa/>. The data from MapBiomas was produced through satellite images analyses but we used the data tables with areas already aggregated by subnational jurisdictions, as the data we present. We only selected for analysis the jurisdictions that are contained in both sources. We compared agricultural area, oil palm area, pastures in Ecuador and mining area. In the case of mining, the data provided by MapBiomas corresponds to land under mining, whereas the data we present are areas of mining concessions.

Some trends are very similar between both datasets. For instance, the increase in mining since the mid-2000s emerges in both datasets. Similarly, Guyana, Suriname and Venezuela also have the largest proportional areas under mining or within mining concessions, although with different ranking.

The trends in agricultural data also share some similarities. Ecuador, Bolivia and Brazil had the largest proportional areas, although the proportional area in Ecuador reported in the dataset we present has a large spike between 2012 and 2013, which largely follows the trajectory of the changes in pastures. Despite this anomaly, both datasets report an increase in pasture area in Ecuador. Both datasets also report large increases in oil palm area in most countries. Nevertheless, MapBiomas reports a substantially lower area in Colombia than the data we collected.

**Figure S91**. Total agricultural area reported by MapBiomas and the data collected in this work. The top row shows the total area in hectares and the bottom row the total proportional area in each country.

**Figure S92.** Total oil palm and pastures area reported by MapBiomas and the data collected in this work. The top row shows the total oil palm area and the bottom row the total pasture area for Ecuador.

**Figure S93.** Area under mining and mining concessions area. Left column shows area under mining per country reported by MapBiomas, right column shows area of mining concessions from the dataset presented. Top row shows absolute area and bottom row proportional area.

# SUPPLEMENTARY MATERIAL E

This supplementary material shows the plots of drivers and interventions aggregated by the archetypes present at 2020.

**Figure S94.** Population growth by archetypes. These plots show the changes in population growth rates aggregated by the subnational land use archetypes that emerge by 2020.

**Figure S95.** Total population by archetypes. These plots show the changes in total population aggregated by the subnational land use archetypes that emerge by 2020.

**Figure S96.** Total cattle population by archetypes. These plots show the changes in total cattle population aggregated by the subnational land use archetypes that emerge by 2020.

**Figure S97.** Total mining concessions’ area by archetypes. These plots show the mining concessions’ area aggregated by the subnational land use archetypes that emerge by 2020. No dates of concession granting were found for Bolivia, so the plot only shows the total area by archetype by 2017.

**Figure S98.** Total oil blocks’ area by archetypes. These plots show the oil blocks’ area aggregated by the subnational land use archetypes that emerge by 2020. No dates of blocks granting were found for Ecuador, so the plot only shows the total area by archetype by 2017.

**Figure S99.** Total protected areas’ area by archetypes. These plots show the protected areas’ area aggregated by the subnational land use archetypes that emerge by 2020.

**Figure S100.** Total Indigenous Territories’ area by archetypes. These plots show the Indigenous Territories’ area aggregated by the subnational land use archetypes that emerge by 2020. No dates of Territories’ legal recognition were found for Ecuador, so the plot only shows the total area by archetype by 2017.

# SUPPLEMENTARY MATERIAL F

This supplementary material contains plots showing the results from the Mann-Kendall analyses. We analysed the trends of the main crops (soy, corn, rice, oil palm, cacao, coffee, sugarcane and banana), cattle, population growth and extractive activities at the archetypes that had emerged by 2020.

In the plots we show variables on the x axes and Kendall’s tau on the y axes. Kendall’s tau represents the strength and direction of monotonic trends. Statistical significance is also tested for each trend.

In the lollipop plots below, lines extending to the right represent increasing trends, lines extending to the left show decreasing trends, and grey lines represent variables that had non-significant trends.

# REFERENCES

Agência Nacional de Águas - Coordenação de Conjuntura e Gestão da Informação, and Ministério do Meio Ambiente. 2019. Unidades de Conservação. *Catálogo de Metadados da ANA*.

Agencia Nacional de Minería - ANM. n.d. CMC - Catastro Minero Colombiano.

Agência Nacional do Petróleo, Gás Natural e Biocombustíveis - ANP. 2023. CPL - Sistema de Consultas Públicas ANP.

Amazon Geo-Referenced Socio-Environmental Information Network. 2020. Maps & Publications. *RAISG*.

Browder, J., M. Pedlowski, R. Walker, R. Wynne, P. Summers, A. Abad, N. Becerra-Cordoba, and J. Mil-Homens. 2008. Revisiting Theories of Frontier Expansion in the Brazilian Amazon: A Survey of the Colonist Farming Population in Rondônia’s Post-Frontier, 1992-2002. *World Development* 36: 1469–1492. doi:10.1016/j.worlddev.2007.08.008.

Bureau of Statistics. 2019. Data Tables - Bureau of Statistics. October 18.

Canelas, J. C. R., H. Z. Taborga, and G. J. A. Escalante. 2005. *Migraciones a Pando y su contribución al desarrollo regional*. FUNDACION PIEB.

City Population. n.d. Suriname: Districts, Cities & Urban Communes - Population Statistics, Maps, Charts, Weather and Web Information.

city-facts. 2023. Suriname.

Delvoye, K., M. Parahoe, and H. Libretto. 2017. Suriname: An Exposed Interior. In *The 21st Century Fight for the Amazon: Environmental Enforcement in the World’s Biggest Rainforest*. Springer.

Departamento Administrativo Nacional de Estadística – DANE. 2022. Demografía y población. *Departamento Administrativo Nacional de Estadística – DANE*. https://www.dane.gov.co/index.php/estadisticas-por-tema/demografia-y-poblacion. Accessed February 23.

Dirección de Estadísticas Agropecuarias y Ambientales. 2023a. Encuesta de Superficie y Producción Agropecuaria Continua (ESPAC) - Metodología. Instituto Nacional de Estadística y Censos.

Dirección de Estadísticas Agropecuarias y Ambientales. 2023b. Evolución Histórica de la Encuesta de Superficie y Producción Agropecuaria Continua (ESPAC) 2022. Instituto Nacional de Estadística y Censos.

Dirección General de Estadística, Seguimiento y Evaluación de Políticas. 2020. *Anuario Estadístico de la Producción Ganadera y Avícola 2020*. Ministerio de Desarrollo Agrario y Riego.

Dirección Gral. de Biodiversidad y Areas Protegidas, and Servicio Nacional de Areas Protegidas. 2012. Áreas Protegidas Subnacionales en Bolivia. Situación actual 2012. Ministerio de Medio Ambiente y Agua (MMAyA).

ESRI. 2021a. Catastro Minero Ecuador.

ESRI. 2021b. LargeScale_concession.

ESRI. 2021c. Lotes petroleros.

ESRI. 2021d. Medium Scale Mineral Properties (Concessions).

ESRI. 2021e. Petróleo Llanos orientales.

ESRI. 2021f. runap - Registro Unico Nacional AP.

ESRI. 2021g. Small Mining Claims.

ESRI. 2021h. Suriname_Mining.

ESRI. 2022. SNAP.

Estado Peruano. 2022a. Servicio Nacional de Áreas Naturales Protegidas por el Estado - Reservas Comunales. *Plataforma digital única del Estado Peruano*.

Estado Peruano. 2022b. Zonas Reservadas - Servicio Nacional de Áreas Naturales Protegidas por el Estado. *GOB.PE: plataforma digital única del Estado Peruano.*

European Commission. Joint Research Centre. 2023. *GHSL data package 2023.* LU: Publications Office of the European Union.

Eva, H. D., and O. Huber, ed. 2005. *A Proposal for defining the geographical boundaries of Amazonia*. European Commission Joint Research Centre.

Fundación Jubileo. n.d. Sistema de Información Geográfico de Hidrocarburos.

García Peña, R. E., and M. I. Silva Viera. 2013. Las ABRAE versus las áreas protegidas en Venezuela. *Revista COPÉRNICO* 10: 13.

García Peña, R. E., L. Hernández, M. I. Silva Viera, Y. Sumoza, M. Rodríguez, and J. R. García. 2019. SHORT COMMUNICATION REMARKS ON THE VENEZUELAN PROTECTED AREAS SYSTEM AS LISTED ON THE WORLD DATABASE ON PROTECTED AREAS AND CBD AICHI TARGET 11. *PARKS* 25.

Governo Federal, and Agência Nacional de Mineração. 2021. Sistema de Informações Geográficas da Mineração (SIGMINE). *Portal Brasileiro de Dados Abertos*.

Institut national de la statistique et des études économiques. 2023. Population estimates - All - French Guiana.

Instituto Brasileiro de Geografia e Estatística. 2021a. Estimates of resident population for Municipalities and Federation Units | IBGE. https://www.ibge.gov.br/en/statistics/social/population/18448-estimates-of-resident-population-for-municipalities-and-federation-units.html?=&t=o-que-e. Accessed November 4.

Instituto Brasileiro de Geografia e Estatística. 2021b. Tabela 1612: Área plantada, área colhida, quantidade produzida, rendimento médio e valor da produção das lavouras temporárias. https://sidra.ibge.gov.br/tabela/1612. Accessed November 4.

Instituto Brasileiro de Geografia e Estatística. 2021c. Tabela 1613: Área destinada à colheita, área colhida, quantidade produzida, rendimento médio e valor da produção das lavouras permanentes. https://sidra.ibge.gov.br/tabela/1613. Accessed November 4.

Instituto Brasileiro de Geografia e Estatística - IBGE. 2014. *Manual Técnico das Pesquisas Agropecuárias Municipais*. Rio de Janeiro: Instituto Brasileiro de Geografia e Estatística - IBGE.

Instituto Brasileiro de Geografia e Estatística - IBGE. n.d. Tabela 3939: Efetivo dos rebanhos, por tipo de rebanho.

Instituto Chico Mendes de Conservação da Biodiversidade. 2020a. Mapa Temático e Dados Geoestatísticos das Unidades de Conservação Federais. *Instituto Chico Mendes de Conservação da Biodiversidade*.

Instituto Chico Mendes de Conservação da Biodiversidade. 2020b. Unidade de Conservação. *Instituto Chico Mendes de Conservação da Biodiversidade*.

Instituto Chico Mendes de Conservação da Biodiversidade. n.d. ICMBio - SIMRPPN.

Instituto Geológico Minero y Metalúrgico -INGEMMET. n.d. Geocatmin.

Instituto Nacional de Estadística - INE. 2020. *Ficha Técnica - ESTADÍSTICAS BÁSICA AGRÍCOLA*. Instituto Nacional de Estadística - INE.

Instituto Nacional de Estadística - INE. 2022a. Agricultura - Cuadros Estadísticos. *INE*.

Instituto Nacional de Estadística - INE. 2022b. Censos. *INE*.

Instituto Nacional de Estadística - INE. 2022c. Ganadería - Cuadros Estadísticos. *INE*.

Instituto Nacional de Estadística - INE. 2022d. Longitud de Caminos. *INE*.

Instituto Nacional de Estadística - INE. 2022e. Población y Hechos Vitales. *INE*.

Instituto Nacional de Estadística de Venezuela. 2011. Proyecciones de Población. *Instituto Nacional de Estadística*.

Instituto Nacional de Estadística e Informática - INEI. 2022. PERU Instituto Nacional de Estadística e Informática - INEI. https://www.inei.gob.pe/estadisticas/indice-tematico/poblacion-y-vivienda/. Accessed February 23.

Instituto Nacional de Estadística e Informática - INEI. n.d. IV Censo Nacional Agropecuario 2012 - Cuadros Estadísticos.

Instituto Nacional de Estadística e Informática - INEI. n.d. SERIES NACIONALES.

Instituto Nacional de Estadística y Censos. 2022. Censo Nacional Agropecuario. *Instituto Nacional de Estadística y Censos*. https://www.ecuadorencifras.gob.ec/censo-nacional-agropecuario/. Accessed August 22.

Instituto Nacional de Estadística y Censos. n.d. Encuesta de Producción Agropecuaria Continua. *Instituto Nacional de Estadística y Censos*.

Instituto Nacional de Estadística y Censos, and Ministerio de Agricultura y Ganadería. 2002. III Censo Nacional Agropecuario - Resultados Nacionales.

International Work Group for Indigenous Affairs. 2021. The Indigenous World 2021: Suriname - IWGIA - International Work Group for Indigenous Affairs.

Iwokrama International Centre for Rainforest Conservation and Development. 2020. Iwokrama International Centre for Rainforest Conservation and Development - About Us. *Iwokrama International Centre for Rainforest Conservation and Development*.

MapBiomas. 2024. MapBiomas Amazonia - ESTADÍSTICAS.

Ministério da Infraestrutura. 2021. Mapas e Bases dos Modos de Transportes. *Ministério da Infraestrutura*.

Ministerio de Agricultura y Desarrollo Rural. 2018a. Evaluaciones Agropecuarias - EVA y Anuario Estadístico del Sector Agropecuario. *Red de Información y Comunicación del Sector Agropecuario Colombiano - Agronet*.

Ministerio de Agricultura y Desarrollo Rural. 2018b. Inventario pecuario. *Red de Información y Comunicación del Sector Agropecuario Colombiano - Agronet*.

Ministerio de Desarrollo Agrario y Riego. 2021. Compendio anual de “PRODUCCIÓN AGRÍCOLA.”

Ministério de Minas e Energia. n.d. Shapefile de Dados. *Agência Nacional do Petróleo, Gás Natural e Biocombustíveis*.

Ministerio del Ambiente. 2015. Info SNAP | Sistema Nacional de Áreas Protegidas del Ecuador. *Sistema Nacional de Áreas Protegidas del Ecuador*.

Ministerio del Ambiente, Agua y Transición Ecológica. 2018. Tambillo, primera área protegida comunitaria del Ecuador – Ministerio del Ambiente, Agua y Transición Ecológica. *Ministerio del Ambiente, Agua y Transición Ecológica - Noticias*. May 3.

Ministerio del Ambiente, Agua y Transición Ecológica. 2020. Ministerio declara a los páramos de Ichubamba Yasepan como la segunda área protegida privada y la número 60 del país – Ministerio del Ambiente, Agua y Transición Ecológica. *Ministerio del Ambiente, Agua y Transición Ecológica - Noticias*. July 30.

Ministerio del Ambiente del Ecuador. 2016. *Estrategia Nacional de Biodiversidad 2015-2030*. primera edición. Quito, Ecuador: Ministerio del Ambiente del Ecuador.

Ordóñez, J. D. 2018. EL RÉGIMEN DE LAS ÁREAS NATURALES PROTEGIDAS EN PERÚ. *Monografías de la Revista Aragonesa de Administración Pública* 17: 385–403.

Ouboter, P. E. 2002. Directory of Protected Areas of Suriname. IBER On behalf of Conservation International Suriname.

Parques Nacionales Naturales de Colombia. 2021a. Registro Único Nacional de Áreas Protegidas – RUNAP - Acerca de Runap.

Parques Nacionales Naturales de Colombia. 2021b. Registro Único Nacional de Áreas Protegidas – RUNAP - Cifras.

Presidência da República. 2000. *LEI No 9.985, DE 18 DE JULHO DE 2000.*

Protected Areas Trust (Guyana). 2022a. Kanashen Amerindian Protected Area. *Protected Areas Trust (Guyana)*.

Protected Areas Trust (Guyana). 2022b. Protected Areas Trust (Guyana). *Protected Areas Trust (Guyana)*.

Protected Areas Trust (Guyana). 2022c. Protected Areas Trust (Guyana) - About Us. *Protected Areas Trust (Guyana)*.

Randell, H. F., and L. K. VanWey. 2014. Networks Versus Need: Drivers of Urban Out-Migration in the Brazilian Amazon. *Population Research and Policy Review* 33: 915–936. doi:10.1007/s11113-014-9336-7.

Secretaría Nacional de Planificación. 2021. Proyecciones y Estudios Demográficos - Sistema Nacional de Información. *Sistema Nacional de Información*.

Servicio Nacional de Áreas Naturales Protegidas por el Estado - SERNANP. 2022. GEO ANP - VISOR DE LAS ÁREAS NATURALES PROTEGIDAS. https://geo.sernanp.gob.pe/visorsernanp/. Accessed February 8.

Servicio Nacional de Áreas Protegidas. 2020. SERNAP | Presentación.

Servicio Nacional de Áreas Protegidas (SERNAP). 2012. Plan de Acción para la Implementación del Programa de Trabajo sobre Áreas Protegidas de la Convención sobre la Diversidad Biológica. ESTADO PLURINACIONAL DE BOLIVIA.

Siso Quintero, G. J. 2012. La población de Venezuela: evolución, crecimiento y distribución geográfica. *Terra Nueva Etapa* 28: 33.

Sistema Nacional de Áreas Protegidas del Ecuador, and Ministerio del Ambiente. 2015. Bosques Protectores | Sistema Nacional de Áreas Protegidas del Ecuador.

de Souza, J. L., D. A. de A. Côrte, and L. M. Ferreira. 2012. Perguntas e Respostas sobre RESERVA PARTICULAR DO PATRIMÔNIO NATURAL. Ministério do Meio Ambiente - Instituto Chico Mendes de Conservação da Biodiversidade.

Statistics Division of the Food and Agriculture Organization - United Nations (FAOSTAT). 2022. FAOSTAT.

UNEP-WCMC, and IUCN. 2022. Protected Planet: The World Database on Protected Areas (WDPA) and World Database on Other Effective Area-based Conservation Measures (WD-OECM) [Online]. Cambridge, UK: UNEP-WCMC and IUCN.

United States Census Bureau. 2023. Subnational Population Data by Geographic Area. *Census.gov*.

Vereniging van Inheemse Dorpshoofden in Suriname - VIDS. 2009. *Securing Indigenous Peoples’ Rights in Conservation in Suriname: A review*.
